# PPARG (Pro12Ala) genetic variant and risk of T2DM: a systematic review and meta-analysis

**DOI:** 10.1038/s41598-020-69363-7

**Published:** 2020-07-29

**Authors:** Negar Sarhangi, Farshad Sharifi, Leila Hashemian, Maryam Hassani Doabsari, Katayoun Heshmatzad, Marzieh Rahbaran, Seyed Hamid Jamaldini, Hamid Reza Aghaei Meybodi, Mandana Hasanzad

**Affiliations:** 1 Personalized Medicine Research Center, Endocrinology and Metabolism Clinical Sciences Institute, Tehran University of Medical Sciences, 1411413137 Tehran, Iran; 2 Elderly Health Research Center, Endocrinology and Metabolism Population Sciences Institute, Tehran University of Medical Sciences, 1411413137 Tehran, Iran; 3 Medical Genomics Research Center, Tehran Medical Sciences, Islamic Azad University, 1916893813 Tehran, Iran; 4 Endocrinology and Metabolism Research Center, Endocrinology and Metabolism Clinical Sciences Institute, Tehran University of Medical Sciences, 1411413137 Tehran, Iran

**Keywords:** Genetics, Molecular biology

## Abstract

Type 2 diabetes mellitus (T2DM) is a complex disease caused by the interaction between genetic and environmental factors. A growing number of evidence suggests that the *peroxisome proliferator-activated receptor gamma *(*PPARG*) gene plays a major role in T2DM development. Meta-analysis of genetic association studies is an efficient tool to gain a better understanding of multifactorial diseases and potentially to provide valuable insights into gene-disease interactions. The present study was focused on assessing the association between Pro12Ala variation in the *PPARG* and T2DM risk through a comprehensive meta-analysis. We searched PubMed, WoS, Embase, Scopus and ProQuest from 1990 to 2017. The fixed-effect or random-effect model was used to evaluate the pooled odds ratios (ORs) and 95% confidence intervals (CIs) depending on the heterogeneity among studies. The sources of heterogeneity and publication bias among the included studies were assessed using I^2^ statistics and Egger's tests. A total of 73 studies, involving 62,250 cases and 69,613 controls were included. The results showed that the minor allele (G) of the rs1801282 variant was associated with the decreased risk of T2DM under different genetic models. Moreover, the protective effect of minor allele was detected to be significantly more in some ethnicities including the European (18%), East Asian (20%), and South East Asian (18%). And the reduction of T2DM risk in Ala12 carriers was stronger in individuals from North Europe rather than Central and South Europe. Our findings indicated that the rs1801282 variant may contribute to decrease of T2DM susceptibility in different ancestries.

## Introduction

Type 2 diabetes mellitus (T2DM) is the most common form of diabetes and is described as a highly prevalent multifactorial disorder^1^. According to the recent statistics of the International Diabetes Federation (IDF), the global T2DM epidemic significantly grows at an alarming rate among populations and so it has become a common health problem worldwide^2^. Although T2DM usually affects older adults, it is also gradually seen in children, adolescents and younger adults due to increasing levels of obesity, low physical activity and poor diet^3^. T2DM is recognized as a major cause of morbidity and leads to premature coronary heart disease progression (CHD), stroke, peripheral vascular disease (PVD), renal failure, and amputation^4^. T2DM is characterized by hyperglycemia, impaired insulin secretion (IS) and insulin resistance (IR) that results from the interaction between numerous genes and environmental factors^5,6^. The genetic architecture of complex traits is now to be related to several numbers of causal variants. But, the most important common variants show small to modest effect sizes^7,8^.

Single nucleotide polymorphisms (SNPs), the most common type of genetic variations between individuals, are the key players in precision medicine approach. SNPs are responsible for more than 80 percent of the variation between individuals which makes them ideal for genotype and phenotype association studies. Genetic association studies as powerful approach have identified several SNPs that are significantly associated with T2DM susceptibility^9,10^.

Many Genome-wide association studies (GWASs) identified several candidate genes, including *peroxisome proliferator-activated receptor gamma (PPARG)*, a member of the nuclear hormone receptor superfamily, as susceptible to T2DM loci in Finnish and British/Irish ancestries^11,12^. There is a lot of information about the genetic architecture of T2DM including the high degree of polygenicity and the tiny effect sizes of most genetic risk variants but several obstacles complicate translation process of these novel loci^13^. Therefore, there is a strong need to concentrate more on translational work to make sense of the hundreds of loci associated with common diseases such as T2DM.

Several evidence have demonstrated that the SNPs of *PPARG* (nuclear receptor) have an important role in controlling lipid and glucose metabolism^14,15^. Among them, the missense variant rs1801282 (also known as Pro12Ala) in the exon B leads to an amino acid change from Proline (P) to Alanine (A) have been extensively reviewed in epidemiologic studies.

Many case–control studies have reported that the Pro12Ala (Ala12) variant is associated with protection against T2DM risk in East Asian (Japanese)^16,17^, Greater Middle Eastern^18^, and European ancestries such as Finnish^19^, Czech^20^, and White from Scottish^21^. Conversely, some another studies suggested that the *PPARG* Pro12Ala variant could be considered as a risk marker conferring susceptibility to T2DM in the Russian^22^, South Asian (Kashmiri)^23^ and mixed ancestry of south Africa^24^.

The results of these genetic association studies are not consistently reproducible and the majority of the initial positive genetic associations cannot be replicated in multiple studies. Furthermore, most of the candidate genes and their variants occasionally indicate only minor effects in genetic association studies. The lack of reproducibility may be due to several factors including variability in the ethnicities and small sample size. As some risk loci displayed significant ethnic differences in frequency and/or effect size. Therefore, meta-analysis is required to detect a small or moderate genetic effect of polymorphisms. The meta-analysis of the genetic association studies is considered to be decisive evidence when correctly performed^25^. Based on these contradictory results from different independent studies, a comprehensive meta-analysis seemed to be a good approach to combine the results of various studies on the same topic and to further explain their findings.

The potential association of rs1801282 polymorphism in the entire coding region of the *PPARG* gene and the risk of T2DM was reported in several meta-analysis^26–29^. Ludovico et al. (2007) demonstrated a heterogeneous effect of the Ala allele on lower development of T2DM in Europeans, Asians, and North Americans ^27^. The reduction effect of Ala allele in *PPARG* gene on T2DM risk was also reported in a meta-analysis by Huguenin et al. (2010) in Caucasians^28^. Two independent studies just conducted in the Chinese Han populations did not find any association of Pro12Ala and T2DM risk^26,29^. Moreover, a recent meta-analysis with 14 studies indicated the evidence for Pro12Ala as a susceptibility variant in Caucasian and Chinese populations^30^.

Despite the previous meta-analysis studies, we are trying to provide an updated and stronger result of evaluating the relationship between the Pro12Ala variant and the risk of T2DM by performing a comprehensive meta-analysis in a larger number of studies from different geographic regions and different ethnicity and interpret and compare data obtained.

PPARs play important roles in various metabolic processes so they are considered good targets for the treatment of metabolic syndrome. But the use of PPAR agonists in diabetes treatment which mainly target PPARγ has been met with side effects. Accordingly, a better genomic understanding of the pathogenesis of diabetes may alleviate the side effects of these agents through targeting certain genetic variants^31^.

SNP biomarkers have potentially wide clinical application in precision medicine, including disease predisposition, screening, diagnosis, prognosis, monitoring, and pharmacogenetics. Precision medicine enables for early treatment and decreased morbidity and mortality. It is thus important in the precision medicine field to find the relevance of SNPs to medical practice and the extent of their impacts in healthcare practice. In type 2 diabetes as the known genetic associations explain only 5–10% of the inheritable basis of T2DM so the genetic variants prove useful in disease predisposition.

Therefore, this systematic review and meta-analysis study could be essential for considering the importance of *PPARγ* common genetic variant in the risk and pathogenesis of T2DM.

## Materials and methods

The current review evaluated the association between rs1801282 (Pro12Ala) polymorphism and T2DM risk. This study was carried out following the criteria of the Preferred Reporting Items for Systematic Reviews and Meta-analyses (PRISMA)^32^ and the published PROSPERO research protocol (CRD42017058832).

### Search strategy

A comprehensive literature search was performed on several databases including PubMed, Scopus, Embase, Web of Science (Wos), and ProQuest to collect relevant literature published from January 1990 to October 2017. We found the different synonyms of the related terms for all subjects by using thesauri systems such as Medical Subject Headings (MeSH) and EMBAS subject headings (Emtree). The combination of the following terms was used to design the search strategy: (*“Type 2 Diabetes Mellitus” OR “insulin independent diabetes mellitus” OR “Noninsulin-Dependent Diabetes Mellitus” OR …*)* and *(*“PPAR Gamma”, “Peroxisome Proliferator Activated Receptor Gamma” OR “PPAR G” OR “PPAR-G” OR “PPAR Gamma” OR “PPAR-Gamma” OR …*). The finalized search syntax in PubMed was adjusted in other databases for a comprehensive search (available in Supplementary file 1).

For the selection of possibly relevant studies, the titles and abstracts of the articles were independently screened according to eligibility criteria with two reviewers (MR and MH).

Two reviewers (MR and MH) also reviewed the full-text articles to determine whether the selected articles adapted to the eligibility criteria and could be included/excluded in the final investigation. Conflicting opinions were resolved through further discussion to achieve a consensus. Moreover, the reference lists of all eligible studies were also checked to identify additional potentially relevant literature.

### Eligibility criteria

All selected studies had to fulfill the following inclusion criteria:

1. Studies published in peer-reviewed journals.
2. All case–control studies just conducted on the human that assessed any association between *PPARG* rs1801282 (Pro12Ala) polymorphism and risk of T2DM.
3. The data about the allele or genotype frequencies should be sufficient to calculate the odds ratios (ORs) with the corresponding 95% confidence intervals (CIs) of the polymorphism in both the case and control groups.
4. The control group included people without T2DM.
5. Studies that just published in the English language.
6. Studies that their full text was available.
7. Short communication and brief genetic report with sufficient data.

Following studies were excluded:

(1) Family based association studies, including case reports and case series.

(2) Reviews, meta-analysis, letters, editorial, comments, and conference abstracts.

(3) In vitro, ex vivo or animal studies.

(4) Studies lack sufficient data about allele frequency or data that could not be calculated.

(5) Duplicate publications and redundant studies of duplicated data; for duplicate reports that published by the same authors using the same case series, only the most recent and the one with the largest sample size one was included.

### Data extraction

Two researchers (LH and KH) extracted the following items were selected from all eligible studies using a redesigned form according to the aforementioned inclusion and exclusion criteria:

(1) First author’s names and year of publication

(2) Country of setting or ethnicity of participants

(3) The number of cases and controls

(4) Mean age and body mass index (BMI) of participants

(5) Genotyping method

(6) The genotype and allele frequencies of *PPARG* gene variant in cases and controls

(7) Hardy–Weinberg equilibrium (HWE) was calculated based on genotype frequencies of certain *PPARG* rs1801282 (Pro12Ala) gene polymorphisms in the control group.

The agreement between two researchers (LH and KH) was achieved by a discussion with a third expert person (MH) in the research team.

If there was a lack of genotype information, the reviewers contacted the corresponding author to get the required data.

### Quality assessment

The Newcastle–Ottawa Scale (NOS) was used to assess the quality of included studies^33^.

The following items were selected for the inclusion of the study, including the selection of cases and controls (4 items, 1 point each), comparability between cases and controls (1 item, up to 2 points) and exposure in cases and controls (3 items, 1 point each).

The NOS has a score range of zero to nine, and studies with a rating of 7–9 were presumed to be of high quality, 4–6 as moderate quality, 4 or less was classified as low-quality studies. Quality assessment was also conducted by two investigators independently.

First, any disagreement regarding the quality assessment was resolved by checking and discussion between the two reviewers. If the two authors could not achieve a consensus, then a third reviewer would resolve the cases of conflict.

### Statistical analysis

Within the study, the results were combined using RStudio (3.51). Genetic association studies do not follow a specific model and therefore multiple genetic models need to be investigated^34^. The odds ratio (OR) and 95% confidence interval (CI) were used to assess the association between rs1801282 polymorphism in *PPARG* gene and the risk of T2DM in seven genetic models as follow: allele model (G vs. C), homozygote model (GG vs. CC), heterozygote model (CG vs. CC), additive model (GG vs. CG), dominant model (GG + CG vs. CC), recessive model (GG vs. CC + CG), and co-dominant model (CG vs. CC + GG) which a ‘ ‘C’’ denotes a major allele; ‘ ‘G’’ denotes a minor allele.

The Cochrane Q-test index was used for detecting the existence of heterogeneity between the results of the primary studies^35^ and I-square index (I^2^) determined the degree of the heterogeneity in meta-analysis based on I2 value of 25%, 50%, and 75% were nominally regarded as low, moderate, and high estimates, respectively^36^.

We applied the random effect model (REM) for an inverse variance which was used to calculate the combining of primary results (the pooled OR estimate) if the heterogeneity was significant (P-value of Q-test < 0.05 or I^2^ > 50%). Otherwise, the fixed effects model (FEM) was occupied to assess the primary effect of the genotype^37^.

The agreement on the genotype frequency with HWE in the controls was calculated using the Pearson’s χ2 test for each study.

### Subgroup analysis

Subsequently, subgroup analysis by ancestry categories, BMI (< 25 and ≥ 25 kg/m^2^) and age (30–50 and ≥ 50 years) of participants, and year of publications (before 2005 and equal or more 2005) were carried out to achieve more specific results. Ancestries were categorized to European, Greater Middle Eastern, East Asian, South East Asian, South Asian, Asian unspecified, African American or Afro-Caribbean, Hispanic or Latin American, Native American, Other admixed ancestry, and Other) according to the classifications that is provided in a study by Morales et al. (2018)^38^.

### Sensitivity analysis

Sensitivity analysis was accomplished by removing those studies that did not meet with HWE.

Studies with a very poor quality score (equal to two or three) were also excluded from the meta-analysis to getting possible stronger results. Moreover, the leave-one-out method in the sensitivity analysis was conducted through consecutive excluding only one study in each time to assess the cause of heterogeneity and to determine whether any individual study influences the stability of final results (pooled OR) in meta-analysis.

### Publication bias

We also appraised the fundamental sources of potential publication bias in Egger’s linear regression test and visual inspection of the asymmetry of the Begg funnel plot^39^. If there was publication bias, the Duval and Tweedie trim-and-fill technique was accomplished to explore the impact of the publication bias on the results^40^.

## Results

### Characteristics of the included study

**Figure 1:**
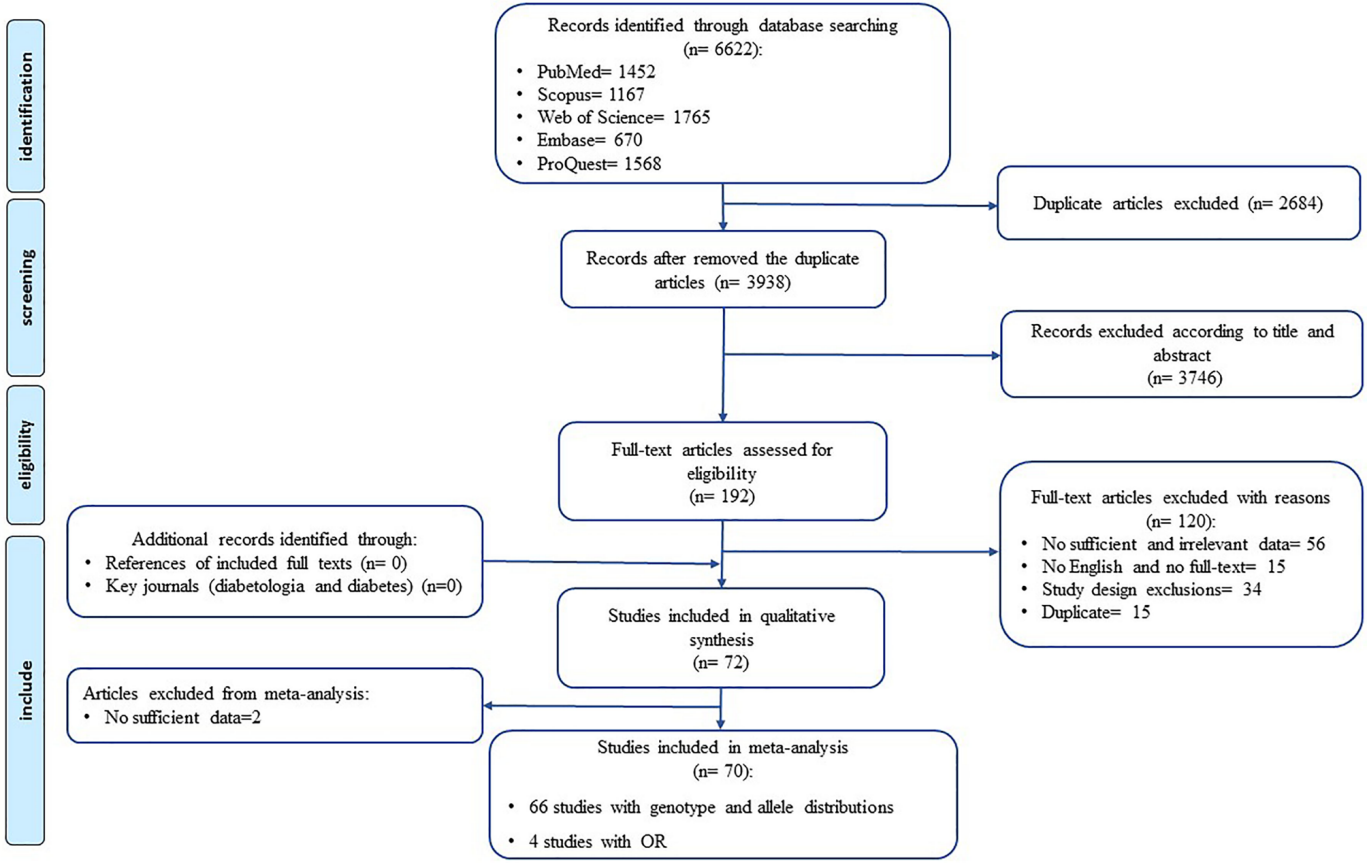
Flow diagram presenting the results of the literature search and study selection process.

During the first stage of our comprehensive search, 6,622 studies were identified through electronic databases and hand searches. As illustrated in Fig. 1, 3,938 articles remained after excluding the duplicates. After reviewing the titles, abstracts of the primary studies, 3,746 papers were identified to be irrelevant. 192 potentially relevant articles were retrieved for further evaluation. Among those remaining studies, 120 studies were excluded for different reasons (56 studies had not sufficient or relevant data about T2DM including studies that evaluated the association of Pro12Ala with type 1 diabetes, metabolite traits, or diabetes complications, also assessed the link of other SNPs and genes with T2DM, and GWAS studies ; 15 studies were not English studies or not available full text; 34 studies were the exclusion of study design such as a clinical trial, cohort, case-series (had no control group), family-based studies, review and meta-analysis, letter to editors/research letters, meeting abstract, commentary, report, news, pilot study; and 15 studies were duplicate) that details are provided in Supplementary Table S1 online.

Two articles due to the insufficient data were removed from further step (meta-analysis). Finally, a total of 73 studies with 62,250 T2DM patients and 69,613 controls met our inclusion criteria for overall meta-analysis after reading the full texts. It should be noted that these 73 studies had been reported by 66 articles.

Besides, other 13 studies data from four articles were lack genotypes or alleles frequency while only OR was reported for these. So we did not exclude from meta-analysis and analyzed using separately command from Stata^41^ that is available in Supplementary file 2 online.

The characteristics and genotype frequencies of included studies are listed in Table 1 and Supplementary file 3 online.

**Table 1:** Characteristics of the studies included in this meta-analysis (n = 73).

First Author et al. (publication year)^Ref^	Ancestry category	Regional population	Sample size Case/control	Genotyping method	Quality score (NOS)
Zeggini et al. (2005) (A)^52^	European	British/Irish	553/342	Pyrosequencing	8
Zeggini et al. (2005) (B)^52^	European	British/Irish	402/889	Pyrosequencing	8
Tripathi et al. (2013)^53^	European	Indo-European	190/210	PCR–RFLP	7
Lu et al. (2011)^54^	East Asian	Han Chinese	534/594	PCR–RFLP	6
Kommoju et al. (2014)^55^	South Asian	Hyderabad (Indian)	732/594	Sequenom Mass array	8
Muller et al. (2003)^56^	Native American	Pima Indian	657/328	PCR Sequencing	9
Oh et al. (2000)^57^	East Asian	Korean	58/111	PCR–RFLP	7
Memisoglu et al. (2003)^58^	European	Caucasian	387/771	Pyrosequencing	7
Mirzaei et al. (2009)^59^	Greater Middle Eastern	Iranian	156/156	PCR–RFLP	3
Mtiraoui et al. (2012) (A) (Lebanes)^60^	Greater Middle Eastern	Lebanese Arabs	751/918	Allelic discrimination method	7
Mtiraoui et al. (2012) (B) (Tunisian)^60^	Greater Middle Eastern	Tunisian Arabs	1,470/838	Allelic discrimination method	7
Zhu et al. (2017)^61^	East Asian	Eastern Chinese Han	497/782	SNP scan genotyping assay	7
Sokkar et al. (2009)^62^	Other	Tanta (Egypt)	24/30	PCR–RFLP	3
Wang et al. (2013)^63^	East Asian	Chinese Han	1,145/2001	TagMan	4
Bener et al. (2015)^64^	Greater Middle Eastern	Qatari	764/764	PCR followed by mutation analysis of the PCR product by real time PCR	6
Ye et al. (2014)^65^	East Asian	Chinese Han	198/255	PCR–RFLP	6
Bouassida et al. (2005)^66^	Greater Middle Eastern	Tunisian	242/246	PCR–RFLP	4
Simon et al. (2002)^67^	European	Caucasian origin from Catalonia	167/63	PCR–RFLP	5
Vergotine et al. (2014)^24^	Other admixed ancestry	Mixed ancestry population of South Africa	212/575	RT-PCR and TagMan genotyping assay Followed by direct sequencing	6
Moon et al. (2005)^68^	East Asian	Korean	677/281	PCR Sequencing	5
Pinterova et al. (2004)^20^	European	Czech	133/97	PCR–RFLP	2
Evans et al. (2001)^69^	European	Germany	219/429	PCR–RFLP	3
Badii et al. (2008)^70^	Greater Middle Eastern	Qatari	400/450	PCR followed by real time	5
Motavallian et al. (2013)^18^	Greater Middle Eastern	Iranian	100/100	PCR–RFLP	5
Phani et al. (2015)^71^	South Asian	Indian (Karnataka origin)	518/518	TETRA-ARMS	5
Sanghera et al. (2009)^72^	South Asian	Asian Indian Sikhs	527/518	TagMan	5
Majid et al. (2016)^23^	South Asian	Kashmiri	100/100	PCR–RFLP	3
Bouhaha et al. (2008)^73^	Greater Middle Eastern	Tunisian	84/261	Light typer system based on fluorescent	5
Sramkova et al. (2002)^74^	European	Czech	183/69	PCR–RFLP	6
Saleh et al. (2016)^75^	South Asian	Bangladeshi	25/28	PCR–RFLP	4
Paramasivam et al. (2016)^6^	South East Asian	Malaysian	120/121	PCR–RFLP	3
Pattanayak et al. (2014)^76^	South Asian	Indian (West Bengal)	200/200	PCR direct sequencing	5
Vimaleswaran et al. (2010)^77^	South Asian	South Indian	1,000/1,000	PCR- RFLP	4
Ringel et al. (1999)^78^	European	Germany	503/310	RFLP-PCR	5
Nemoto et al. (2002) (A)^79^	East Asian	Native Japanese	60/45	PCR-SSCP	4
Nemoto et al. (2002) (B)^79^	Asian unspecified	Japanese Americans	91/54	PCR-SSCP	4
Meshkani et al. (2007)^80^	Greater Middle Eastern	Iranian	284/412	PCR–RFLP	5
Hara et al. (2000)^17^	East Asian	Japanese	415/541	PCR–RFLP	3
Chistiakov et al. (2010)^22^	Other	Russian	588/597	TaqMan-based Real-Time PCR	6
Ghoussaini et al. (2005)^81^	European	French Caucasian	628/318	TaqMan AD Assay	4
Mato et al. (2016)^82^	Other	Cameroonian (Mixed)	60/60	PCR–RFLP	3
Malecki et al. (2003) ^83^	European	Polish	366/278	PCR- RFLP	5
Lara-Riegos et al. (2015) ^84^	Hispanic or Latin American	Maya	126/126	TagMan	5
Hegele et al. (2000) ^85^	Other	Canadian Oji-Cree	179/332	PCR Sequencing	2
Li et al. (2008) (A) (Uygur) ^86^	East Asian	Uygur	71/111	PCR–RFLP	4
Li et al. (2008) (B) (Kazak) ^86^	East Asian	Kazak	46/80	PCR–RFLP	4
Li et al. (2008) (C) (Han) ^86^	East Asian	Han	124/102	PCR–RFLP	4
Ho et al. (2012) (B) (Stage 1 + 2)^87^	East Asian	Hong Kong Chinese	1,461/600	Either the allele specific melting temperature shift assay at Roche Pharmaceuticals or the Sequenom i-PLEX gold assay	7
Hansen et al. (2005)^88^	European	Danish Caucasians	1,461/4,986	Chip-based matrix-assisted laser desorption/ionization time-of-flight mass spectrometry	6
Meirhaeghe et al. (2000)^89^	European	French	170/839	Allele-specific oligonucleotide hybridization	4
Costa et al. (2009)^90^	European	Italian	211/254	Gene-specific PCR and direct sequencing	4
Gragnoli et al. (2007)^91^	European	Italian	335/417	PCR followed by Sequencing	2
Erdogan et al. (2007)^2^	Greater Middle Eastern	Turkish	91/50	PCR–RFLP	6
Tavares et al. (2005)^92^	European	Brazilian Caucasians	207/170	PCR–RFLP	5
Raza et al. (2012)^42^	South Asian	North Indian	87/88	PCR–RFLP	4
Pei et al. (2013)^93^	East Asian	Chinese Han	197/212	MALDI-TOF mass spectrometry	5
Mohamed et al. (2007)^94^	Greater Middle Eastern	Tunisian	491/400	PCR–RFLP	6
Namvaran et al. (2011)^95^	Greater Middle Eastern	Iranian	101/128	RT-PCR with TagMan	5
Tariq et al. (2013)^96^	Greater Middle Eastern	Pakistani	373/200	PCR–RFLP	4
Doney et al. (2004)^21^	European	White from Scottish cities	1997/1,060	TagMan allelic discrimination assays	3
Mori et al. (2001)^16^	East Asian	Japanese	2,201/1,212	PCR–RFLP	3
Clement et al. (2000)^97^	European	French (Caucasian)	402/295	PCR–RFLP	3
Avzaletdinova et al. (2016)^98^	Other	Republic of Bashkortostan	294/326	Real-time PCR using the TaqMan	3
Kao et al. (2003)^99^	African American or Afro-Caribbean	African-American	436/1,005	PCR	3
Wang et al. (2009)^100^	East Asian	Chinese Han	395/391	Minisequencing	5
Horiki et al. (2004) ^101^	East Asian	Japanese	227/278	PCR–RFLP	4
Douglas et al. (2001) ^19^	European	Finnish	522/413	MALDI-TOF mass spectrometry	3
Lv et al. (2017) ^102^	East Asian	Chinese Han	647/650	TagMan fluorescence probe	4
Mancini et al. (1999) ^103^	European	Italian-Caucasian	131/312	PCR–RFLP	3
Radha et al. (2006) (South Asian living in Chennai) ^104^	South Asian	South Asian	799/820	PCR–RFLP	6
Radha et al. (2006) (South Asian living in Dallas) ^104^	South Asian	South Asian	81/616	PCR–RFLP	6
Radha et al. (2006) (Caucasian living in Dallas) ^104^	European	Caucasian	123/334	PCR–RFLP	6
Martínez‐Gómez et al. (2011) (Combined) ^105^	Hispanic or Latin American	Mexican	719/746	Real-Time PCR by TagMan	8

*BMI* body mass index, *PCR* polymerase chain reaction, *PCR–RFLP* polymerase chain reaction-restriction fragments length polymorphism, *NOS* Newcastle–Ottawa scale, *TETRA-ARMS tetra*-primer amplification refractory mutation system, *RT-PCR* reverse transcription polymerase chain reaction, *AD* allelic discrimination, *MALDI-TOF* matrix-assisted laser desorption/ionization time-of-flight, *Ref* reference.

The NOS score of eligible articles ranged from two to eight stars. 11 of included studies were evaluated to be high quality, 33 were low quality, and 28 studies were considerate as moderate quality.

There were 21 studies of Europeans, 17 studies of East Asians, 10 South Asians, 13 Greater Middle Eastern, five other, and two Hispanic or Latin American. Other ancestry groups such as South East Asian, Asian unspecified, other admixed ancestry, Native American, and African American or Afro-Caribbean included only one study.

Different genotyping methods consist of TaqMan, *tetra-*primer amplification refractory mutation system *(*TETRA-ARMS), restriction fragment length polymorphism (RFLP-PCR), mass spectrometry, direct sequencing, real-time PCR, and etc. which was listed in Table 1.

The genotype frequency of the control group met to HWE in the included studies except for five case–control studies and one study that not reported the p-value of HWE.

### The results of meta-analysis

Combining the results of the primary studies showed a significant association between the Pro12Ala polymorphism and T2DM risk under REM in seven genetic models including Allelic (OR: 0.82, 95% CI: 0.76–0.89, *P* < 0.01) with high between-study heterogeneity (I^2^ = 71%), homozygous, heterozygous, additive, dominant, recessive, and co-dominant genetic models. Further details on the genetic coding of the variant are provided in Table 2, and the forest plots are shown in Supplementary Fig. S1 online.

**Table 2:** The meta-analysis results of association between Pro12Ala variant and T2DM risk.

Genetic model	No of studies	Number		Test of association OR (95% CI)	Statistical model	Test of heterogeneity		Test of publication bias
		Case	Control			I^2^ (%)	P_H_	P _Egger_
Allele model: G vs. C	73	62,250	69,613	0.82 (0.76;0.89)	REM	71	< 0.01	< .0001
Homozygote model: GG vs. CC	62	20,666	23,618	0.68 (0.53;0.88)	REM	49	< 0.01	0.7340
Heterozygote model: CG vs. CC	62	24,165	27,505	0.84 (0.77;0.93)	REM	64	< 0.01	0.4790
Additive model: GG vs. CG	62	4,207	5,706	0.77 (0.62;0.97)	REM	29	0.03	0.8527
Dominant model: CG + GG vs. CC	62	24,491	28,792	0.84 (0.77;0.92)	REM	63	< 0.01	0.1695
Recessive model: GG vs. CC + CG	62	24,491	28,792	0.71 (0.56;0.90)	REM	45	< 0.01	0.7372
Codominant model: CG vs. CC + GG	62	24,491	28,792	0.87 (0.81;0.95)	REM	52	< 0.01	0.7074

*OR* odds ratio, *CI* confidence interval, *REM* random effect model, *FEM* fixed-effects model, *I*^*2*^ I-squared metric of the heterogeneity, *P*_*H*_ P value of heterogeneity, *Q test*, P_Egger_ P value of Egger linear regression test. I^2^ value of 25%, 50%, and 75% were nominally regarded as low, moderate, and high estimates, respectively.

The OR analysis results designed for the allele (5 studies), additive (4 studies), and dominant (4 studies) genetic models that were analyzed according to just OR (totally 13 studies) were consistent with the main results of the meta-analysis (Supplementary file 2 online).

### Subgroup analysis

Subgroup analysis by ancestry categories revealed a significant association between *PPARG* rs1801282 polymorphisms and T2DM in the European populations under allelic (OR: 0.82, 95% CI: 0.73–0.91), homozygous (OR: 0.74, 95% CI: 0.59–0.92), heterozygous (OR: 0.88, 95% CI: 0.79–0.98), additive (OR: 0.76, 95% CI: 0.58–0.98), dominant (OR: 0.86, 95% CI: 0.77–0.96), recessive (OR: 0.75, 95% CI: 0.61–0.93), and co-dominant (OR: 0.88, 95% CI: 0.82–0.95) genetic models (Table 3).

**Table 3:** Summary of subgroup analysis according to ancestry categories, BMI and age of participants, and publication year.

		Allele model (G vs. C)					Homozygous model (GG vs. CC)				Heterozygous model (CG vs. CC)			
	٭N	Cases/Controls	OR (95% CI)	P_H_	I^2^	٭٭N	Cases/Controls	OR (95% CI)	P_H_	I^2^	Cases/Controls	OR (95% CI)	P_H_	I^2^
Total	73	62,250/69,613	**0.82 (0.76–0.89)**	< 0.01	71%	62	20,666/23,618	**0.68 (0.53–0.88)**	< 0.01	49%	24,165/27,505	**0.84 (0.77–0.93)**	< 0.01	64%
**Ancestry categories**														
European	21	18,580/25,712	**0.82 (0.73–0.91)**	< 0.01	60%	21	6,847/9,395	**0.74 (0.59–0.92)**	0.15	27%	8,373/11,818	**0.88 (0.79–0.98)**	0.05	38%
East Asian	17	17,906/16,491	**0.80 (0.63–1.00)**	< 0.01	78%	9	6,336/6,192	2.09 (1.39–3.14)	0.58	0%	7,007/6,266	0.76 (0.56–1.02)	< 0.01	82%
South Asian	10	8,138/8,964	**0.82 (0.71–0.95)**	0.03	51%	20	2,604/2,430	**056 (0.36–0.86)**	0.10	42%	3,133/2,980	**0.88 (0.78–1.00)**	0.05	50%
Greater Middle Eastern	13	10,614/9,846	0.89 (0.70–1.14)	< 0.01	77%	8	2,670/2,745	0.82 (0.36–1.87)	0.02	56%	3,036/3,090	0.87 (0.69–1.10)	0.03	50%
Other	5	2,290/2,690	0.74 (0.45–1.21)	< 0.01	85%	3	862/979	0.63 (0.18–2.19)	0.01	72%	1,112/1,292	0.84 (0.58 -1.22)	0.04	64%
**BMI**														
< 25 kg/m^2^	17	20,812/19,248	**0.67 (0.52–0.87)**	< 0.01	90%	13	6,570/6,296	0.78 (0.40–1.52)	< 0.01	59%	7,310/6,404	0.82 (0.62–1.09)	< 0.01	84%
≥ 25 kg/m^2^	30	22,566/31,229	0.88 (0.77–1.01)	< 0.01	77%	24	6,104/9,752	**0.59 (0.40–0.88)**	< 0.01	47%	7,443/11,988	0.96 (0.85–1.10)	< 0.01	50%
**Age**														
< 50	19	17,040/27,888	**0.79 (0.69–0.90)**	< 0.01	69%	13	4,356/8,569	**0.73 (0.54–0.98)**	0.07	40%	5,131/9,714	0.81 (0.64–1.04)	< 0.01	80%
≥ 50	30	2,798/23,571	**0.78 (0.64–0.94)**	< 0.01	87%	28	9,010/7,886	0.62 (0.38–1.03)	< 0.01	63%	10,415/9,161	0.94 (0.81–1.09)	< 0.01	60%
**Publication year**														
< 2005	21	19,008/17,684	**0.78 (0.69–0.90)**	< 0.01	57%	18	7,239/6,508	0.79 (0.61–1.03)	0.47	0%	8,328/7,736	**0.82 (0.70–0.95)**	< 0.01	51%
≥ 2005	52	43,716/51,355	**0.79 (0.70–0.89)**	< 0.01	82%	44	13,427/17,100	**0.65 (0.46–0.92)**	< 0.01	58%	15,837/19,769	**0.86 (0.76–0.96)**	< 0.01	67%

	Additive model (GG vs. CG)				Dominant model (GG + CG vs. CC)				Recessive model (CG vs. CC + GG)					
	Cases/Controls	OR (95% CI)	P	I^2^	Cases/Controls	OR (95% CI)	P	I^2^	Cases/Controls	OR (95% CI)	P	I^2^		
Total	4,207/5,706	**0.77 (0.62–0.97)**	0.03	29%	24,491/28,792	**0.84 (0.77–0.92)**	< 0.01	63%	24,491/28,792	**0.71 (0.59–0.90)**	< 0.01	45%		
**Ancestry categories**														
European	1793/2,924	**0.76 (0.58–0.98)**	0.59	0%	8,478/12,046	**0.86 (0.77–0.96)**	0.01	48%	8,478/12,046	**0.75 (0.61–0.93)**	0.22	20%		
East Asian	811/952	1.74 (1.15–2.64)	0.91	0%	7,077/7,105	0.82 (0.65–1.05)	< 0.01	75%	7,077/7,105	1.91 (1.28–2.86)	0.71	0%		
South Asian	641/682	**0.54 (0.35–0.84)**	0.07	46%	3,189/3,046	0.85 (0.68–1.06)	0.01	61%	3,189/3,046	**0.60 (0.40–0.90)**	0.12	39		
Greater Middle Eastern	465/499	0.90 (040–2.00)	0.02	55%	3,086/3,167	0.84 (0.64–1.08)	< 0.01	60%	3,086/3,167	0.85 (0.37–1.94)	< 0.01	61		
Other	316/419	0.90 (0.55–1.48)	0.21	33%	1,145/1,345	0.76 (0.47–1.23)	< 0.01	80%	1,145/1,345	0.71 (0.23–2.24)	0.03	68		
**BMI**														
< 25 kg/m^2^	946/1,068	1.00 (0.71–1.40)	0.03	46%	7,413/7,284	0.87 (0.69–1.10)	< 0.01	79%	7,413/7,284	0.82 (0.45–1.48)	0.02	52%		
≥ 25 kg/m^2^	1596/2,840	**0.68 (0.54–0.86)**	0.12	27%	7,572/12,290	0.93 (0.80–1.07)	< 0.01	64%	7,572/12,290	**0.61 (0.42–0.89)**	< 0.01	46%		
**Age**														
< 50	935/2,321	0.77 (0.57–1.04)	0.18	26%	5,211/10,702	0.84 (0.70–1.02)	< 0.01	67%	5,211/10,702	**0.75 (0.56–1.00)**	0.13	32%		
≥ 50	1712/1679	**0.67 (0.45–1.00)**	0.01	44%	10,569/9,363	0.90 (0.77–1.06)	< 0.01	70%	10,569/9,363	0.63 (0.40–1.01)	< 0.01	61%		
**Publication year**														
< 2005	190/1,451	0.81 (0.58–1.15)	0.91	0%	8,400/7,825	**0.81 (0.70–0.94)**	< 0.01	55%	8,400/7,825	0.80 (0.62–1.04)	0.59	0%		
≥ 2005	2,917/4,255	0.75 (0.56–1.01)	< 0.01	43%	16,091/20,957	**0.85 (0.77–0.95)**	< 0.01	65%	16,091/20,957	**0.68 (0.49–0.94)**	< 0.01	55%		

	Co-dominant model (CG vs. CC + GG)													
	Cases/controls	OR (95% CI)	P	I^2^										
Total	**24,491/28,972**	**0.87 (0.81–0.95)**	** < 0.01**	**52%**										
**Ancestry categories**														
European	8,478/12,046	**0.88 (0.82–0.95)**	0.08	35%										
East Asian	7,077/7,105	**0.80 (0.65–0.98)**	< 0.01	64%										
South Asian	3,189/3,046	**0.88 (0.78–1.00)**	0.12	38%										
Greater Middle Eastern	3,086/3,167	0.98 (0.84–1.14)	< 0.01	65%										
Other	1,145/1,345	**0.83 (0.69–1.00)**	0.12	48%										
**BMI**														
< 25 kg/m^2^	7,413/7,284	0.84 (0.70–1.02)	< 0.01	64%										
≥ 25 kg/m^2^	7,572/12,290	1.02 (0.89–1.16)	< 0.01	53%										
**Age**														
< 50	5,211/10,702	**0.84 (0.73–0.98)**	0.04	45%										
≥ 50	10,569/9,363	0.98 (0.85–1.13)	< 0.01	60%										
**Publication year**														
< 2005	8,400/7,825	**0.82 (0.71–0.95)**	< 0.01	49%										
≥ 2005	16,091/20,957	**0.89 (0.81–0.98)**	< 0.01	52%										

Bold values indicate that the values have statistical significant.

*BMI* body mass index, *OR* odds ratio, *CI* confidence interval, *I*^*2*^ I-squared metric of the heterogeneity, *P*_*H*_ P value of heterogeneity**.**

٭Number of studies in allele model.

٭٭Number of studies in genetic models.

A significant association was also detected in the East Asian populations under allelic (OR: 0.80, 95% CI: 0.63–1.00) and co-dominant (OR: 0.80, 95% CI: 0.65–0.98) genetic models (Table 3).

There was a significant association in the South Asian populations under allelic (OR: 0.82, 95% CI: 0.71–0.95), homozygous (OR: 0.56, 95% CI: 0.36–0.86), heterozygous (OR: 0.88, 95% CI: 0.78–1.00), additive (OR: 0.54, 95% CI: 0.35–0.84), recessive (OR: 0.60, 95% CI: 0.40–0.90), and co-dominant (OR: 0.88, 95% CI: 0.78–1.00) genetic models (Table 3).

There was not a significant association detected under all genetic models in the Greater Middle Eastern population.

In addition, we only found significant association in the category named “other” under co-dominant (OR: 0.83, 95% CI: 0.69–1.00) genetic model (Table 3).

Other ancestry categories including South East Asian, Asian unspecified, African American or Afro-Caribbean, Hispanic or Latin American, Native American, and Other admixed ancestry were not reliable to report due to the low number of publications.

**Figure 2:**
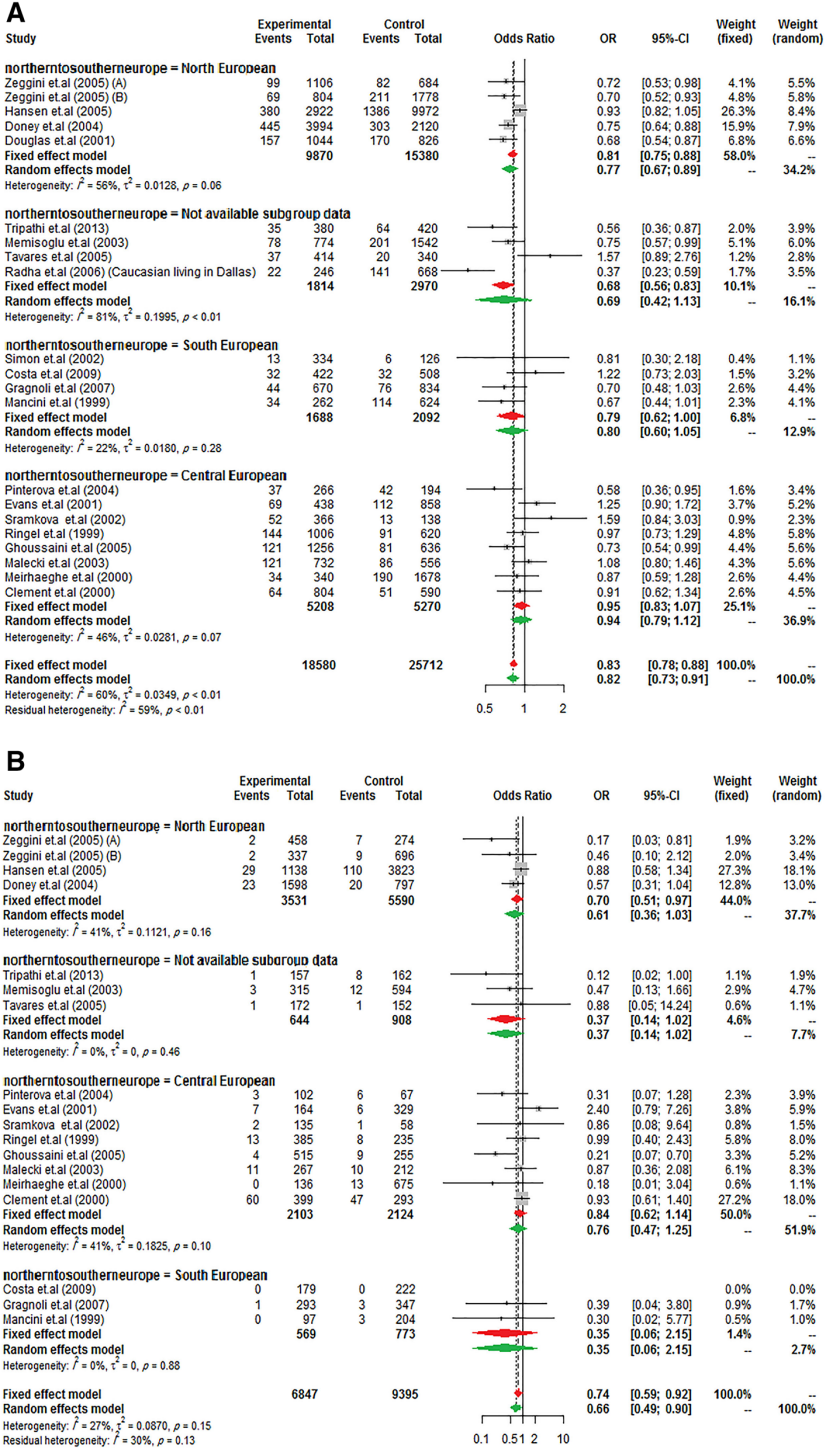

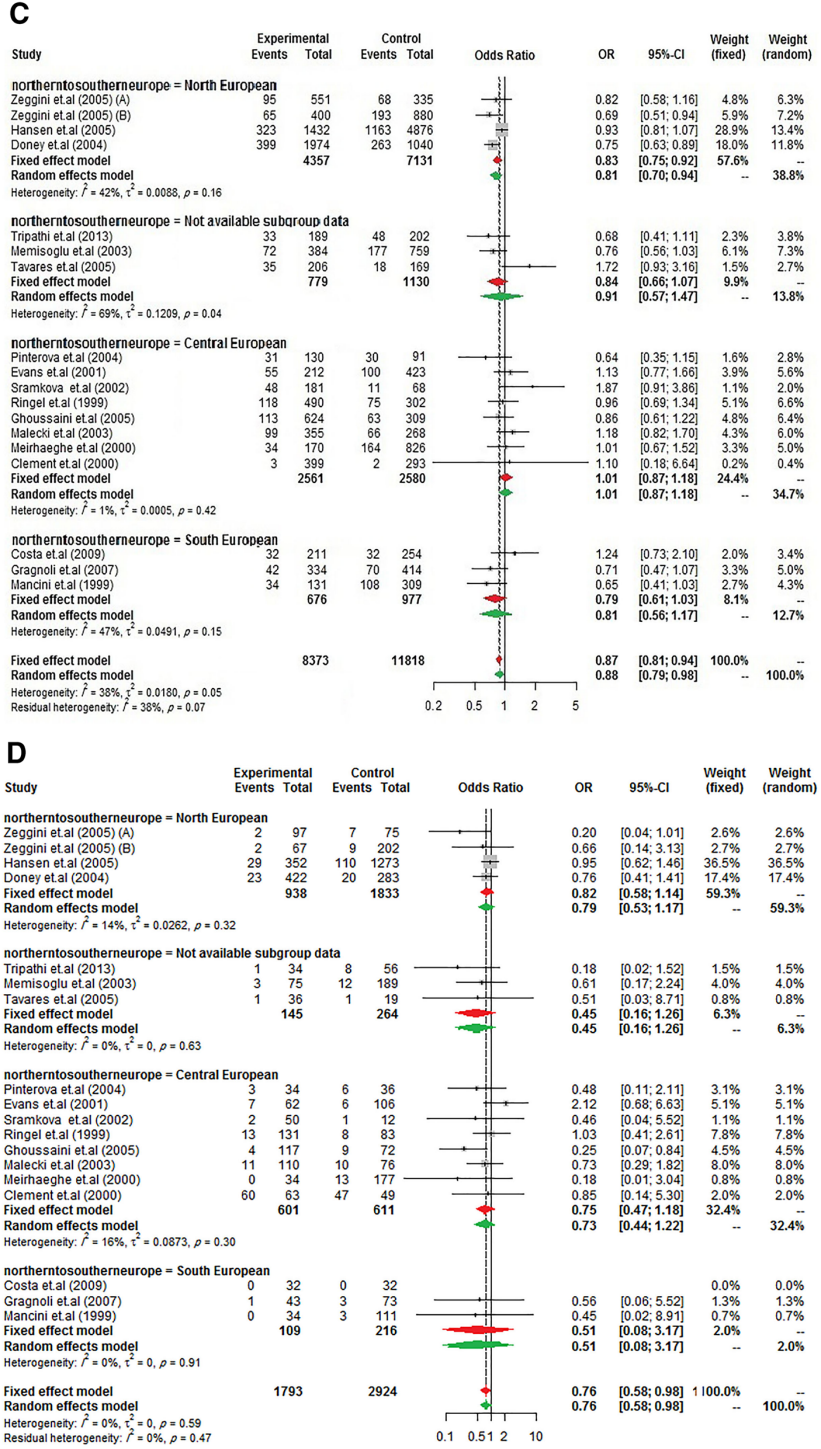

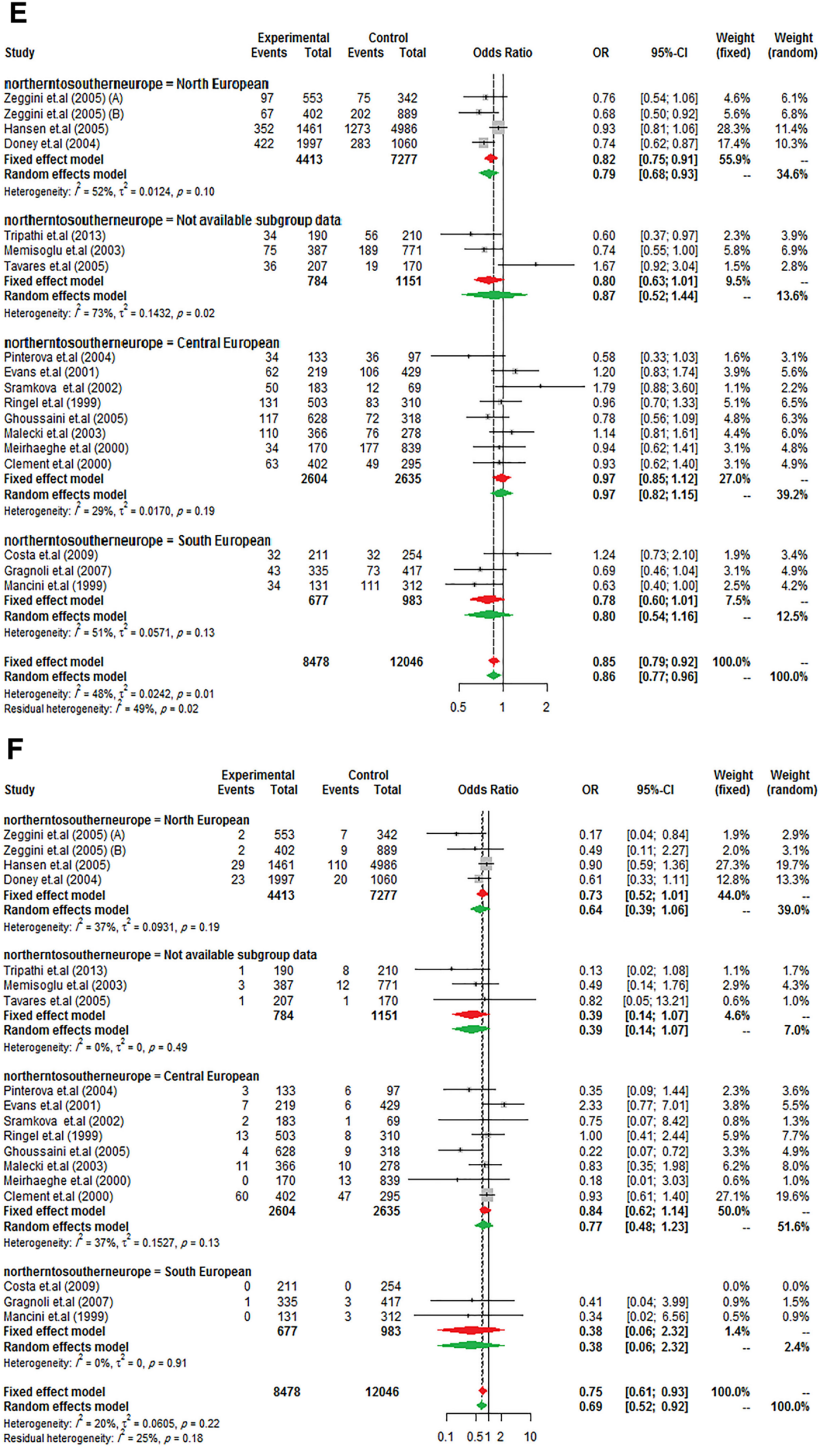

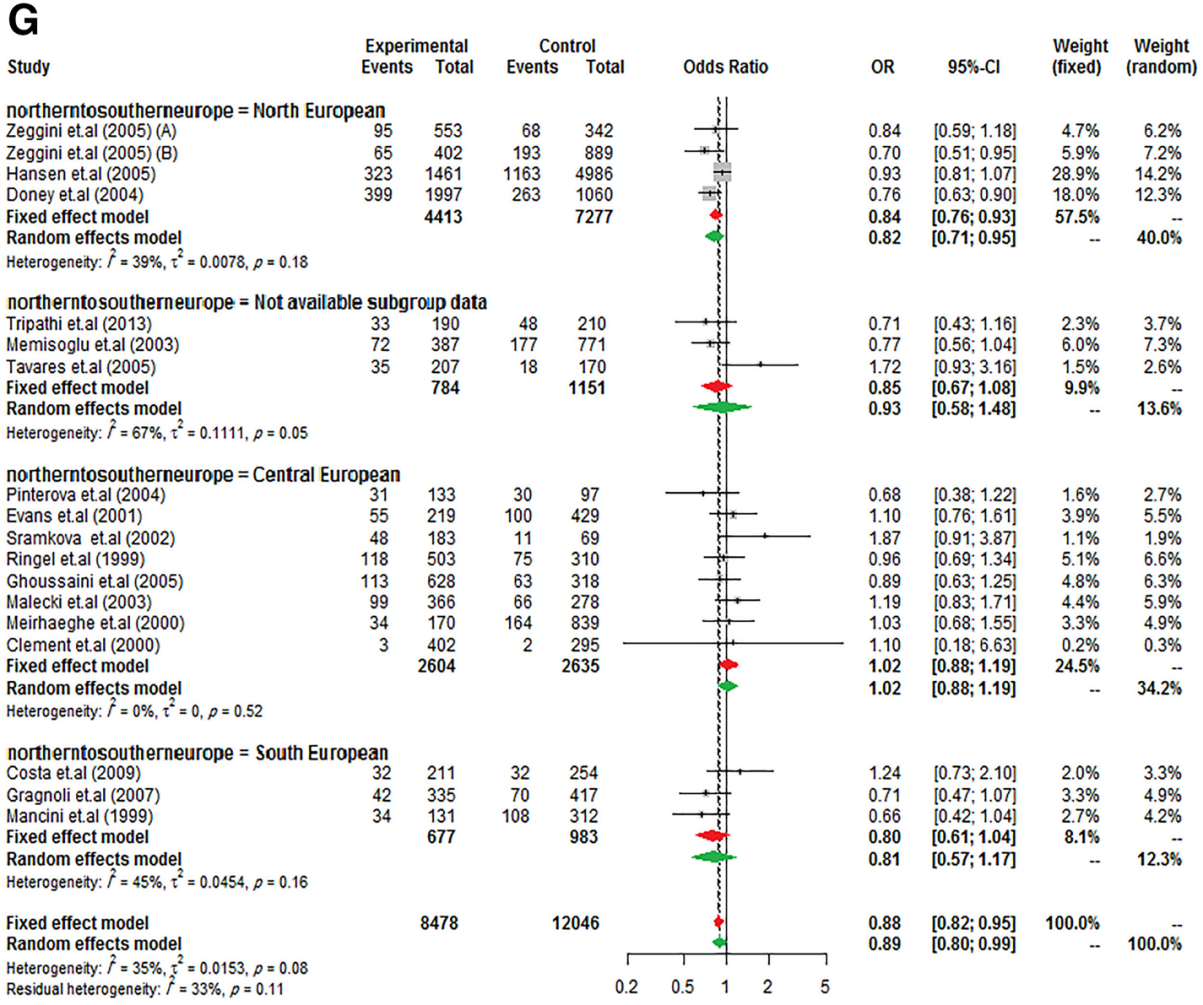
Risk of T2DM according to *PPARG* Ala12 variant from North to South European ancestry. (**A**) allele model, (**B**) homozygote model, (**C**) heterozygote model, (**D**) additive model, (**E**) dominant model, (**F**) recessive model, and (**G**) co-dominant model.

Same to the results of the study by Ludovico et al. ^27^, it was observed a significant between-study heterogeneity among Europeans, whereas it was not in other ancestry categories. So, to further subgroup analysis, data from Europe was stratified to "North European" (Scottish, British, and Finnish), "Central European" (Poles, Germans, French, Czechs), "South European" (Italians and Spaniards), and "not available subgroup data" (such as Caucasian) to confirm the previously reported findings. As presents in Fig. 2, the reduction of T2DM risk in G allele carriers from Northern and Southern Europe was almost equal (23% and 21%, respectively) but did not influence in Central Europe. However, it should be noted that the negative result may have been due to the low sample size in Central Europe studies.

Furthermore, in subgroup analysis, participants with mean BMI ≥ 25 kg/m^2^ indicated a strong association with T2DM risk for homozygous (OR for participants with mean BMI ≥ 25 vs. BMI < 25 = 0.59 vs. 0.78), additive (OR for participants with mean BMI ≥ 25 vs. BMI < 25 = 0.68 vs. 1.00), and recessive (OR for participants with mean BMI ≥ 25 vs. BMI < 25 = 0.61 vs. 0.82) genetic models (see Supplementary Fig. S2 online).

Nevertheless, no significant association was found among age and year of publication (see Supplementary Fig. S3, Fig. S4 online).

### Sensitivity analysis

Although, the combined results remained stable after removing single studies in the allele, homozygote, heterozygote, dominant, recessive, and co-dominant models, but the pooled OR was go away from significantly after omitting the study by Motavallian et al. (2013)^18^ and Raza et al. (2012)^42^ in the additive model indicating that the results of these studies had the highest influence on the pooled estimate (see Supplementary Fig. S5 online).

Furthermore, we excluded those HWE-violating studies for sensitivity analyses. However, the pooled ORs in overall was not statistically altered, indicating that the results were stable (see Supplementary Fig. S6 online).

The evidence of sensitivity analysis suggested that removing poor-quality studies could not influence the combined results (see Supplementary Fig. S7 online).

### Heterogeneity and publication bias

Heterogeneity was detected under the allele model (G vs. C), heterozygote model (CG vs. CC), dominant model (GG + CG vs. CC), and co-dominant model (CG vs. CC + GG) genetic models.

No evidence of asymmetry was observed among the primary studies by Begg funnel plots in any comparisons (see Supplementary Fig. S8 online). The Egger's regression test indicated that there was no evidence of potential statistical publication bias in either of genetic models except in allele model and the results were constant and sturdy (Table 2).

## Discussion

Diabetes is one of the major driver of morbidity and mortality worldwide and in spite of introducing approximately 100 identified susceptibility loci with robust interaction signals with T2DM but most of them show little value in clinical practice^43^.

It seems that the *PPAR-γ* plays an important role in the pathological process of diabetes. The functional role of *PPAR-γ* has been well described, and its variations in association with TDM and obesity have been extensively investigated in different ethnicities^44^.

Many GWASs have used SNPs to investigate the development risk of T2DM^12,45,46^.

Pro12Ala (rs1801282) is considered to be the most analyzed common variants in the *PPARG* gene which decreases the receptor binding affinity to the responsive elements and consequently inducing a reduction in transcriptional activity both with and without PPAR-γ agonists using effect on receptor structure which eventually leads to insulin sensitivity and abnormalities of adipose tissue formation^47^. The more common (C) and rare (G) alleles of rs1801282 encode the 'Pro' and 'Ala' amino acids, respectively. According to the previous GWAS, the Pro allele of this variant was reported to increase the risk of T2DM. But, the Ala allele has a protective effect on T2DM development^12,46,48^.

The result of the present systematic review and meta-analysis consists of 62,250 cases and 69,613 controls from 73 studies in order to achieve substantial evidence of any association between *PPAR-γ* rs1801282 and T2DM risk. The findings of this meta-analysis showed that the G allele of Pro12Ala polymorphism could cause approximately an 18% reduction in the probability of developing T2DM. The reduction of the T2DM risk was also detected vary across different ethnicities; European (18%), East Asian (20%), and South Asian (18%) while no association was founded in the Greater Middle Eastern population.

As is obvious, the differences in the reduction of T2DM risk between those of European, South Asian, and East Asian ancestries are not really very different in the present study whereas in a study by Ludovico et al.^27^ the reduced risk in the Asian population more than European (35% vs. 14%) was reported. Consistent with the previous report, the reduced T2DM risk was stronger in North European populations in stratified Europe from Northern to Southern gradient^27^.

The contradictory results from the different ethnic populations appear interesting that it can be partially attributed to the small sample size and the fact that different genetic backgrounds and various environmental factors might be lead to conflicting results from the same polymorphism among primary studies with different ethnicities^49^. It indicates that the stratification of the studies based on different ethnicities is very important in the present meta-analysis.

The present result was different from previous studies exclusively in the Chinese Han populations^26,29^ which indicated the Pro12Ala variant of *PPARG* is not associated with T2D risk.

However, it was consistent with recent meta-analysis research results with 20,702 cases and 36,227 controls from 14 studies^30^ which showing evidence of Pro12Ala as a susceptibility variant for the lowering development of T2DM.

It should be noticed that the results of our study could be closer to reality due to the number of cases, controls, and studies of different ancestries. Furthermore, a study by Huguenin et al. showed a significant effect of the Ala allele on reduction of T2DM risk in Caucasians^28^. Also, a meta-analysis by Gouda in 2010 observed that the *PPAR-γ* Pro12Ala variant is positively associated with a reduction of T2DM risk^50^.

The analysis of subjects harboring polymorphisms within *PPAR-γ* has made an important contribution in providing convincing genetic evidence of a role in glucose homeostasis, lipid metabolism, and determination of fat mass. Such studies also provided data for the underlying mechanisms of insulin sensitivity, *PPAR-γ* action and, T2DM risk. However, neither environmental triggers nor genetics alone can explain T2D pathogenesis because of its multifactorial nature. Hence, more functional studies and large population-based validation surveys are needed to perform. To the best of our knowledge, this is one of the most comprehensive meta-analysis of the association of *PPAR-γ* rs1801282 (Pro12Ala) polymorphism and T2DM risk.

### Limitations

Despite our promising findings, multiple limitations should also be addressed. Firstly, T2DM is a complex disorder and we only discussed individual genetic variant without having to consider the interaction with other genetic variants or environmental variables (lifestyle, smoking, etc.).

Secondly, owing to the restriction of the accessibility of original research information, the study did not consider other appropriate variables such as gender, age, and genotype frequency data as the genotype frequency data was not available in some articles (11 from 73) and only the allele model was evaluated in order to assess the association among the overall population**.** Therefore, a more precise association with sufficient data should be explored. These results should be interpreted with caution until further sequencing approaches verification and greater meta-analysis is required.

Thirdly, significant publication bias was observed in some T2DM comparisons including the allele model. This may be due to the fact that the ethnicity of the populations in the early studies is mostly European or Asian, or that there is a greater number of low or medium quality studies rather than the high-quality ones. And also, significant heterogeneity was detected in the primary study results, indicating that the inconsistent results of the included studies could not be fully explained by differences in ethnic background, BMI, age, and other unmeasured variables of participants that may also partially attribute heterogeneity to the inter-study.

Fourthly, there are some gaps about particular ancestry groups including; Aboriginal Australian, African unspecified, Asian unspecified, Central Asian, Oceanian, and Sub-Saharan African that should be addressed.

Finally, obesity is also a significant intermediate factor in the rise of T2DM and having BMI information would be important and useful in the association analysis. But the definitions of obesity were not the same or accessible in our included studies. Therefore, in our subgroup analysis with BMI, the mean BMI of populations was used which does not indicate the exact BMI individual level of the study. So, this may be causing the contradictory result of this stratification.

Moreover, despite these limitations, our comprehensive research can still make a valid conclusion.

## Conclusion

Genomic association studies help in disease predispositions by using genomic variants which have been discovered by GWASs^10^. The introduced genetic variants can be used to detect high-risk individuals for certain diseases. Thereby personalized medicine goals for improving patient outcomes will be achieved through such studies. A genetic variant that is associated with disease in one ethnic group but not in another may indicate ethnic differences in risk disease predisposition. So the result of genetic association studies represents only the tip of the iceberg and meta-analysis study shows great benefit for the personalized medicine approach^10^. The *PPARG* Pro12Ala variant in current meta‐analysis indicated enough evidence for the presence of a significant association of individuals carrying the *PPARG* Ala12 variant with a reduced risk of T2DM. Additionally, the results of analysis under diverse ancestries confirm the importance of SNPs association studies in different ethnicities. But this effect is not very different among European compared to other ancestries. And among Europeans, existence stronger in North European, and barely significant in South European, and not being in South European. The genetic architecture of diabetes, including polygenicity and most risk variants, has been discussed in previous studies with important implications for precision medicine^51^. But several obstacles complicate the translation of novel loci and variants into the clinical decision practice, overcoming these will lead to the development of new drugs to treat T2DM.

It appears that the lack of efficacy in anti-diabetic drugs is returned to the preclinical models in clinical trials. Human genomic advancement provides a better condition for proper assessment of drug development efficacy in pharmaceutical R&D through combining a targeted pathway or genetic alteration to a desirable phenotype (T2DM).

Decision-making through precision medicine needs therapeutic approaches which are obtained by the genetic association study of the common variants/loci.

The identification of the right drugs that are most effective and safe for each patient and reducing the global economic impact will be possible when the genetic information of the diabetic patients will provide a valuable resource to predict T2DM progression. Genetic studies are one of the most important approach in order to predict and prevent T2DM in the near future. It is hoped, therefore, that the decades ahead will elucidate the extent to which the inherited variation and its interaction with the environmental factors help clinicians’ diagnostics.

## Supplementary information

Supplementary Information 1.

Supplementary Information 2.

Supplementary Information 3.

Supplementary Information 4.

Supplementary Information 5.

Supplementary Information 6.

Supplementary Information 7.

Supplementary Information 8.

Supplementary Information 9.

Supplementary Information 10.

Supplementary Information 11.

Supplementary Information 12.

## Data Availability

Data sharing is not applicable to this type of article.
