# Supplementary material for: PPARG (Pro12Ala) genetic variant and risk of T2DM: a systematic review and meta-analysis
Source: Sci Rep. 2020 Jul 29;10:12764. doi: 10.1038/s41598-020-69363-7 (PMC7391673; doi:10.1038/s41598-020-69363-7)
Supplement: Supplementary file 1 — Supplementary Information 1. [file 41598_2020_69363_MOESM1_ESM.pdf]

PPARG (Pro12Ala) genetic variant and risk of T2DM: a systematic review and meta-analysis

Negar Sarhangi [1], Farshad Sharifi [2], Leila Hashemian [3], Maryam Hassani Doabsari [3], Katayoun Heshmatzad [3], Marzieh Rahbaran [3], Seyed Hamid Jamaldini [3], Hamid Reza Aghaei Meybodi [1, 4], Mandana Hasanzad [1,3,*]

[1] Personalized Medicine Research Center, Endocrinology and Metabolism Clinical Sciences Institute, Tehran University of Medical Sciences, Tehran 1411413137, Iran

[2] Elderly Health Research Center, Endocrinology and Metabolism Population Sciences Institute, Tehran University of Medical Sciences, Tehran 1411413137, Iran

[3] Medical Genomics Research Center, Tehran Medical Sciences, Islamic Azad University, Tehran 1916893813, Iran

[4] Endocrinology and Metabolism Research Center, Endocrinology and Metabolism Clinical Sciences Institute, Tehran University of Medical Sciences, Tehran 1411413137, Iran

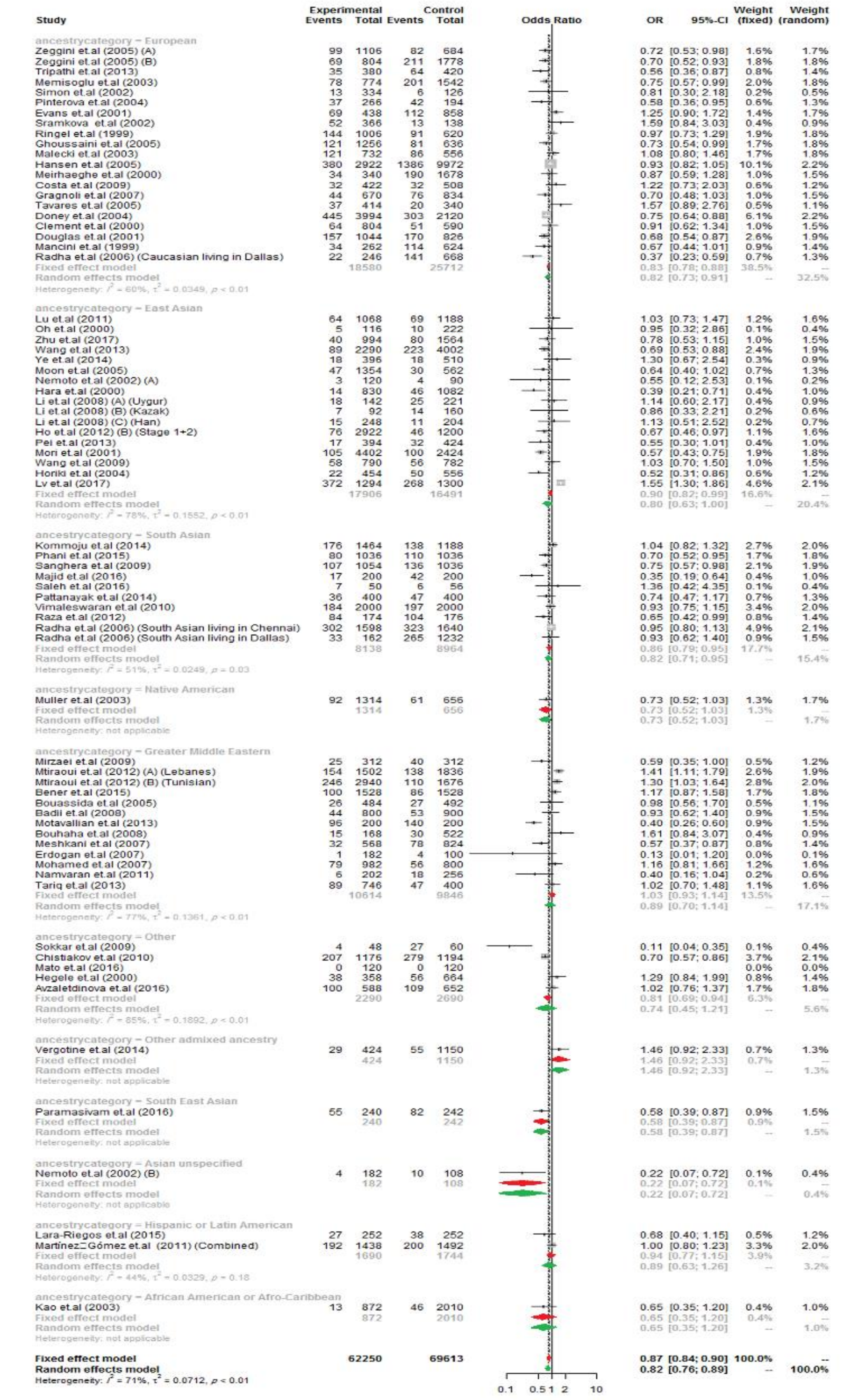
Study
Experimental Events Total
Control Events Total
Odds Ratio
OR 95%-CI
Weight (fixed)
Weight (random)
ancestrycategory = European
Zeggini et.al (2005) (A) 99 1106 82 684 0.72 [0.53; 0.98] 1.6% 1.7%
Zeggini et.al (2005) (B) 69 804 211 1778 0.70 [0.52; 0.93] 1.8% 1.8%
Tripathi et.al (2013) 35 380 64 420 0.56 [0.36; 0.87] 0.8% 1.4%
Memisoglu et.al (2003) 78 774 201 1542 0.75 [0.57; 0.99] 2.0% 1.8%
Simon et.al (2002) 13 334 6 126 0.81 [0.30; 2.18] 0.2% 0.5%
Pinterova et.al (2004) 37 266 42 194 0.58 [0.36; 0.95] 0.6% 1.3%
Evans et.al (2001) 69 438 112 858 1.25 [0.90; 1.72] 1.4% 1.7%
Sramkova et.al (2002) 52 366 13 138 1.59 [0.84; 3.03] 0.4% 0.9%
Ringel et.al (1999) 144 1006 91 620 0.97 [0.73; 1.29] 1.9% 1.8%
Ghoussaini et.al (2005) 121 1256 81 636 0.73 [0.54; 0.99] 1.7% 1.8%
Malecki et.al (2003) 121 732 86 556 1.08 [0.80; 1.46] 1.7% 1.8%
Hansen et.al (2005) 380 2922 1386 9972 0.93 [0.82; 1.05] 10.1% 2.2%
Meirhaeghe et.al (2000) 34 340 190 1678 0.87 [0.59; 1.28] 1.0% 1.5%
Costa et.al (2009) 32 422 32 508 1.22 [0.73; 2.03] 0.6% 1.2%
Gragnoli et.al (2007) 44 670 76 834 0.70 [0.48; 1.03] 1.0% 1.5%
Tavares et.al (2005) 37 414 20 340 1.57 [0.89; 2.76] 0.5% 1.1%
Doney et.al (2004) 445 3994 303 2120 0.75 [0.64; 0.88] 6.1% 2.2%
Clement et.al (2000) 64 804 51 590 0.91 [0.62; 1.34] 1.0% 1.5%
Douglas et.al (2001) 157 1044 170 826 0.68 [0.54; 0.87] 2.6% 1.9%
Mancini et.al (1999) 34 262 114 624 0.67 [0.44; 1.01] 0.9% 1.4%
Radha et.al (2006) (Caucasian living in Dallas) 22 246 141 668 0.37 [0.23; 0.59] 0.7% 1.3%
Fixed effect model 18580 25712 0.83 [0.78; 0.88] 38.5% --
Random effects model 0.82 [0.73; 0.91] -- 32.5%
Heterogeneity: I^2 = 60%, τ^2 = 0.0349, p < 0.01
ancestrycategory = East Asian
Lu et.al (2011) 64 1068 69 1188 1.03 [0.73; 1.47] 1.2% 1.6%
Oh et.al (2000) 5 116 10 222 0.95 [0.32; 2.86] 0.1% 0.4%
Zhu et.al (2017) 40 994 80 1564 0.78 [0.53; 1.15] 1.0% 1.5%
Wang et.al (2013) 89 2290 223 4002 0.69 [0.53; 0.88] 2.4% 1.9%
Ye et.al (2014) 18 396 18 510 1.30 [0.67; 2.54] 0.3% 0.9%
Moon et.al (2005) 47 1354 30 562 0.64 [0.40; 1.02] 0.7% 1.3%
Nemoto et.al (2002) (A) 3 120 4 90 0.55 [0.12; 2.53] 0.1% 0.2%
Hara et.al (2000) 14 830 46 1082 0.39 [0.21; 0.71] 0.4% 1.0%
Li et.al (2008) (A) (Uygur) 18 142 25 221 1.14 [0.60; 2.17] 0.4% 0.9%
Li et.al (2008) (B) (Kazak) 7 92 14 160 0.86 [0.33; 2.21] 0.2% 0.6%
Li et.al (2008) (C) (Han) 15 248 11 204 1.13 [0.51; 2.52] 0.2% 0.7%
Ho et.al (2012) (B) (Stage 1+2) 76 2922 46 1200 0.67 [0.46; 0.97] 1.1% 1.6%
Pei et.al (2013) 17 394 32 424 0.55 [0.30; 1.01] 0.4% 1.0%
Mori et.al (2001) 105 4402 100 2424 0.57 [0.43; 0.75] 1.9% 1.8%
Wang et.al (2009) 58 790 56 782 1.03 [0.70; 1.50] 1.0% 1.5%
Horiki et.al (2004) 22 454 50 556 0.52 [0.31; 0.86] 0.6% 1.2%
Lv et.al (2017) 372 1294 268 1300 1.55 [1.30; 1.86] 4.6% 2.1%
Fixed effect model 17906 16491 0.90 [0.82; 0.99] 16.6% --
Random effects model 0.80 [0.63; 1.00] -- 20.4%
Heterogeneity: I^2 = 78%, τ^2 = 0.1552, p < 0.01
ancestrycategory = South Asian
Kommoju et.al (2014) 176 1464 138 1188 1.04 [0.82; 1.32] 2.7% 2.0%
Phani et.al (2015) 80 1036 110 1036 0.70 [0.52; 0.95] 1.7% 1.8%
Sanghera et.al (2009) 107 1054 136 1036 0.75 [0.57; 0.98] 2.1% 1.9%
Majid et.al (2016) 17 200 42 200 0.35 [0.19; 0.64] 0.4% 1.0%
Saleh et.al (2016) 7 50 6 56 1.36 [0.42; 4.35] 0.1% 0.4%
Pattanayak et.al (2014) 36 400 47 400 0.74 [0.47; 1.17] 0.7% 1.3%
Vimaleswaran et.al (2010) 184 2000 197 2000 0.93 [0.75; 1.15] 3.4% 2.0%
Raza et.al (2012) 84 174 104 176 0.65 [0.42; 0.99] 0.8% 1.4%
Radha et.al (2006) (South Asian living in Chennai) 302 1598 323 1640 0.95 [0.80; 1.13] 4.9% 2.1%
Radha et.al (2006) (South Asian living in Dallas) 33 162 265 1232 0.93 [0.62; 1.40] 0.9% 1.5%
Fixed effect model 8138 8964 0.86 [0.79; 0.95] 17.7% --
Random effects model 0.82 [0.71; 0.95] -- 15.4%
Heterogeneity: I^2 = 51%, τ^2 = 0.0249, p = 0.03
ancestrycategory = Native American
Muller et.al (2003) 92 1314 61 656 0.73 [0.52; 1.03] 1.3% 1.7%
Fixed effect model 1314 656 0.73 [0.52; 1.03] 1.3% --
Random effects model 0.73 [0.52; 1.03] -- 1.7%
Heterogeneity: not applicable
ancestrycategory = Greater Middle Eastern
Mirzaei et.al (2009) 25 312 40 312 0.59 [0.35; 1.00] 0.5% 1.2%
Mtiraoui et.al (2012) (A) (Lebanes) 154 1502 138 1836 1.41 [1.11; 1.79] 2.6% 1.9%
Mtiraoui et.al (2012) (B) (Tunisian) 246 2940 110 1676 1.30 [1.03; 1.64] 2.8% 2.0%
Bener et.al (2015) 100 1528 86 1528 1.17 [0.87; 1.58] 1.7% 1.8%
Bouassida et.al (2005) 26 484 27 492 0.98 [0.56; 1.70] 0.5% 1.1%
Badii et.al (2008) 44 800 53 900 0.93 [0.62; 1.40] 0.9% 1.5%
Motavallian et.al (2013) 96 200 140 200 0.40 [0.26; 0.60] 0.9% 1.5%
Bouhaha et.al (2008) 15 168 30 522 1.61 [0.84; 3.07] 0.4% 0.9%
Meshkani et.al (2007) 32 568 78 824 0.57 [0.37; 0.87] 0.8% 1.4%
Erdogan et.al (2007) 1 182 4 100 0.13 [0.01; 1.20] 0.0% 0.1%
Mohamed et.al (2007) 79 982 56 800 1.16 [0.81; 1.66] 1.2% 1.6%
Namvaran et.al (2011) 6 202 18 256 0.40 [0.16; 1.04] 0.2% 0.6%
Tariq et.al (2013) 89 746 47 400 1.02 [0.70; 1.48] 1.1% 1.6%
Fixed effect model 10614 9846 1.03 [0.93; 1.14] 13.5% --
Random effects model 0.89 [0.70; 1.14] -- 17.1%
Heterogeneity: I^2 = 77%, τ^2 = 0.1361, p < 0.01
ancestrycategory = Other
Sokkar et.al (2009) 4 48 27 60 0.11 [0.04; 0.35] 0.1% 0.4%
Chistiakov et.al (2010) 207 1176 279 1194 0.70 [0.57; 0.86] 3.7% 2.1%
Mato et.al (2016) 0 120 0 120 0.0% 0.0%
Hegele et.al (2000) 38 358 56 664 1.29 [0.84; 1.99] 0.8% 1.4%
Avzaletdinova et.al (2016) 100 588 109 652 1.02 [0.76; 1.37] 1.7% 1.8%
Fixed effect model 2290 2690 0.81 [0.69; 0.94] 6.3% --
Random effects model 0.74 [0.45; 1.21] -- 5.6%
Heterogeneity: I^2 = 85%, τ^2 = 0.1892, p < 0.01
ancestrycategory = Other admixed ancestry
Vergotine et.al (2014) 29 424 55 1150 1.46 [0.92; 2.33] 0.7% 1.3%
Fixed effect model 424 1150 1.46 [0.92; 2.33] 0.7% --
Random effects model 1.46 [0.92; 2.33] -- 1.3%
Heterogeneity: not applicable
ancestrycategory = South East Asian
Paramasivam et.al (2016) 55 240 82 242 0.58 [0.39; 0.87] 0.9% 1.5%
Fixed effect model 240 242 0.58 [0.39; 0.87] 0.9% --
Random effects model 0.58 [0.39; 0.87] -- 1.5%
Heterogeneity: not applicable
ancestrycategory = Asian unspecified
Nemoto et.al (2002) (B) 4 182 10 108 0.22 [0.07; 0.72] 0.1% 0.4%
Fixed effect model 182 108 0.22 [0.07; 0.72] 0.1% --
Random effects model 0.22 [0.07; 0.72] -- 0.4%
Heterogeneity: not applicable
ancestrycategory = Hispanic or Latin American
Lara-Riegos et.al (2015) 27 252 38 252 0.68 [0.40; 1.15] 0.5% 1.2%
Martínez Gómez et.al (2011) (Combined) 192 1438 200 1492 1.00 [0.80; 1.23] 3.3% 2.0%
Fixed effect model 1690 1744 0.94 [0.77; 1.15] 3.9% --
Random effects model 0.89 [0.63; 1.26] -- 3.2%
Heterogeneity: I^2 = 44%, τ^2 = 0.0329, p = 0.18
ancestrycategory = African American or Afro-Caribbean
Kao et.al (2003) 13 872 46 2010 0.65 [0.35; 1.20] 0.4% 1.0%
Fixed effect model 872 2010 0.65 [0.35; 1.20] 0.4% --
Random effects model 0.65 [0.35; 1.20] -- 1.0%
Heterogeneity: not applicable
Fixed effect model 62250 69613 0.87 [0.84; 0.90] 100.0% --
Random effects model 0.82 [0.76; 0.89] -- 100.0%
Heterogeneity: I^2 = 71%, τ^2 = 0.0712, p < 0.01
0.1 0.5 1 2 10

A

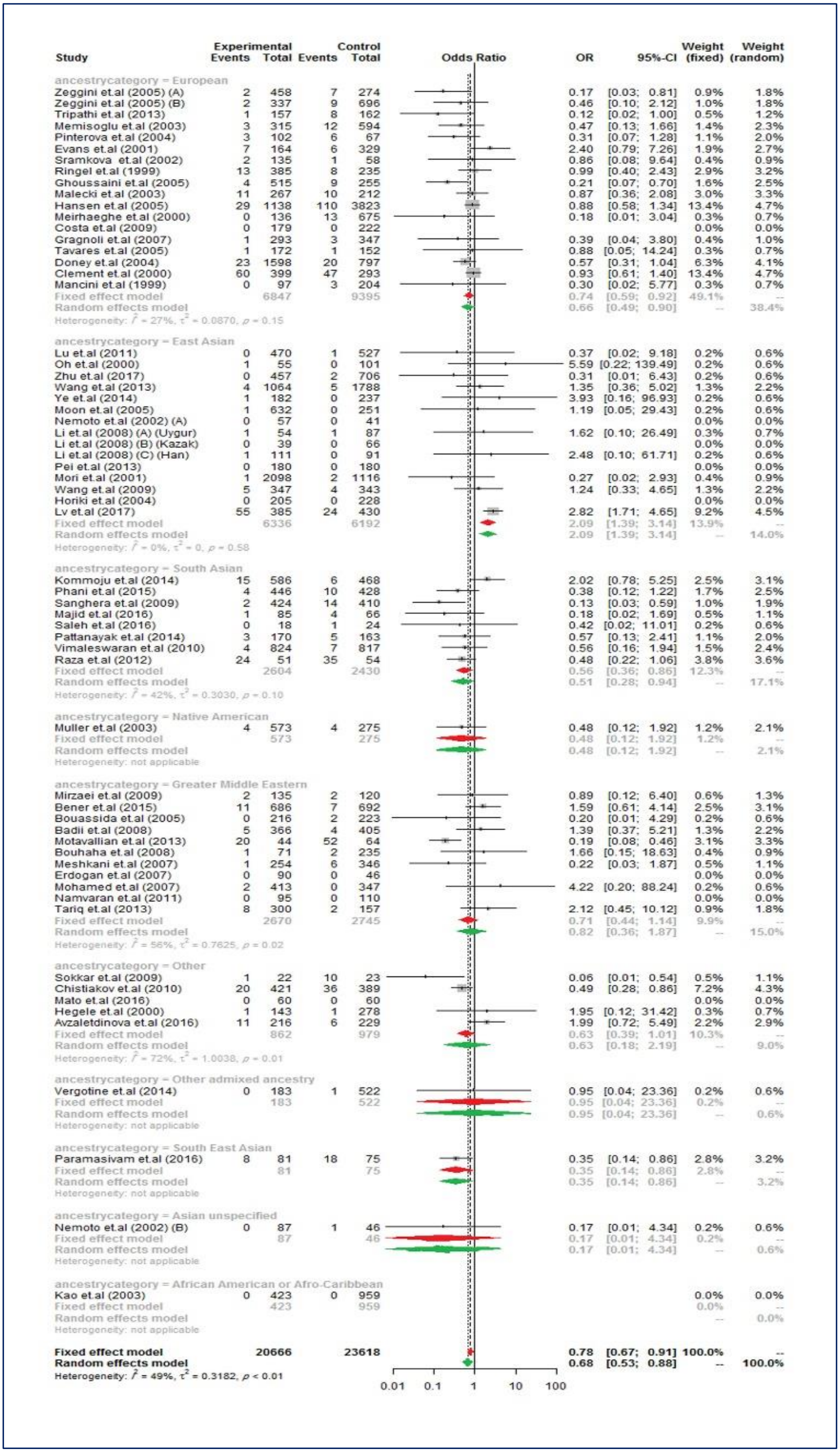
Study
Experimental Events Total
Control Events Total
Odds Ratio
OR 95%-CI
Weight (fixed)
Weight (random)
ancestrycategory = European
Zeggini et.al (2005) (A) 2 458 7 274 0.17 [0.03; 0.81] 0.9% 1.8%
Zeggini et.al (2005) (B) 2 337 9 696 0.46 [0.10; 2.12] 1.0% 1.8%
Tripathi et.al (2013) 1 157 8 162 0.12 [0.02; 1.00] 0.5% 1.2%
Memisoglu et.al (2003) 3 315 12 594 0.47 [0.13; 1.66] 1.4% 2.3%
Pinterova et.al (2004) 3 102 6 67 0.31 [0.07; 1.28] 1.1% 2.0%
Evans et.al (2001) 7 164 6 329 2.40 [0.79; 7.26] 1.9% 2.7%
Sramkova et.al (2002) 2 135 1 58 0.86 [0.08; 9.64] 0.4% 0.9%
Ringel et.al (1999) 13 385 8 235 0.99 [0.40; 2.43] 2.9% 3.2%
Ghoussaini et.al (2005) 4 515 9 255 0.21 [0.07; 0.70] 1.6% 2.5%
Malecki et.al (2003) 11 267 10 212 0.87 [0.36; 2.08] 3.0% 3.3%
Hansen et.al (2005) 29 1138 110 3823 0.88 [0.58; 1.34] 13.4% 4.7%
Meirhaeghe et.al (2000) 0 136 13 675 0.18 [0.01; 3.04] 0.3% 0.7%
Costa et.al (2009) 0 179 0 222 0.0% 0.0%
Gragnoli et.al (2007) 1 293 3 347 0.39 [0.04; 3.80] 0.4% 1.0%
Tavares et.al (2005) 1 172 1 152 0.88 [0.05; 14.24] 0.3% 0.7%
Doney et.al (2004) 23 1598 20 797 0.57 [0.31; 1.04] 6.3% 4.1%
Clement et.al (2000) 60 399 47 293 0.93 [0.61; 1.40] 13.4% 4.7%
Mancini et.al (1999) 0 97 3 204 0.30 [0.02; 5.77] 0.3% 0.7%
Fixed effect model 6847 9395 0.74 [0.59; 0.92] 49.1% --
Random effects model 0.66 [0.49; 0.90] -- 38.4%
Heterogeneity: I^2 = 27%, τ^2 = 0.0870, p = 0.15
ancestrycategory = East Asian
Lu et.al (2011) 0 470 1 527 0.37 [0.02; 9.18] 0.2% 0.6%
Oh et.al (2000) 1 55 0 101 5.59 [0.22; 139.49] 0.2% 0.6%
Zhu et.al (2017) 0 457 2 706 0.31 [0.01; 6.43] 0.2% 0.6%
Wang et.al (2013) 4 1064 5 1788 1.35 [0.36; 5.02] 1.3% 2.2%
Ye et.al (2014) 1 182 0 237 3.93 [0.16; 96.93] 0.2% 0.6%
Moon et.al (2005) 1 632 0 251 1.19 [0.05; 29.43] 0.2% 0.6%
Nemoto et.al (2002) (A) 0 57 0 41 0.0% 0.0%
Li et.al (2008) (A) (Uygur) 1 54 1 87 1.62 [0.10; 26.49] 0.3% 0.7%
Li et.al (2008) (B) (Kazak) 0 39 0 66 0.0% 0.0%
Li et.al (2008) (C) (Han) 1 111 0 91 2.48 [0.10; 61.71] 0.2% 0.6%
Pei et.al (2013) 0 180 0 180 0.0% 0.0%
Mori et.al (2001) 1 2098 2 1116 0.27 [0.02; 2.93] 0.4% 0.9%
Wang et.al (2009) 5 347 4 343 1.24 [0.33; 4.65] 1.3% 2.2%
Horiki et.al (2004) 0 205 0 228 0.0% 0.0%
Lv et.al (2017) 55 385 24 430 2.82 [1.71; 4.65] 9.2% 4.5%
Fixed effect model 6336 6192 2.09 [1.39; 3.14] 13.9% --
Random effects model 2.09 [1.39; 3.14] -- 14.0%
Heterogeneity: I^2 = 0%, τ^2 = 0, p = 0.58
ancestrycategory = South Asian
Kommoju et.al (2014) 15 586 6 468 2.02 [0.78; 5.25] 2.5% 3.1%
Phani et.al (2015) 4 446 10 428 0.38 [0.12; 1.22] 1.7% 2.5%
Sanghera et.al (2009) 2 424 14 410 0.13 [0.03; 0.59] 1.0% 1.9%
Majid et.al (2016) 1 85 4 66 0.18 [0.02; 1.69] 0.5% 1.1%
Saleh et.al (2016) 0 18 1 24 0.42 [0.02; 11.01] 0.2% 0.6%
Pattanayak et.al (2014) 3 170 5 163 0.57 [0.13; 2.41] 1.1% 2.0%
Vimaleswaran et.al (2010) 4 824 7 817 0.56 [0.16; 1.94] 1.5% 2.4%
Raza et.al (2012) 24 51 35 54 0.48 [0.22; 1.06] 3.8% 3.6%
Fixed effect model 2604 2430 0.56 [0.36; 0.86] 12.3% --
Random effects model 0.51 [0.28; 0.94] -- 17.1%
Heterogeneity: I^2 = 42%, τ^2 = 0.3030, p = 0.10
ancestrycategory = Native American
Muller et.al (2003) 4 573 4 275 0.48 [0.12; 1.92] 1.2% 2.1%
Fixed effect model 573 275 0.48 [0.12; 1.92] 1.2% --
Random effects model 0.48 [0.12; 1.92] -- 2.1%
Heterogeneity: not applicable
ancestrycategory = Greater Middle Eastern
Mirzaei et.al (2009) 2 135 2 120 0.89 [0.12; 6.40] 0.6% 1.3%
Bener et.al (2015) 11 686 7 692 1.59 [0.61; 4.14] 2.5% 3.1%
Bouassida et.al (2005) 0 216 2 223 0.20 [0.01; 4.29] 0.2% 0.6%
Badii et.al (2008) 5 366 4 405 1.39 [0.37; 5.21] 1.3% 2.2%
Motavallian et.al (2013) 20 44 52 64 0.19 [0.08; 0.46] 3.1% 3.3%
Bouhaha et.al (2008) 1 71 2 235 1.66 [0.15; 18.63] 0.4% 0.9%
Meshkani et.al (2007) 1 254 6 346 0.22 [0.03; 1.87] 0.5% 1.1%
Erdogan et.al (2007) 0 90 0 46 0.0% 0.0%
Mohamed et.al (2007) 2 413 0 347 4.22 [0.20; 88.24] 0.2% 0.6%
Namvaran et.al (2011) 0 95 0 110 0.0% 0.0%
Tariq et.al (2013) 8 300 2 157 2.12 [0.45; 10.12] 0.9% 1.8%
Fixed effect model 2670 2745 0.71 [0.44; 1.14] 9.9% --
Random effects model 0.82 [0.36; 1.87] -- 15.0%
Heterogeneity: I^2 = 56%, τ^2 = 0.7625, p = 0.02
ancestrycategory = Other
Sokkar et.al (2009) 1 22 10 23 0.06 [0.01; 0.54] 0.5% 1.1%
Chistiakov et.al (2010) 20 421 36 389 0.49 [0.28; 0.86] 7.2% 4.3%
Mato et.al (2016) 0 60 0 60 0.0% 0.0%
Hegele et.al (2000) 1 143 1 278 1.95 [0.12; 31.42] 0.3% 0.7%
Avzaletdinova et.al (2016) 11 216 6 229 1.99 [0.72; 5.49] 2.2% 2.9%
Fixed effect model 862 979 0.63 [0.39; 1.01] 10.3% --
Random effects model 0.63 [0.18; 2.19] -- 9.0%
Heterogeneity: I^2 = 72%, τ^2 = 1.0038, p = 0.01
ancestrycategory = Other admixed ancestry
Vergotine et.al (2014) 0 183 1 522 0.95 [0.04; 23.36] 0.2% 0.6%
Fixed effect model 183 522 0.95 [0.04; 23.36] 0.2% --
Random effects model 0.95 [0.04; 23.36] -- 0.6%
Heterogeneity: not applicable
ancestrycategory = South East Asian
Paramasivam et.al (2016) 8 81 18 75 0.35 [0.14; 0.86] 2.8% 3.2%
Fixed effect model 81 75 0.35 [0.14; 0.86] 2.8% --
Random effects model 0.35 [0.14; 0.86] -- 3.2%
Heterogeneity: not applicable
ancestrycategory = Asian unspecified
Nemoto et.al (2002) (B) 0 87 1 46 0.17 [0.01; 4.34] 0.2% 0.6%
Fixed effect model 87 46 0.17 [0.01; 4.34] 0.2% --
Random effects model 0.17 [0.01; 4.34] -- 0.6%
Heterogeneity: not applicable
ancestrycategory = African American or Afro-Caribbean
Kao et.al (2003) 0 423 0 959 0.0% 0.0%
Fixed effect model 423 959 0.0% --
Random effects model -- 0.0%
Heterogeneity: not applicable
Fixed effect model 20666 23618 0.78 [0.67; 0.91] 100.0% --
Random effects model 0.68 [0.53; 0.88] -- 100.0%
Heterogeneity: I^2 = 49%, τ^2 = 0.3182, p < 0.01
0.01 0.1 1 10 100

B

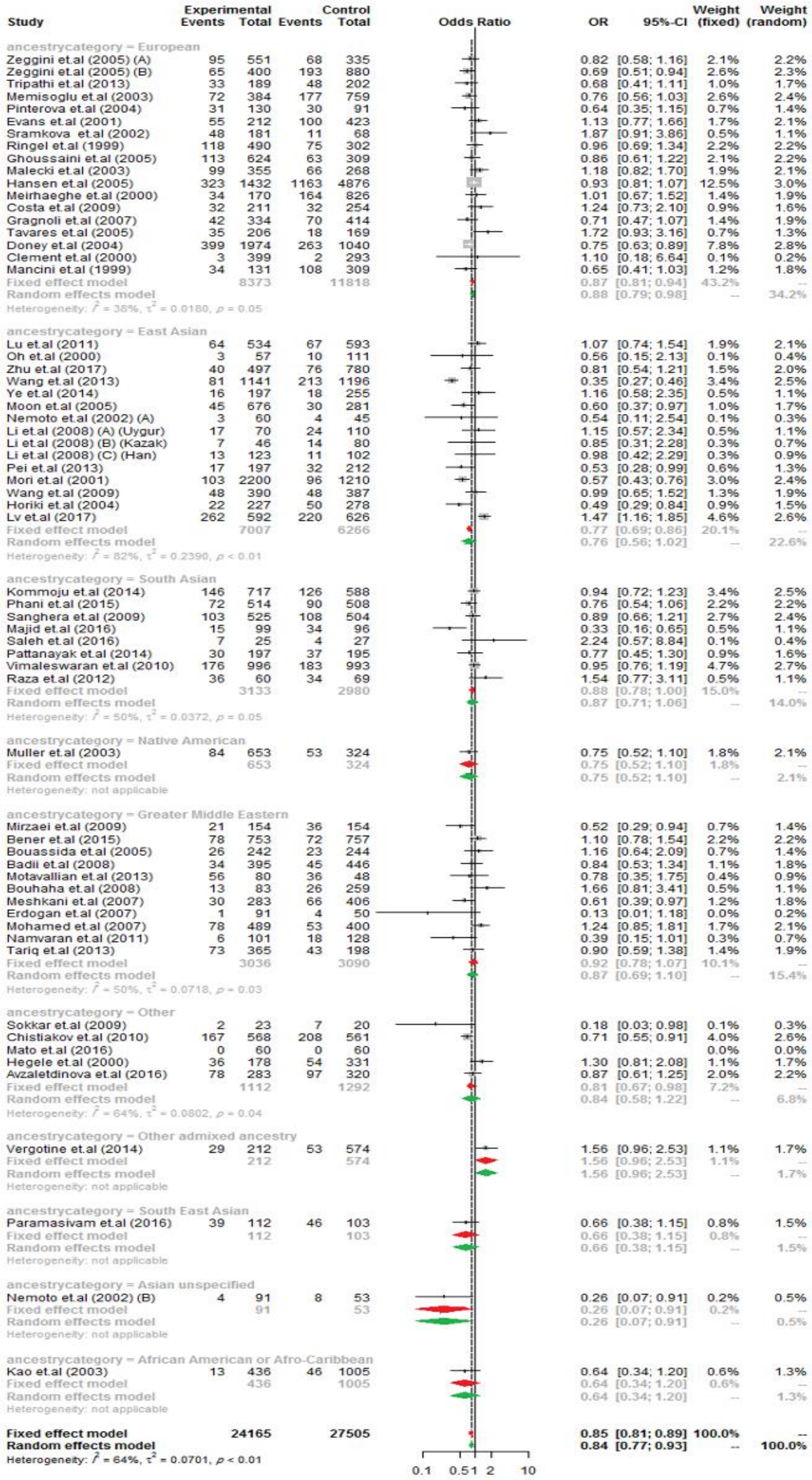
Study
Experimental Events Total
Control Events Total
Odds Ratio
OR 95%-CI Weight (fixed) Weight (random)
ancestrycategory = European
Zeggini et.al (2005) (A) 95 551 68 335 0.82 [0.58; 1.16] 2.1% 2.2%
Zeggini et.al (2005) (B) 65 400 193 880 0.69 [0.51; 0.94] 2.6% 2.3%
Tripathi et.al (2013) 33 189 48 202 0.68 [0.41; 1.11] 1.0% 1.7%
Memisoglu et.al (2003) 72 384 177 759 0.76 [0.56; 1.03] 2.6% 2.4%
Pinterova et.al (2004) 31 130 30 91 0.64 [0.35; 1.15] 0.7% 1.4%
Evans et.al (2001) 55 212 100 423 1.13 [0.77; 1.66] 1.7% 2.1%
Sramkova et.al (2002) 48 181 11 68 1.87 [0.91; 3.86] 0.5% 1.1%
Ringel et.al (1999) 118 490 75 302 0.96 [0.69; 1.34] 2.2% 2.2%
Ghoussaini et.al (2005) 113 624 63 309 0.86 [0.61; 1.22] 2.1% 2.2%
Malecki et.al (2003) 99 355 66 268 1.18 [0.82; 1.70] 1.9% 2.1%
Hansen et.al (2005) 323 1432 1163 4876 0.93 [0.81; 1.07] 12.5% 3.0%
Meirhaeghe et.al (2000) 34 170 164 826 1.01 [0.67; 1.52] 1.4% 1.9%
Costa et.al (2009) 32 211 32 254 1.24 [0.73; 2.10] 0.9% 1.6%
Gragnoli et.al (2007) 42 334 70 414 0.71 [0.47; 1.07] 1.4% 1.9%
Tavares et.al (2005) 35 206 18 169 1.72 [0.93; 3.16] 0.7% 1.3%
Doney et.al (2004) 399 1974 263 1040 0.75 [0.63; 0.89] 7.8% 2.8%
Clement et.al (2000) 3 399 2 293 1.10 [0.18; 6.64] 0.1% 0.2%
Mancini et.al (1999) 34 131 108 309 0.65 [0.41; 1.03] 1.2% 1.8%
Fixed effect model 8373 11818 0.87 [0.81; 0.94] 43.2% --
Random effects model 0.88 [0.79; 0.98] -- 34.2%
Heterogeneity: I^2 = 38%, τ^2 = 0.0180, p = 0.05
ancestrycategory = East Asian
Lu et.al (2011) 64 534 67 593 1.07 [0.74; 1.54] 1.9% 2.1%
Oh et.al (2000) 3 57 10 111 0.56 [0.15; 2.13] 0.1% 0.4%
Zhu et.al (2017) 40 497 76 780 0.81 [0.54; 1.21] 1.5% 2.0%
Wang et.al (2013) 81 1141 213 1196 0.35 [0.27; 0.46] 3.4% 2.5%
Ye et.al (2014) 16 197 18 255 1.16 [0.58; 2.35] 0.5% 1.1%
Moon et.al (2005) 45 676 30 281 0.60 [0.37; 0.97] 1.0% 1.7%
Nemoto et.al (2002) (A) 3 60 4 45 0.54 [0.11; 2.54] 0.1% 0.3%
Li et.al (2008) (A) (Uygur) 17 70 24 110 1.15 [0.57; 2.34] 0.5% 1.1%
Li et.al (2008) (B) (Kazak) 7 46 14 80 0.85 [0.31; 2.28] 0.3% 0.7%
Li et.al (2008) (C) (Han) 13 123 11 102 0.98 [0.42; 2.29] 0.3% 0.9%
Pei et.al (2013) 17 197 32 212 0.53 [0.28; 0.99] 0.6% 1.3%
Mori et.al (2001) 103 2200 96 1210 0.57 [0.43; 0.76] 3.0% 2.4%
Wang et.al (2009) 48 390 48 387 0.99 [0.65; 1.52] 1.3% 1.9%
Horiki et.al (2004) 22 227 50 278 0.49 [0.29; 0.84] 0.9% 1.5%
Lv et.al (2017) 262 592 220 626 1.47 [1.16; 1.85] 4.6% 2.6%
Fixed effect model 7007 6266 0.77 [0.69; 0.86] 20.1% --
Random effects model 0.76 [0.56; 1.02] -- 22.6%
Heterogeneity: I^2 = 82%, τ^2 = 0.2390, p < 0.01
ancestrycategory = South Asian
Kommoju et.al (2014) 146 717 126 588 0.94 [0.72; 1.23] 3.4% 2.5%
Phani et.al (2015) 72 514 90 508 0.76 [0.54; 1.06] 2.2% 2.2%
Sanghera et.al (2009) 103 525 108 504 0.89 [0.66; 1.21] 2.7% 2.4%
Majid et.al (2016) 15 99 34 96 0.33 [0.16; 0.65] 0.5% 1.1%
Saleh et.al (2016) 7 25 4 27 2.24 [0.57; 8.84] 0.1% 0.4%
Pattanayak et.al (2014) 30 197 37 195 0.77 [0.45; 1.30] 0.9% 1.6%
Vimaleswaran et.al (2010) 176 996 183 993 0.95 [0.76; 1.19] 4.7% 2.7%
Raza et.al (2012) 36 60 34 69 1.54 [0.77; 3.11] 0.5% 1.1%
Fixed effect model 3133 2980 0.88 [0.78; 1.00] 15.0% --
Random effects model 0.87 [0.71; 1.06] -- 14.0%
Heterogeneity: I^2 = 50%, τ^2 = 0.0372, p = 0.05
ancestrycategory = Native American
Muller et.al (2003) 84 653 53 324 0.75 [0.52; 1.10] 1.8% 2.1%
Fixed effect model 653 324 0.75 [0.52; 1.10] 1.8% --
Random effects model 0.75 [0.52; 1.10] -- 2.1%
Heterogeneity: not applicable
ancestrycategory = Greater Middle Eastern
Mirzaei et.al (2009) 21 154 36 154 0.52 [0.29; 0.94] 0.7% 1.4%
Bener et.al (2015) 78 753 72 757 1.10 [0.78; 1.54] 2.2% 2.2%
Bouassida et.al (2005) 26 242 23 244 1.16 [0.64; 2.09] 0.7% 1.4%
Badii et.al (2008) 34 395 45 446 0.84 [0.53; 1.34] 1.1% 1.8%
Motavallian et.al (2013) 56 80 36 48 0.78 [0.35; 1.75] 0.4% 0.9%
Bouhaha et.al (2008) 13 83 26 259 1.66 [0.81; 3.41] 0.5% 1.1%
Meshkani et.al (2007) 30 283 66 406 0.61 [0.39; 0.97] 1.2% 1.8%
Erdogan et.al (2007) 1 91 4 50 0.13 [0.01; 1.18] 0.0% 0.2%
Mohamed et.al (2007) 78 489 53 400 1.24 [0.85; 1.81] 1.7% 2.1%
Namvaran et.al (2011) 6 101 18 128 0.39 [0.15; 1.01] 0.3% 0.7%
Tariq et.al (2013) 73 365 43 198 0.90 [0.59; 1.38] 1.4% 1.9%
Fixed effect model 3036 3090 0.92 [0.78; 1.07] 10.1% --
Random effects model 0.87 [0.69; 1.10] -- 15.4%
Heterogeneity: I^2 = 50%, τ^2 = 0.0718, p = 0.03
ancestrycategory = Other
Sokkar et.al (2009) 2 23 7 20 0.18 [0.03; 0.98] 0.1% 0.3%
Chistiakov et.al (2010) 167 568 208 561 0.71 [0.55; 0.91] 4.0% 2.6%
Mato et.al (2016) 0 60 0 60 0.0% 0.0%
Hegele et.al (2000) 36 178 54 331 1.30 [0.81; 2.08] 1.1% 1.7%
Avzaletdinova et.al (2016) 78 283 97 320 0.87 [0.61; 1.25] 2.0% 2.2%
Fixed effect model 1112 1292 0.81 [0.67; 0.98] 7.2% --
Random effects model 0.84 [0.58; 1.22] -- 6.8%
Heterogeneity: I^2 = 64%, τ^2 = 0.0802, p = 0.04
ancestrycategory = Other admixed ancestry
Vergotine et.al (2014) 29 212 53 574 1.56 [0.96; 2.53] 1.1% 1.7%
Fixed effect model 212 574 1.56 [0.96; 2.53] 1.1% --
Random effects model 1.56 [0.96; 2.53] -- 1.7%
Heterogeneity: not applicable
ancestrycategory = South East Asian
Paramasivam et.al (2016) 39 112 46 103 0.66 [0.38; 1.15] 0.8% 1.5%
Fixed effect model 112 103 0.66 [0.38; 1.15] 0.8% --
Random effects model 0.66 [0.38; 1.15] -- 1.5%
Heterogeneity: not applicable
ancestrycategory = Asian unspecified
Nemoto et.al (2002) (B) 4 91 8 53 0.26 [0.07; 0.91] 0.2% 0.5%
Fixed effect model 91 53 0.26 [0.07; 0.91] 0.2% --
Random effects model 0.26 [0.07; 0.91] -- 0.5%
Heterogeneity: not applicable
ancestrycategory = African American or Afro-Caribbean
Kao et.al (2003) 13 436 46 1005 0.64 [0.34; 1.20] 0.6% 1.3%
Fixed effect model 436 1005 0.64 [0.34; 1.20] 0.6% --
Random effects model 0.64 [0.34; 1.20] -- 1.3%
Heterogeneity: not applicable
Fixed effect model 24165 27505 0.85 [0.81; 0.89] 100.0% --
Random effects model 0.84 [0.77; 0.93] -- 100.0%
Heterogeneity: I^2 = 64%, τ^2 = 0.0701, p < 0.01
0.1 0.51 2 10

C

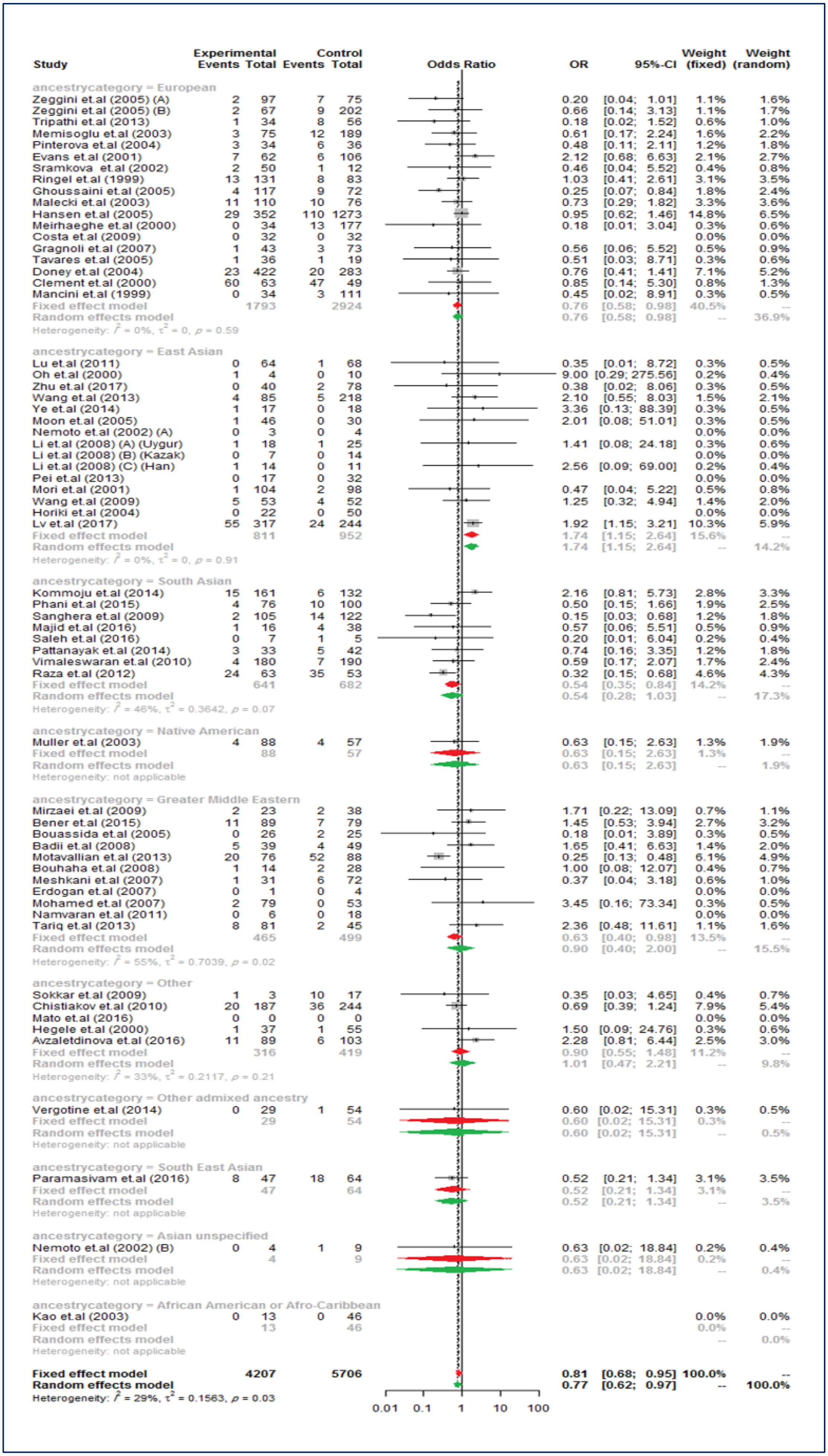
Study | Experimental Events Total | Control Events Total | Odds Ratio | OR | 95%-CI | Weight (fixed) | Weight (random)
ancestrycategory = European
Zeggini et.al (2005) (A) 2 97 7 75 0.20 [0.04; 1.01] 1.1% 1.6%
Zeggini et.al (2005) (B) 2 67 9 202 0.66 [0.14; 3.13] 1.1% 1.7%
Tripathi et.al (2013) 1 34 8 56 0.18 [0.02; 1.52] 0.6% 1.0%
Memisoglu et.al (2003) 3 75 12 189 0.61 [0.17; 2.24] 1.6% 2.2%
Pinterova et.al (2004) 3 34 6 36 0.48 [0.11; 2.11] 1.2% 1.8%
Evans et.al (2001) 7 62 6 106 2.12 [0.68; 6.63] 2.1% 2.7%
Sramkova et.al (2002) 2 50 1 12 0.46 [0.04; 5.52] 0.4% 0.8%
Ringel et.al (1999) 13 131 8 83 1.03 [0.41; 2.61] 3.1% 3.5%
Ghoussaini et.al (2005) 4 117 9 72 0.25 [0.07; 0.84] 1.8% 2.4%
Malecki et.al (2003) 11 110 10 76 0.73 [0.29; 1.82] 3.3% 3.6%
Hansen et.al (2005) 29 352 110 1273 0.95 [0.62; 1.46] 14.8% 6.5%
Meirhaeghe et.al (2000) 0 34 13 177 0.18 [0.01; 3.04] 0.3% 0.6%
Costa et.al (2009) 0 32 0 32 0.0% 0.0%
Gragnoli et.al (2007) 1 43 3 73 0.56 [0.06; 5.52] 0.5% 0.9%
Tavares et.al (2005) 1 36 1 19 0.51 [0.03; 8.71] 0.3% 0.6%
Doney et.al (2004) 23 422 20 283 0.76 [0.41; 1.41] 7.1% 5.2%
Clement et.al (2000) 60 63 47 49 0.85 [0.14; 5.30] 0.8% 1.3%
Mancini et.al (1999) 0 34 3 111 0.45 [0.02; 8.91] 0.3% 0.5%
Fixed effect model 1793 2924 0.76 [0.58; 0.98] 40.5% --
Random effects model 0.76 [0.58; 0.98] -- 36.9%
Heterogeneity: I^2 = 0%, τ^2 = 0, p = 0.59
ancestrycategory = East Asian
Lu et.al (2011) 0 64 1 68 0.35 [0.01; 8.72] 0.3% 0.5%
Oh et.al (2000) 1 4 0 10 9.00 [0.29; 275.56] 0.2% 0.4%
Zhu et.al (2017) 0 40 2 78 0.38 [0.02; 8.06] 0.3% 0.5%
Wang et.al (2013) 4 85 5 218 2.10 [0.55; 8.03] 1.5% 2.1%
Ye et.al (2014) 1 17 0 18 3.36 [0.13; 88.39] 0.3% 0.5%
Moon et.al (2005) 1 46 0 30 2.01 [0.08; 51.01] 0.3% 0.5%
Nemoto et.al (2002) (A) 0 3 0 4 0.0% 0.0%
Li et.al (2008) (A) (Uygur) 1 18 1 25 1.41 [0.08; 24.18] 0.3% 0.6%
Li et.al (2008) (B) (Kazak) 0 7 0 14 0.0% 0.0%
Li et.al (2008) (C) (Han) 1 14 0 11 2.56 [0.09; 69.00] 0.2% 0.4%
Pei et.al (2013) 0 17 0 32 0.0% 0.0%
Mori et.al (2001) 1 104 2 98 0.47 [0.04; 5.22] 0.5% 0.8%
Wang et.al (2009) 5 53 4 52 1.25 [0.32; 4.94] 1.4% 2.0%
Horiki et.al (2004) 0 22 0 50 0.0% 0.0%
Lv et.al (2017) 55 317 24 244 1.92 [1.15; 3.21] 10.3% 5.9%
Fixed effect model 811 952 1.74 [1.15; 2.64] 15.6% --
Random effects model 1.74 [1.15; 2.64] -- 14.2%
Heterogeneity: I^2 = 0%, τ^2 = 0, p = 0.91
ancestrycategory = South Asian
Kommoju et.al (2014) 15 161 6 132 2.16 [0.81; 5.73] 2.8% 3.3%
Phani et.al (2015) 4 76 10 100 0.50 [0.15; 1.66] 1.9% 2.5%
Sanghera et.al (2009) 2 105 14 122 0.15 [0.03; 0.68] 1.2% 1.8%
Majid et.al (2016) 1 16 4 38 0.57 [0.06; 5.51] 0.5% 0.9%
Saleh et.al (2016) 0 7 1 5 0.20 [0.01; 6.04] 0.2% 0.4%
Pattanayak et.al (2014) 3 33 5 42 0.74 [0.16; 3.35] 1.2% 1.8%
Vimaleswaran et.al (2010) 4 180 7 190 0.59 [0.17; 2.07] 1.7% 2.4%
Raza et.al (2012) 24 63 35 53 0.32 [0.15; 0.68] 4.6% 4.3%
Fixed effect model 641 682 0.54 [0.35; 0.84] 14.2% --
Random effects model 0.54 [0.28; 1.03] -- 17.3%
Heterogeneity: I^2 = 46%, τ^2 = 0.3642, p = 0.07
ancestrycategory = Native American
Muller et.al (2003) 4 88 4 57 0.63 [0.15; 2.63] 1.3% 1.9%
Fixed effect model 88 57 0.63 [0.15; 2.63] 1.3% --
Random effects model 0.63 [0.15; 2.63] -- 1.9%
Heterogeneity: not applicable
ancestrycategory = Greater Middle Eastern
Mirzaei et.al (2009) 2 23 2 38 1.71 [0.22; 13.09] 0.7% 1.1%
Bener et.al (2015) 11 89 7 79 1.45 [0.53; 3.94] 2.7% 3.2%
Bouassida et.al (2005) 0 26 2 25 0.18 [0.01; 3.89] 0.3% 0.5%
Badii et.al (2008) 5 39 4 49 1.65 [0.41; 6.63] 1.4% 2.0%
Motavallian et.al (2013) 20 76 52 88 0.25 [0.13; 0.48] 6.1% 4.9%
Bouhaha et.al (2008) 1 14 2 28 1.00 [0.08; 12.07] 0.4% 0.7%
Meshkani et.al (2007) 1 31 6 72 0.37 [0.04; 3.18] 0.6% 1.0%
Erdogan et.al (2007) 0 1 0 4 0.0% 0.0%
Mohamed et.al (2007) 2 79 0 53 3.45 [0.16; 73.34] 0.3% 0.5%
Namvaran et.al (2011) 0 6 0 18 0.0% 0.0%
Tariq et.al (2013) 8 81 2 45 2.36 [0.48; 11.61] 1.1% 1.6%
Fixed effect model 465 499 0.63 [0.40; 0.98] 13.5% --
Random effects model 0.90 [0.40; 2.00] -- 15.5%
Heterogeneity: I^2 = 55%, τ^2 = 0.7039, p = 0.02
ancestrycategory = Other
Sokkar et.al (2009) 1 3 10 17 0.35 [0.03; 4.65] 0.4% 0.7%
Chistiakov et.al (2010) 20 187 36 244 0.69 [0.39; 1.24] 7.9% 5.4%
Mato et.al (2016) 0 0 0 0 0.0% 0.0%
Hegele et.al (2000) 1 37 1 55 1.50 [0.09; 24.76] 0.3% 0.6%
Avzaletdinova et.al (2016) 11 89 6 103 2.28 [0.81; 6.44] 2.5% 3.0%
Fixed effect model 316 419 0.90 [0.55; 1.48] 11.2% --
Random effects model 1.01 [0.47; 2.21] -- 9.8%
Heterogeneity: I^2 = 33%, τ^2 = 0.2117, p = 0.21
ancestrycategory = Other admixed ancestry
Vergotine et.al (2014) 0 29 1 54 0.60 [0.02; 15.31] 0.3% 0.5%
Fixed effect model 29 54 0.60 [0.02; 15.31] 0.3% --
Random effects model 0.60 [0.02; 15.31] -- 0.5%
Heterogeneity: not applicable
ancestrycategory = South East Asian
Paramasivam et.al (2016) 8 47 18 64 0.52 [0.21; 1.34] 3.1% 3.5%
Fixed effect model 47 64 0.52 [0.21; 1.34] 3.1% --
Random effects model 0.52 [0.21; 1.34] -- 3.5%
Heterogeneity: not applicable
ancestrycategory = Asian unspecified
Nemoto et.al (2002) (B) 0 4 1 9 0.63 [0.02; 18.84] 0.2% 0.4%
Fixed effect model 4 9 0.63 [0.02; 18.84] 0.2% --
Random effects model 0.63 [0.02; 18.84] -- 0.4%
Heterogeneity: not applicable
ancestrycategory = African American or Afro-Caribbean
Kao et.al (2003) 0 13 0 46 0.0% 0.0%
Fixed effect model 13 46 0.0% --
Random effects model -- 0.0%
Heterogeneity: not applicable
Fixed effect model 4207 5706 0.81 [0.68; 0.95] 100.0% --
Random effects model 0.77 [0.62; 0.97] -- 100.0%
Heterogeneity: I^2 = 29%, τ^2 = 0.1563, p = 0.03
0.01 0.1 1 10 100

D

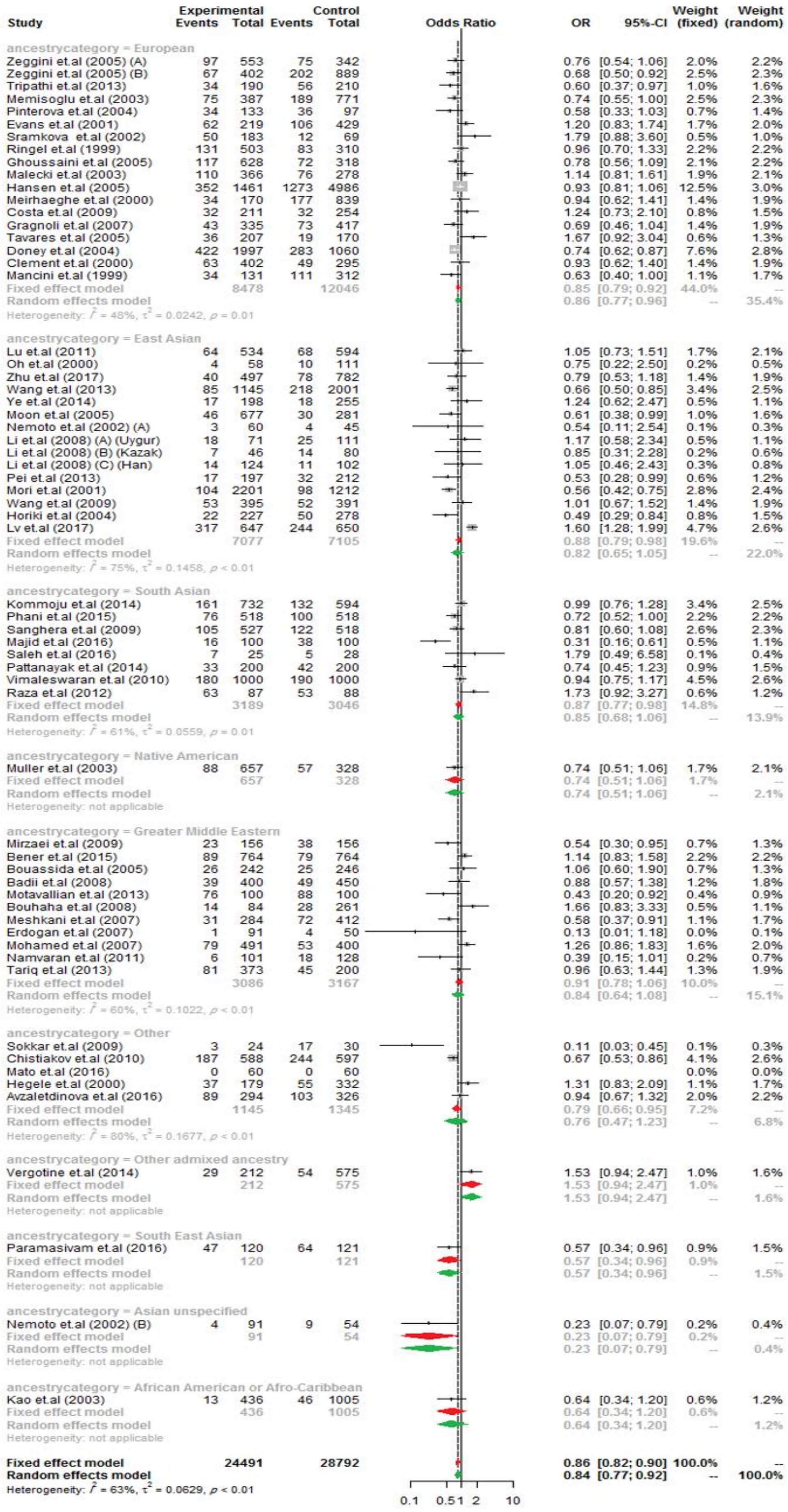
Study Experimental Events Total Control Events Total Odds Ratio OR 95%-CI Weight (fixed) Weight (random)
ancestrycategory = European
Zeggini et.al (2005) (A) 97 553 75 342 0.76 [0.54; 1.06] 2.0% 2.2%
Zeggini et.al (2005) (B) 67 402 202 889 0.68 [0.50; 0.92] 2.5% 2.3%
Tripathi et.al (2013) 34 190 56 210 0.60 [0.37; 0.97] 1.0% 1.6%
Memisoglu et.al (2003) 75 387 189 771 0.74 [0.55; 1.00] 2.5% 2.3%
Pinterova et.al (2004) 34 133 36 97 0.58 [0.33; 1.03] 0.7% 1.4%
Evans et.al (2001) 62 219 106 429 1.20 [0.83; 1.74] 1.7% 2.0%
Sramkova et.al (2002) 50 183 12 69 1.79 [0.88; 3.60] 0.5% 1.0%
Ringel et.al (1999) 131 503 83 310 0.96 [0.70; 1.33] 2.2% 2.2%
Ghoussaini et.al (2005) 117 628 72 318 0.78 [0.56; 1.09] 2.1% 2.2%
Malecki et.al (2003) 110 366 76 278 1.14 [0.81; 1.61] 1.9% 2.1%
Hansen et.al (2005) 352 1461 1273 4986 0.93 [0.81; 1.06] 12.5% 3.0%
Meirhaeghe et.al (2000) 34 170 177 839 0.94 [0.62; 1.41] 1.4% 1.9%
Costa et.al (2009) 32 211 32 254 1.24 [0.73; 2.10] 0.8% 1.5%
Gragnoli et.al (2007) 43 335 73 417 0.69 [0.46; 1.04] 1.4% 1.9%
Tavares et.al (2005) 36 207 19 170 1.67 [0.92; 3.04] 0.6% 1.3%
Doney et.al (2004) 422 1997 283 1060 0.74 [0.62; 0.87] 7.6% 2.8%
Clement et.al (2000) 63 402 49 295 0.93 [0.62; 1.40] 1.4% 1.9%
Mancini et.al (1999) 34 131 111 312 0.63 [0.40; 1.00] 1.1% 1.7%
Fixed effect model 8478 12046 0.85 [0.79; 0.92] 44.0% --
Random effects model 0.86 [0.77; 0.96] -- 35.4%
Heterogeneity: I^2 = 48%, τ^2 = 0.0242, p = 0.01
ancestrycategory = East Asian
Lu et.al (2011) 64 534 68 594 1.05 [0.73; 1.51] 1.7% 2.1%
Oh et.al (2000) 4 58 10 111 0.75 [0.22; 2.50] 0.2% 0.5%
Zhu et.al (2017) 40 497 78 782 0.79 [0.53; 1.18] 1.4% 1.9%
Wang et.al (2013) 85 1145 218 2001 0.66 [0.50; 0.85] 3.4% 2.5%
Ye et.al (2014) 17 198 18 255 1.24 [0.62; 2.47] 0.5% 1.1%
Moon et.al (2005) 46 677 30 281 0.61 [0.38; 0.99] 1.0% 1.6%
Nemoto et.al (2002) (A) 3 60 4 45 0.54 [0.11; 2.54] 0.1% 0.3%
Li et.al (2008) (A) (Uygur) 18 71 25 111 1.17 [0.58; 2.34] 0.5% 1.1%
Li et.al (2008) (B) (Kazak) 7 46 14 80 0.85 [0.31; 2.28] 0.2% 0.6%
Li et.al (2008) (C) (Han) 14 124 11 102 1.05 [0.46; 2.43] 0.3% 0.8%
Pei et.al (2013) 17 197 32 212 0.53 [0.28; 0.99] 0.6% 1.2%
Mori et.al (2001) 104 2201 98 1212 0.56 [0.42; 0.75] 2.8% 2.4%
Wang et.al (2009) 53 395 52 391 1.01 [0.67; 1.52] 1.4% 1.9%
Horiki et.al (2004) 22 227 50 278 0.49 [0.29; 0.84] 0.8% 1.5%
Lv et.al (2017) 317 647 244 650 1.60 [1.28; 1.99] 4.7% 2.6%
Fixed effect model 7077 7105 0.88 [0.79; 0.98] 19.6% --
Random effects model 0.82 [0.65; 1.05] -- 22.0%
Heterogeneity: I^2 = 75%, τ^2 = 0.1458, p < 0.01
ancestrycategory = South Asian
Kommoju et.al (2014) 161 732 132 594 0.99 [0.76; 1.28] 3.4% 2.5%
Phani et.al (2015) 76 518 100 518 0.72 [0.52; 1.00] 2.2% 2.2%
Sanghera et.al (2009) 105 527 122 518 0.81 [0.60; 1.08] 2.6% 2.3%
Majid et.al (2016) 16 100 38 100 0.31 [0.16; 0.61] 0.5% 1.1%
Saleh et.al (2016) 7 25 5 28 1.79 [0.49; 6.58] 0.1% 0.4%
Pattanayak et.al (2014) 33 200 42 200 0.74 [0.45; 1.23] 0.9% 1.5%
Vimaleswaran et.al (2010) 180 1000 190 1000 0.94 [0.75; 1.17] 4.5% 2.6%
Raza et.al (2012) 63 87 53 88 1.73 [0.92; 3.27] 0.6% 1.2%
Fixed effect model 3189 3046 0.87 [0.77; 0.98] 14.8% --
Random effects model 0.85 [0.68; 1.06] -- 13.9%
Heterogeneity: I^2 = 61%, τ^2 = 0.0559, p = 0.01
ancestrycategory = Native American
Muller et.al (2003) 88 657 57 328 0.74 [0.51; 1.06] 1.7% 2.1%
Fixed effect model 657 328 0.74 [0.51; 1.06] 1.7% --
Random effects model 0.74 [0.51; 1.06] -- 2.1%
Heterogeneity: not applicable
ancestrycategory = Greater Middle Eastern
Mirzaei et.al (2009) 23 156 38 156 0.54 [0.30; 0.95] 0.7% 1.3%
Bener et.al (2015) 89 764 79 764 1.14 [0.83; 1.58] 2.2% 2.2%
Bouassida et.al (2005) 26 242 25 246 1.06 [0.60; 1.90] 0.7% 1.3%
Badii et.al (2008) 39 400 49 450 0.88 [0.57; 1.38] 1.2% 1.8%
Motavallian et.al (2013) 76 100 88 100 0.43 [0.20; 0.92] 0.4% 0.9%
Bouhaha et.al (2008) 14 84 28 261 1.66 [0.83; 3.33] 0.5% 1.1%
Meshkani et.al (2007) 31 284 72 412 0.58 [0.37; 0.91] 1.1% 1.7%
Erdogan et.al (2007) 1 91 4 50 0.13 [0.01; 1.18] 0.0% 0.1%
Mohamed et.al (2007) 79 491 53 400 1.26 [0.86; 1.83] 1.6% 2.0%
Namvaran et.al (2011) 6 101 18 128 0.39 [0.15; 1.01] 0.2% 0.7%
Tariq et.al (2013) 81 373 45 200 0.96 [0.63; 1.44] 1.3% 1.9%
Fixed effect model 3086 3167 0.91 [0.78; 1.06] 10.0% --
Random effects model 0.84 [0.64; 1.08] -- 15.1%
Heterogeneity: I^2 = 60%, τ^2 = 0.1022, p < 0.01
ancestrycategory = Other
Sokkar et.al (2009) 3 24 17 30 0.11 [0.03; 0.45] 0.1% 0.3%
Chistiakov et.al (2010) 187 588 244 597 0.67 [0.53; 0.86] 4.1% 2.6%
Mato et.al (2016) 0 60 0 60 0.0% 0.0%
Hegele et.al (2000) 37 179 55 332 1.31 [0.83; 2.09] 1.1% 1.7%
Avzaletdinova et.al (2016) 89 294 103 326 0.94 [0.67; 1.32] 2.0% 2.2%
Fixed effect model 1145 1345 0.79 [0.66; 0.95] 7.2% --
Random effects model 0.76 [0.47; 1.23] -- 6.8%
Heterogeneity: I^2 = 80%, τ^2 = 0.1677, p < 0.01
ancestrycategory = Other admixed ancestry
Vergotine et.al (2014) 29 212 54 575 1.53 [0.94; 2.47] 1.0% 1.6%
Fixed effect model 212 575 1.53 [0.94; 2.47] 1.0% --
Random effects model 1.53 [0.94; 2.47] -- 1.6%
Heterogeneity: not applicable
ancestrycategory = South East Asian
Paramasivam et.al (2016) 47 120 64 121 0.57 [0.34; 0.96] 0.9% 1.5%
Fixed effect model 120 121 0.57 [0.34; 0.96] 0.9% --
Random effects model 0.57 [0.34; 0.96] -- 1.5%
Heterogeneity: not applicable
ancestrycategory = Asian unspecified
Nemoto et.al (2002) (B) 4 91 9 54 0.23 [0.07; 0.79] 0.2% 0.4%
Fixed effect model 91 54 0.23 [0.07; 0.79] 0.2% --
Random effects model 0.23 [0.07; 0.79] -- 0.4%
Heterogeneity: not applicable
ancestrycategory = African American or Afro-Caribbean
Kao et.al (2003) 13 436 46 1005 0.64 [0.34; 1.20] 0.6% 1.2%
Fixed effect model 436 1005 0.64 [0.34; 1.20] 0.6% --
Random effects model 0.64 [0.34; 1.20] -- 1.2%
Heterogeneity: not applicable
Fixed effect model 24491 28792 0.86 [0.82; 0.90] 100.0% --
Random effects model 0.84 [0.77; 0.92] -- 100.0%
Heterogeneity: I^2 = 63%, τ^2 = 0.0629, p < 0.01
0.1 0.5 1 2 10

E

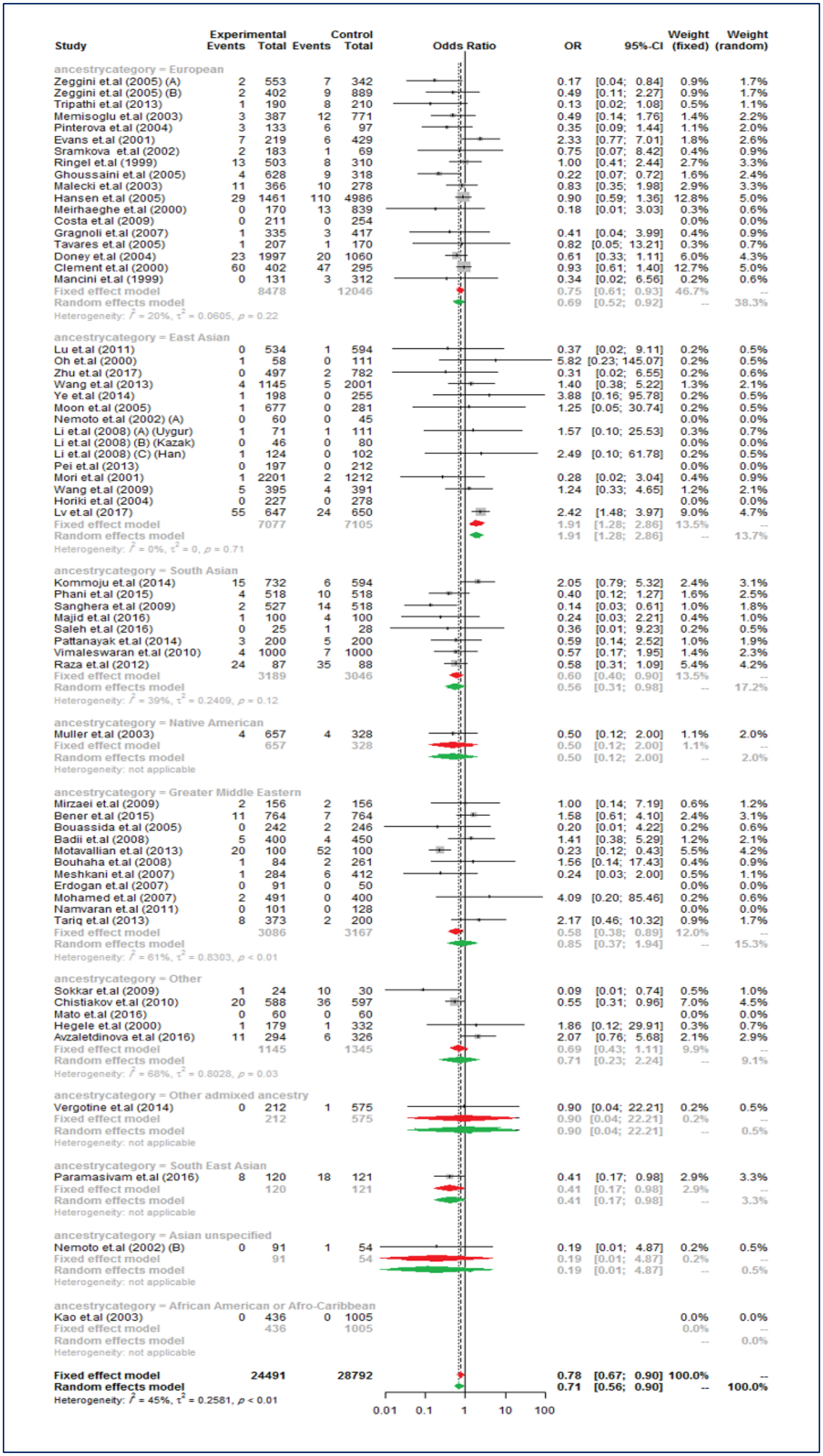
Study | Experimental Events Total | Control Events Total | Odds Ratio | OR | 95%-CI | Weight (fixed) | Weight (random)
ancestrycategory = European
Zeggini et.al (2005) (A) 2 553 7 342 0.17 [0.04; 0.84] 0.9% 1.7%
Zeggini et.al (2005) (B) 2 402 9 889 0.49 [0.11; 2.27] 0.9% 1.7%
Tripathi et.al (2013) 1 190 8 210 0.13 [0.02; 1.08] 0.5% 1.1%
Memisoglu et.al (2003) 3 387 12 771 0.49 [0.14; 1.76] 1.4% 2.2%
Pinterova et.al (2004) 3 133 6 97 0.35 [0.09; 1.44] 1.1% 2.0%
Evans et.al (2001) 7 219 6 429 2.33 [0.77; 7.01] 1.8% 2.6%
Sramkova et.al (2002) 2 183 1 69 0.75 [0.07; 8.42] 0.4% 0.9%
Ringel et.al (1999) 13 503 8 310 1.00 [0.41; 2.44] 2.7% 3.3%
Ghoussaini et.al (2005) 4 628 9 318 0.22 [0.07; 0.72] 1.6% 2.4%
Malecki et.al (2003) 11 366 10 278 0.83 [0.35; 1.98] 2.9% 3.3%
Hansen et.al (2005) 29 1461 110 4986 0.90 [0.59; 1.36] 12.8% 5.0%
Meirhaeghe et.al (2000) 0 170 13 839 0.18 [0.01; 3.03] 0.3% 0.6%
Costa et.al (2009) 0 211 0 254 0.0% 0.0%
Gragnoli et.al (2007) 1 335 3 417 0.41 [0.04; 3.99] 0.4% 0.9%
Tavares et.al (2005) 1 207 1 170 0.82 [0.05; 13.21] 0.3% 0.7%
Doney et.al (2004) 23 1997 20 1060 0.61 [0.33; 1.11] 6.0% 4.3%
Clement et.al (2000) 60 402 47 295 0.93 [0.61; 1.40] 12.7% 5.0%
Mancini et.al (1999) 0 131 3 312 0.34 [0.02; 6.56] 0.2% 0.6%
Fixed effect model 8478 12046 0.75 [0.61; 0.93] 46.7% --
Random effects model 0.69 [0.52; 0.92] -- 38.3%
Heterogeneity: I^2 = 20%, τ^2 = 0.0605, p = 0.22
ancestrycategory = East Asian
Lu et.al (2011) 0 534 1 594 0.37 [0.02; 9.11] 0.2% 0.5%
Oh et.al (2000) 1 58 0 111 5.82 [0.23; 145.07] 0.2% 0.5%
Zhu et.al (2017) 0 497 2 782 0.31 [0.02; 6.55] 0.2% 0.6%
Wang et.al (2013) 4 1145 5 2001 1.40 [0.38; 5.22] 1.3% 2.1%
Ye et.al (2014) 1 198 0 255 3.88 [0.16; 95.78] 0.2% 0.5%
Moon et.al (2005) 1 677 0 281 1.25 [0.05; 30.74] 0.2% 0.5%
Nemoto et.al (2002) (A) 0 60 0 45 0.0% 0.0%
Li et.al (2008) (A) (Uygur) 1 71 1 111 1.57 [0.10; 25.53] 0.3% 0.7%
Li et.al (2008) (B) (Kazak) 0 46 0 80 0.0% 0.0%
Li et.al (2008) (C) (Han) 1 124 0 102 2.49 [0.10; 61.78] 0.2% 0.5%
Pei et.al (2013) 0 197 0 212 0.0% 0.0%
Mori et.al (2001) 1 2201 2 1212 0.28 [0.02; 3.04] 0.4% 0.9%
Wang et.al (2009) 5 395 4 391 1.24 [0.33; 4.65] 1.2% 2.1%
Horiki et.al (2004) 0 227 0 278 0.0% 0.0%
Lv et.al (2017) 55 647 24 650 2.42 [1.48; 3.97] 9.0% 4.7%
Fixed effect model 7077 7105 1.91 [1.28; 2.86] 13.5% --
Random effects model 1.91 [1.28; 2.86] -- 13.7%
Heterogeneity: I^2 = 0%, τ^2 = 0, p = 0.71
ancestrycategory = South Asian
Kommoju et.al (2014) 15 732 6 594 2.05 [0.79; 5.32] 2.4% 3.1%
Phani et.al (2015) 4 518 10 518 0.40 [0.12; 1.27] 1.6% 2.5%
Sanghera et.al (2009) 2 527 14 518 0.14 [0.03; 0.61] 1.0% 1.8%
Majid et.al (2016) 1 100 4 100 0.24 [0.03; 2.21] 0.4% 1.0%
Saleh et.al (2016) 0 25 1 28 0.36 [0.01; 9.23] 0.2% 0.5%
Pattanayak et.al (2014) 3 200 5 200 0.59 [0.14; 2.52] 1.0% 1.9%
Vimaleswaran et.al (2010) 4 1000 7 1000 0.57 [0.17; 1.95] 1.4% 2.3%
Raza et.al (2012) 24 87 35 88 0.58 [0.31; 1.09] 5.4% 4.2%
Fixed effect model 3189 3046 0.60 [0.40; 0.90] 13.5% --
Random effects model 0.56 [0.31; 0.98] -- 17.2%
Heterogeneity: I^2 = 39%, τ^2 = 0.2409, p = 0.12
ancestrycategory = Native American
Muller et.al (2003) 4 657 4 328 0.50 [0.12; 2.00] 1.1% 2.0%
Fixed effect model 657 328 0.50 [0.12; 2.00] 1.1% --
Random effects model 0.50 [0.12; 2.00] -- 2.0%
Heterogeneity: not applicable
ancestrycategory = Greater Middle Eastern
Mirzaei et.al (2009) 2 156 2 156 1.00 [0.14; 7.19] 0.6% 1.2%
Bener et.al (2015) 11 764 7 764 1.58 [0.61; 4.10] 2.4% 3.1%
Bouassida et.al (2005) 0 242 2 246 0.20 [0.01; 4.22] 0.2% 0.6%
Badii et.al (2008) 5 400 4 450 1.41 [0.38; 5.29] 1.2% 2.1%
Motavallian et.al (2013) 20 100 52 100 0.23 [0.12; 0.43] 5.5% 4.2%
Bouhaha et.al (2008) 1 84 2 261 1.56 [0.14; 17.43] 0.4% 0.9%
Meshkani et.al (2007) 1 284 6 412 0.24 [0.03; 2.00] 0.5% 1.1%
Erdogan et.al (2007) 0 91 0 50 0.0% 0.0%
Mohamed et.al (2007) 2 491 0 400 4.09 [0.20; 85.46] 0.2% 0.6%
Namvaran et.al (2011) 0 101 0 128 0.0% 0.0%
Tariq et.al (2013) 8 373 2 200 2.17 [0.46; 10.32] 0.9% 1.7%
Fixed effect model 3086 3167 0.58 [0.38; 0.89] 12.0% --
Random effects model 0.85 [0.37; 1.94] -- 15.3%
Heterogeneity: I^2 = 61%, τ^2 = 0.8303, p < 0.01
ancestrycategory = Other
Sokkar et.al (2009) 1 24 10 30 0.09 [0.01; 0.74] 0.5% 1.0%
Chistiakov et.al (2010) 20 588 36 597 0.55 [0.31; 0.96] 7.0% 4.5%
Mato et.al (2016) 0 60 0 60 0.0% 0.0%
Hegele et.al (2000) 1 179 1 332 1.86 [0.12; 29.91] 0.3% 0.7%
Avzaletdinova et.al (2016) 11 294 6 326 2.07 [0.76; 5.68] 2.1% 2.9%
Fixed effect model 1145 1345 0.69 [0.43; 1.11] 9.9% --
Random effects model 0.71 [0.23; 2.24] -- 9.1%
Heterogeneity: I^2 = 68%, τ^2 = 0.8028, p = 0.03
ancestrycategory = Other admixed ancestry
Vergotine et.al (2014) 0 212 1 575 0.90 [0.04; 22.21] 0.2% 0.5%
Fixed effect model 212 575 0.90 [0.04; 22.21] 0.2% --
Random effects model 0.90 [0.04; 22.21] -- 0.5%
Heterogeneity: not applicable
ancestrycategory = South East Asian
Paramasivam et.al (2016) 8 120 18 121 0.41 [0.17; 0.98] 2.9% 3.3%
Fixed effect model 120 121 0.41 [0.17; 0.98] 2.9% --
Random effects model 0.41 [0.17; 0.98] -- 3.3%
Heterogeneity: not applicable
ancestrycategory = Asian unspecified
Nemoto et.al (2002) (B) 0 91 1 54 0.19 [0.01; 4.87] 0.2% 0.5%
Fixed effect model 91 54 0.19 [0.01; 4.87] 0.2% --
Random effects model 0.19 [0.01; 4.87] -- 0.5%
Heterogeneity: not applicable
ancestrycategory = African American or Afro-Caribbean
Kao et.al (2003) 0 436 0 1005 0.0% 0.0%
Fixed effect model 436 1005 0.0% --
Random effects model -- 0.0%
Heterogeneity: not applicable
Fixed effect model 24491 28792 0.78 [0.67; 0.90] 100.0% --
Random effects model 0.71 [0.56; 0.90] -- 100.0%
Heterogeneity: I^2 = 45%, τ^2 = 0.2581, p < 0.01
0.01 0.1 1 10 100

F

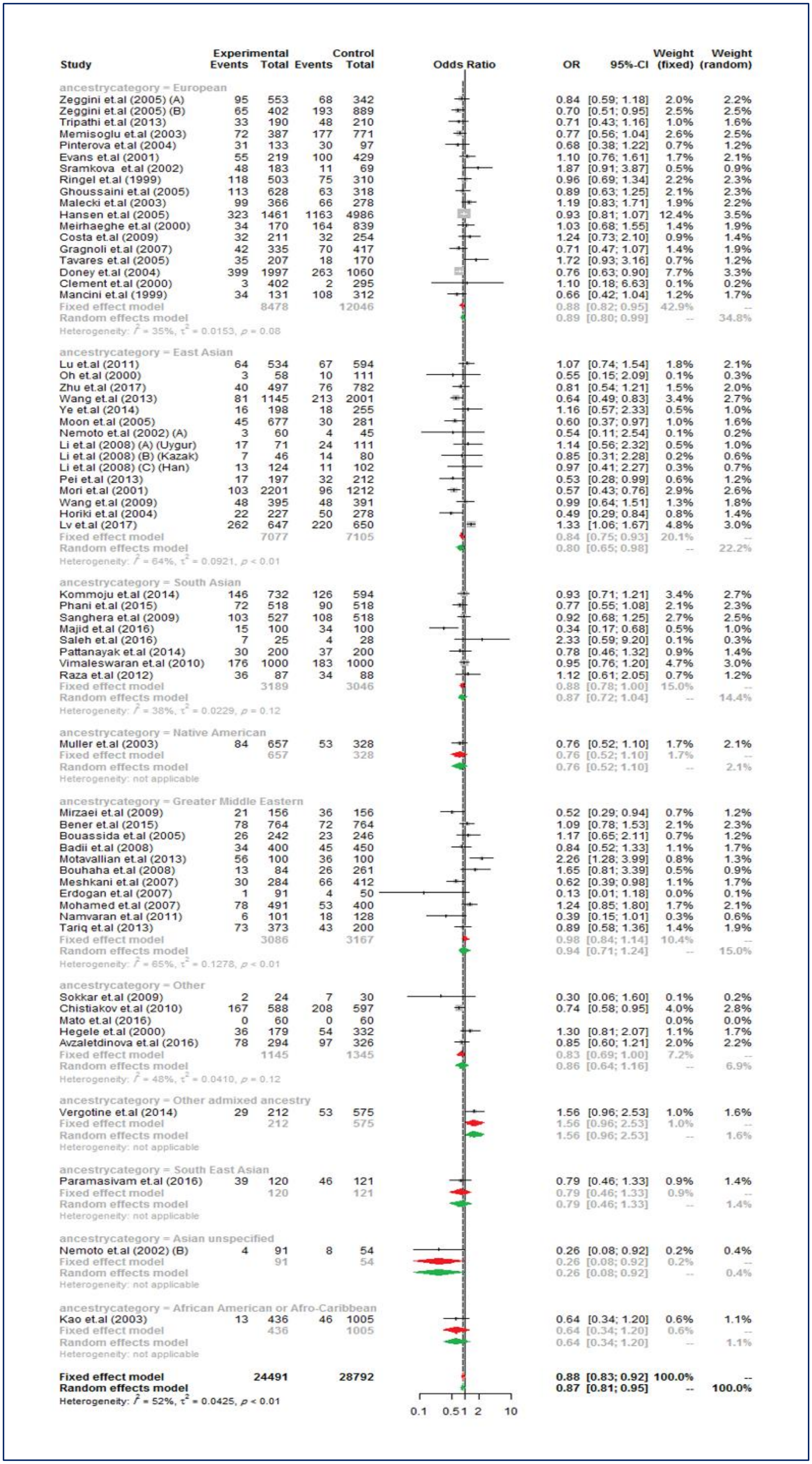
Study | Experimental Events Total | Control Events Total | Odds Ratio | OR 95%-CI | Weight (fixed) | Weight (random)
ancestrycategory = European
Zeggini et.al (2005) (A) 95 553 68 342 0.84 [0.59; 1.18] 2.0% 2.2%
Zeggini et.al (2005) (B) 65 402 193 889 0.70 [0.51; 0.95] 2.5% 2.5%
Tripathi et.al (2013) 33 190 48 210 0.71 [0.43; 1.16] 1.0% 1.6%
Memisoglu et.al (2003) 72 387 177 771 0.77 [0.56; 1.04] 2.6% 2.5%
Pinterova et.al (2004) 31 133 30 97 0.68 [0.38; 1.22] 0.7% 1.2%
Evans et.al (2001) 55 219 100 429 1.10 [0.76; 1.61] 1.7% 2.1%
Sramkova et.al (2002) 48 183 11 69 1.87 [0.91; 3.87] 0.5% 0.9%
Ringel et.al (1999) 118 503 75 310 0.96 [0.69; 1.34] 2.2% 2.3%
Ghoussaini et.al (2005) 113 628 63 318 0.89 [0.63; 1.25] 2.1% 2.3%
Malecki et.al (2003) 99 366 66 278 1.19 [0.83; 1.71] 1.9% 2.2%
Hansen et.al (2005) 323 1461 1163 4986 0.93 [0.81; 1.07] 12.4% 3.5%
Meirhaeghe et.al (2000) 34 170 164 839 1.03 [0.68; 1.55] 1.4% 1.9%
Costa et.al (2009) 32 211 32 254 1.24 [0.73; 2.10] 0.9% 1.4%
Gragnoli et.al (2007) 42 335 70 417 0.71 [0.47; 1.07] 1.4% 1.9%
Tavares et.al (2005) 35 207 18 170 1.72 [0.93; 3.16] 0.7% 1.2%
Doney et.al (2004) 399 1997 263 1060 0.76 [0.63; 0.90] 7.7% 3.3%
Clement et.al (2000) 3 402 2 295 1.10 [0.18; 6.63] 0.1% 0.2%
Mancini et.al (1999) 34 131 108 312 0.66 [0.42; 1.04] 1.2% 1.7%
Fixed effect model 8478 12046 0.88 [0.82; 0.95] 42.9% --
Random effects model 0.89 [0.80; 0.99] -- 34.8%
Heterogeneity: I^2 = 35%, τ^2 = 0.0153, p = 0.08
ancestrycategory = East Asian
Lu et.al (2011) 64 534 67 594 1.07 [0.74; 1.54] 1.8% 2.1%
Oh et.al (2000) 3 58 10 111 0.55 [0.15; 2.09] 0.1% 0.3%
Zhu et.al (2017) 40 497 76 782 0.81 [0.54; 1.21] 1.5% 2.0%
Wang et.al (2013) 81 1145 213 2001 0.64 [0.49; 0.83] 3.4% 2.7%
Ye et.al (2014) 16 198 18 255 1.16 [0.57; 2.33] 0.5% 1.0%
Moon et.al (2005) 45 677 30 281 0.60 [0.37; 0.97] 1.0% 1.6%
Nemoto et.al (2002) (A) 3 60 4 45 0.54 [0.11; 2.54] 0.1% 0.2%
Li et.al (2008) (A) (Uygur) 17 71 24 111 1.14 [0.56; 2.32] 0.5% 1.0%
Li et.al (2008) (B) (Kazak) 7 46 14 80 0.85 [0.31; 2.28] 0.2% 0.6%
Li et.al (2008) (C) (Han) 13 124 11 102 0.97 [0.41; 2.27] 0.3% 0.7%
Pei et.al (2013) 17 197 32 212 0.53 [0.28; 0.99] 0.6% 1.2%
Mori et.al (2001) 103 2201 96 1212 0.57 [0.43; 0.76] 2.9% 2.6%
Wang et.al (2009) 48 395 48 391 0.99 [0.64; 1.51] 1.3% 1.8%
Horiki et.al (2004) 22 227 50 278 0.49 [0.29; 0.84] 0.8% 1.4%
Lv et.al (2017) 262 647 220 650 1.33 [1.06; 1.67] 4.8% 3.0%
Fixed effect model 7077 7105 0.84 [0.75; 0.93] 20.1% --
Random effects model 0.80 [0.65; 0.98] -- 22.2%
Heterogeneity: I^2 = 64%, τ^2 = 0.0921, p < 0.01
ancestrycategory = South Asian
Kommoju et.al (2014) 146 732 126 594 0.93 [0.71; 1.21] 3.4% 2.7%
Phani et.al (2015) 72 518 90 518 0.77 [0.55; 1.08] 2.1% 2.3%
Sanghera et.al (2009) 103 527 108 518 0.92 [0.68; 1.25] 2.7% 2.5%
Majid et.al (2016) 15 100 34 100 0.34 [0.17; 0.68] 0.5% 1.0%
Saleh et.al (2016) 7 25 4 28 2.33 [0.59; 9.20] 0.1% 0.3%
Pattanayak et.al (2014) 30 200 37 200 0.78 [0.46; 1.32] 0.9% 1.4%
Vimaleswaran et.al (2010) 176 1000 183 1000 0.95 [0.76; 1.20] 4.7% 3.0%
Raza et.al (2012) 36 87 34 88 1.12 [0.61; 2.05] 0.7% 1.2%
Fixed effect model 3189 3046 0.88 [0.78; 1.00] 15.0% --
Random effects model 0.87 [0.72; 1.04] -- 14.4%
Heterogeneity: I^2 = 38%, τ^2 = 0.0229, p = 0.12
ancestrycategory = Native American
Muller et.al (2003) 84 657 53 328 0.76 [0.52; 1.10] 1.7% 2.1%
Fixed effect model 657 328 0.76 [0.52; 1.10] 1.7% --
Random effects model 0.76 [0.52; 1.10] -- 2.1%
Heterogeneity: not applicable
ancestrycategory = Greater Middle Eastern
Mirzaei et.al (2009) 21 156 36 156 0.52 [0.29; 0.94] 0.7% 1.2%
Bener et.al (2015) 78 764 72 764 1.09 [0.78; 1.53] 2.1% 2.3%
Bouassida et.al (2005) 26 242 23 246 1.17 [0.65; 2.11] 0.7% 1.2%
Badii et.al (2008) 34 400 45 450 0.84 [0.52; 1.33] 1.1% 1.7%
Motavallian et.al (2013) 56 100 36 100 2.26 [1.28; 3.99] 0.8% 1.3%
Bouhaha et.al (2008) 13 84 26 261 1.65 [0.81; 3.39] 0.5% 0.9%
Meshkani et.al (2007) 30 284 66 412 0.62 [0.39; 0.98] 1.1% 1.7%
Erdogan et.al (2007) 1 91 4 50 0.13 [0.01; 1.18] 0.0% 0.1%
Mohamed et.al (2007) 78 491 53 400 1.24 [0.85; 1.80] 1.7% 2.1%
Namvaran et.al (2011) 6 101 18 128 0.39 [0.15; 1.01] 0.3% 0.6%
Tariq et.al (2013) 73 373 43 200 0.89 [0.58; 1.36] 1.4% 1.9%
Fixed effect model 3086 3167 0.98 [0.84; 1.14] 10.4% --
Random effects model 0.94 [0.71; 1.24] -- 15.0%
Heterogeneity: I^2 = 65%, τ^2 = 0.1278, p < 0.01
ancestrycategory = Other
Sokkar et.al (2009) 2 24 7 30 0.30 [0.06; 1.60] 0.1% 0.2%
Chistiakov et.al (2010) 167 588 208 597 0.74 [0.58; 0.95] 4.0% 2.8%
Mato et.al (2016) 0 60 0 60 0.0% 0.0%
Hegele et.al (2000) 36 179 54 332 1.30 [0.81; 2.07] 1.1% 1.7%
Avzaletdinova et.al (2016) 78 294 97 326 0.85 [0.60; 1.21] 2.0% 2.2%
Fixed effect model 1145 1345 0.83 [0.69; 1.00] 7.2% --
Random effects model 0.86 [0.64; 1.16] -- 6.9%
Heterogeneity: I^2 = 48%, τ^2 = 0.0410, p = 0.12
ancestrycategory = Other admixed ancestry
Vergotine et.al (2014) 29 212 53 575 1.56 [0.96; 2.53] 1.0% 1.6%
Fixed effect model 212 575 1.56 [0.96; 2.53] 1.0% --
Random effects model 1.56 [0.96; 2.53] -- 1.6%
Heterogeneity: not applicable
ancestrycategory = South East Asian
Paramasivam et.al (2016) 39 120 46 121 0.79 [0.46; 1.33] 0.9% 1.4%
Fixed effect model 120 121 0.79 [0.46; 1.33] 0.9% --
Random effects model 0.79 [0.46; 1.33] -- 1.4%
Heterogeneity: not applicable
ancestrycategory = Asian unspecified
Nemoto et.al (2002) (B) 4 91 8 54 0.26 [0.08; 0.92] 0.2% 0.4%
Fixed effect model 91 54 0.26 [0.08; 0.92] 0.2% --
Random effects model 0.26 [0.08; 0.92] -- 0.4%
Heterogeneity: not applicable
ancestrycategory = African American or Afro-Caribbean
Kao et.al (2003) 13 436 46 1005 0.64 [0.34; 1.20] 0.6% 1.1%
Fixed effect model 436 1005 0.64 [0.34; 1.20] 0.6% --
Random effects model 0.64 [0.34; 1.20] -- 1.1%
Heterogeneity: not applicable
Fixed effect model 24491 28792 0.88 [0.83; 0.92] 100.0% --
Random effects model 0.87 [0.81; 0.95] -- 100.0%
Heterogeneity: I^2 = 52%, τ^2 = 0.0425, p < 0.01
0.1 0.51 2 10

G

Figure S1. Forests for Pro12Ala polymorphism (rs1801282) and T2DM risk. “A” represents allele (G vs. C); “B” represents homozygote (GG vs. CC); “C” represents heterozygote (CG vs. CC); “D” represents additive (GG vs. CG); “E” represents dominant (CG+GG vs. CC); “F” represents recessive (GG vs. CC+CG); “G” co-dominant model (CG vs. CC+GG). Areas of gray Square stand for the studies-specific weight. Red and green signs represent the pooled ORs and 95% CIs of the overall population with fixed effect and random effects model, respectively. 95% CI = 95% confidence interval, x^2_n = heterogeneity Q statistic, I^2 = I-squared metric of the heterogeneity, n = degrees of freedom; OR = odds ratio; P = P value of heterogeneity Q test.
