# Supplementary material for: PPARG (Pro12Ala) genetic variant and risk of T2DM: a systematic review and meta-analysis
Source: Sci Rep. 2020 Jul 29;10:12764. doi: 10.1038/s41598-020-69363-7 (PMC7391673; doi:10.1038/s41598-020-69363-7)
Supplement: Supplementary file 4 — Supplementary Information 4. [file 41598_2020_69363_MOESM4_ESM.pdf]

PPARG (Pro12Ala) genetic variant and risk of T2DM: a systematic review and meta-analysis

Negar Sarhangi [1], Farshad Sharifi [2], Leila Hashemian [3], Maryam Hassani Doabsari [3], Katayoun Heshmatzad [3], Marzieh Rahbaran [3], Seyed Hamid Jamaldini [3], Hamid Reza Aghaei Meybodi [1, 4], Mandana Hasanzad [1,3,*]

[1] Personalized Medicine Research Center, Endocrinology and Metabolism Clinical Sciences Institute, Tehran University of Medical Sciences, Tehran 1411413137, Iran

[2] Elderly Health Research Center, Endocrinology and Metabolism Population Sciences Institute, Tehran University of Medical Sciences, Tehran 1411413137, Iran

[3] Medical Genomics Research Center, Tehran Medical Sciences, Islamic Azad University, Tehran 1916893813, Iran

[4] Endocrinology and Metabolism Research Center, Endocrinology and Metabolism Clinical Sciences Institute, Tehran University of Medical Sciences, Tehran 1411413137, Iran

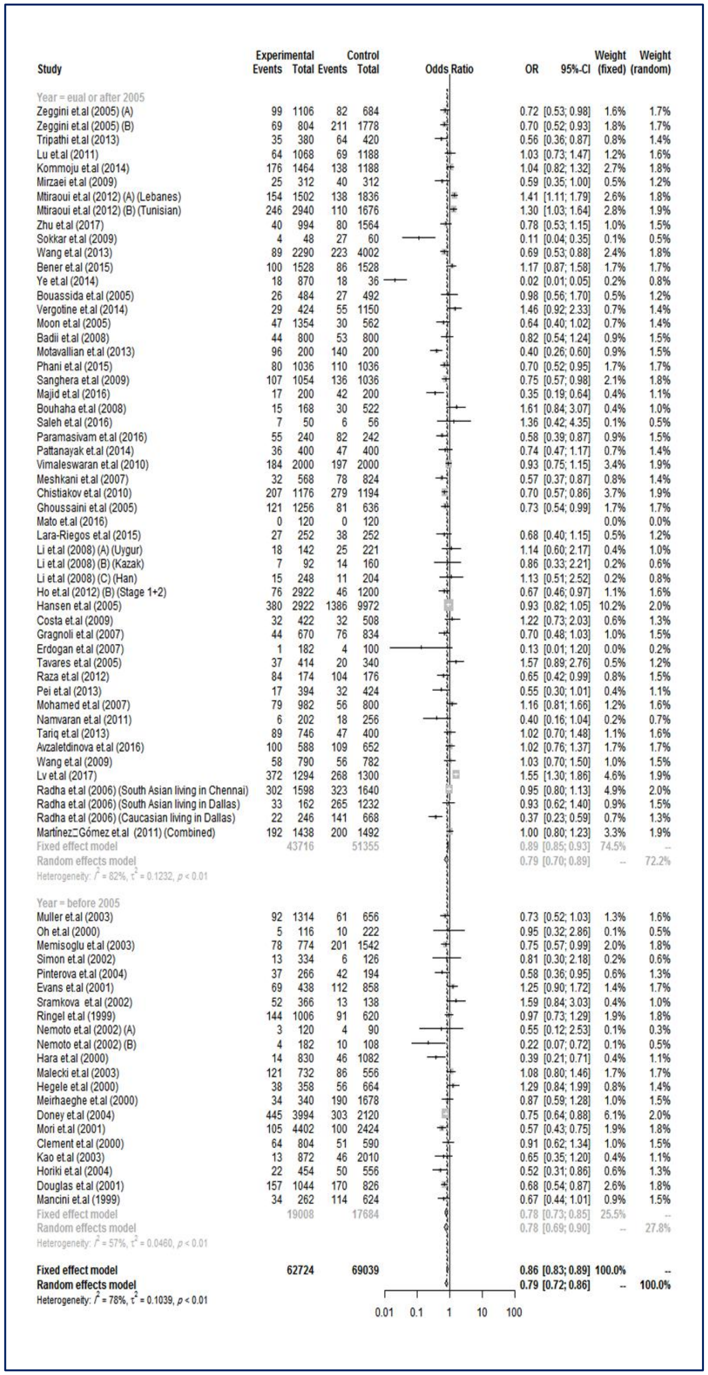
Study
Experimental Events Total
Control Events Total
Odds Ratio
OR 95%-CI
Weight (fixed)
Weight (random)
Year = eual or after 2005
Fixed effect model 43716 51355 0.89 [0.85; 0.93] 74.5%
Random effects model 0.79 [0.70; 0.89] 72.2%
Year = before 2005
Fixed effect model 19008 17684 0.78 [0.73; 0.85] 25.5%
Random effects model 0.78 [0.69; 0.90] 27.8%
Fixed effect model 62724 69039 0.86 [0.83; 0.89] 100.0%
Random effects model 0.79 [0.72; 0.86] 100.0%
0.01 0.1 1 10 100

A

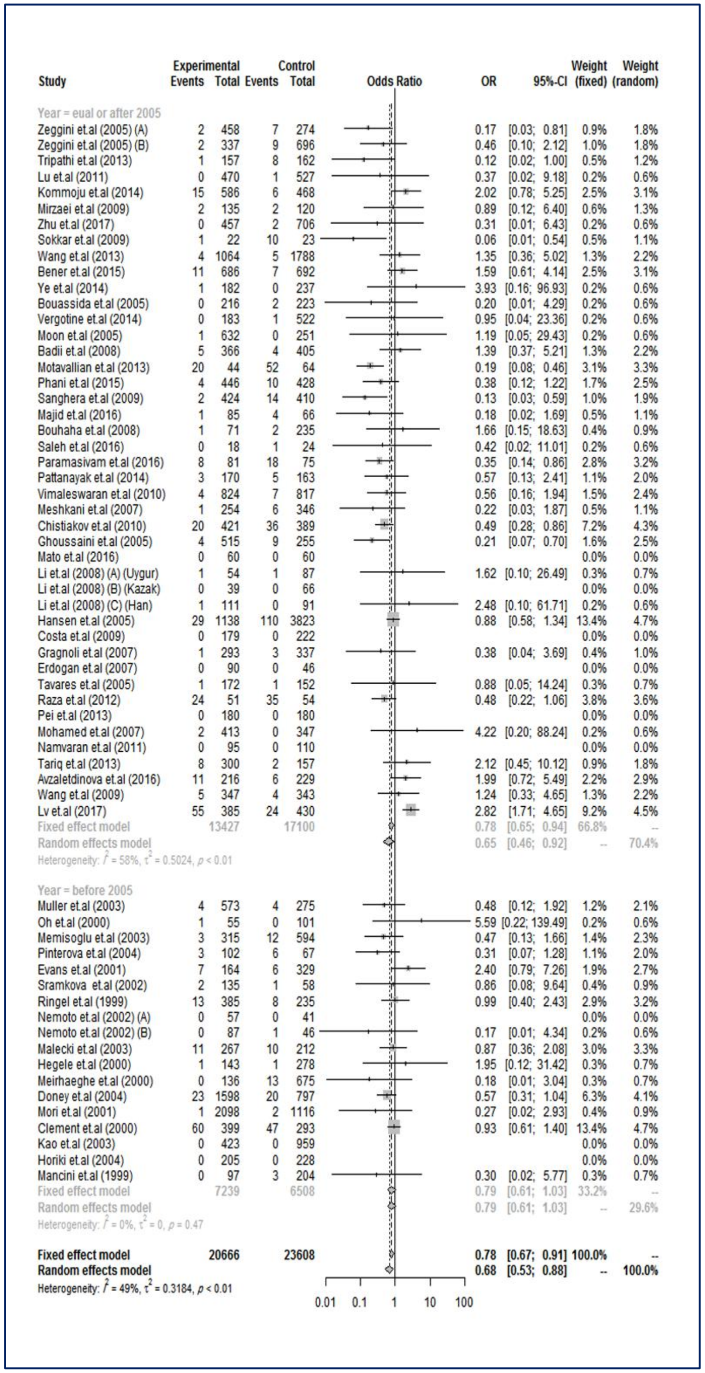
Study
Experimental Events Total
Control Events Total
Odds Ratio
OR 95%-CI
Weight (fixed)
Weight (random)
Year = eual or after 2005
Fixed effect model 13427 17100 0.78 [0.65; 0.94] 66.8%
Random effects model 0.65 [0.46; 0.92] 70.4%
Year = before 2005
Fixed effect model 7239 6508 0.79 [0.61; 1.03] 33.2%
Random effects model 0.79 [0.61; 1.03] 29.6%
Fixed effect model 20666 23608 0.78 [0.67; 0.91] 100.0%
Random effects model 0.68 [0.53; 0.88] 100.0%
0.01 0.1 1 10 100

B

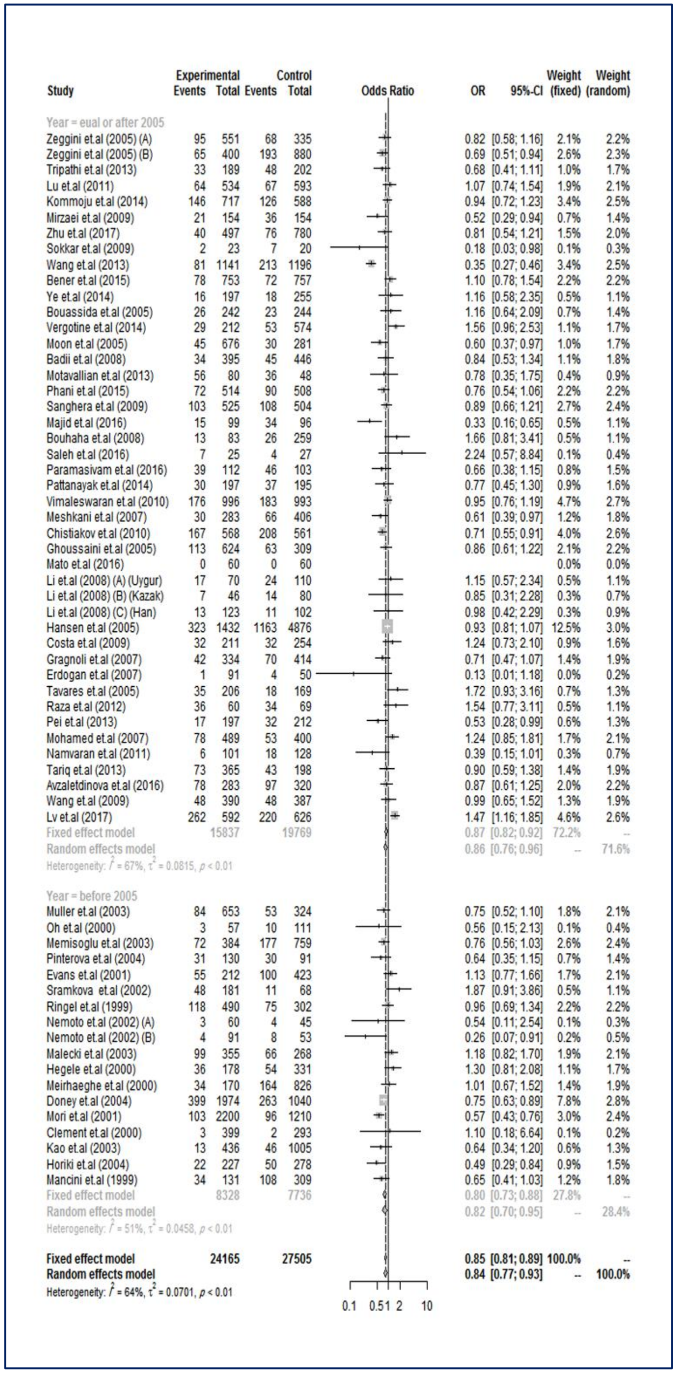
Study
Experimental Events Total
Control Events Total
Odds Ratio
OR 95%-CI
Weight (fixed)
Weight (random)
Year = eual or after 2005
Fixed effect model 15837 19769 0.87 [0.82; 0.92] 72.2%
Random effects model 0.86 [0.76; 0.96] 71.6%
Year = before 2005
Fixed effect model 8328 7736 0.80 [0.73; 0.88] 27.8%
Random effects model 0.82 [0.70; 0.95] 28.4%
Fixed effect model 24165 27505 0.85 [0.81; 0.89] 100.0%
Random effects model 0.84 [0.77; 0.93] 100.0%
0.1 0.51 2 10

C

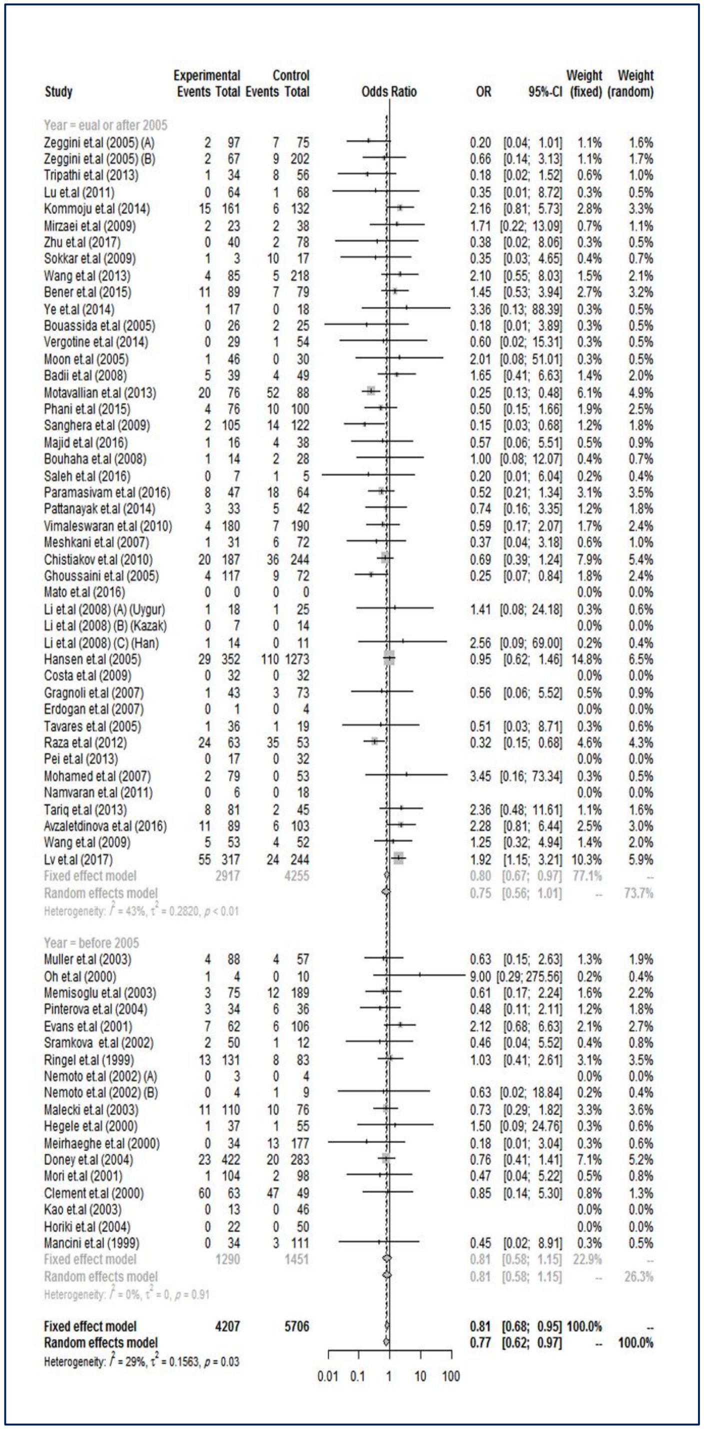
Study | Experimental Events Total | Control Events Total | Odds Ratio | OR | 95%-CI | Weight (fixed) | Weight (random)
Year = eual or after 2005
Zeggini et.al (2005) (A) 2 97 7 75 0.20 [0.04; 1.01] 1.1% 1.6%
Zeggini et.al (2005) (B) 2 67 9 202 0.66 [0.14; 3.13] 1.1% 1.7%
Tripathi et.al (2013) 1 34 8 56 0.18 [0.02; 1.52] 0.6% 1.0%
Lu et.al (2011) 0 64 1 68 0.35 [0.01; 8.72] 0.3% 0.5%
Kommoju et.al (2014) 15 161 6 132 2.16 [0.81; 5.73] 2.8% 3.3%
Mirzaei et.al (2009) 2 23 2 38 1.71 [0.22; 13.09] 0.7% 1.1%
Zhu et.al (2017) 0 40 2 78 0.38 [0.02; 8.06] 0.3% 0.5%
Sokkar et.al (2009) 1 3 10 17 0.35 [0.03; 4.65] 0.4% 0.7%
Wang et.al (2013) 4 85 5 218 2.10 [0.55; 8.03] 1.5% 2.1%
Bener et.al (2015) 11 89 7 79 1.45 [0.53; 3.94] 2.7% 3.2%
Ye et.al (2014) 1 17 0 18 3.36 [0.13; 88.39] 0.3% 0.5%
Bouassida et.al (2005) 0 26 2 25 0.18 [0.01; 3.89] 0.3% 0.5%
Vergotine et.al (2014) 0 29 1 54 0.60 [0.02; 15.31] 0.3% 0.5%
Moon et.al (2005) 1 46 0 30 2.01 [0.08; 51.01] 0.3% 0.5%
Badii et.al (2008) 5 39 4 49 1.65 [0.41; 6.63] 1.4% 2.0%
Motavallian et.al (2013) 20 76 52 88 0.25 [0.13; 0.48] 6.1% 4.9%
Phani et.al (2015) 4 76 10 100 0.50 [0.15; 1.66] 1.9% 2.5%
Sanghera et.al (2009) 2 105 14 122 0.15 [0.03; 0.68] 1.2% 1.8%
Majid et.al (2016) 1 16 4 38 0.57 [0.06; 5.51] 0.5% 0.9%
Bouhaha et.al (2008) 1 14 2 28 1.00 [0.08; 12.07] 0.4% 0.7%
Saleh et.al (2016) 0 7 1 5 0.20 [0.01; 6.04] 0.2% 0.4%
Paramasivam et.al (2016) 8 47 18 64 0.52 [0.21; 1.34] 3.1% 3.5%
Pattanayak et.al (2014) 3 33 5 42 0.74 [0.16; 3.35] 1.2% 1.8%
Vimaleswaran et.al (2010) 4 180 7 190 0.59 [0.17; 2.07] 1.7% 2.4%
Meshkani et.al (2007) 1 31 6 72 0.37 [0.04; 3.18] 0.6% 1.0%
Chistiakov et.al (2010) 20 187 36 244 0.69 [0.39; 1.24] 7.9% 5.4%
Ghoussaini et.al (2005) 4 117 9 72 0.25 [0.07; 0.84] 1.8% 2.4%
Mato et.al (2016) 0 0 0 0 0.0% 0.0%
Li et.al (2008) (A) (Uygur) 1 18 1 25 1.41 [0.08; 24.18] 0.3% 0.6%
Li et.al (2008) (B) (Kazak) 0 7 0 14 0.0% 0.0%
Li et.al (2008) (C) (Han) 1 14 0 11 2.56 [0.09; 69.00] 0.2% 0.4%
Hansen et.al (2005) 29 352 110 1273 0.95 [0.62; 1.46] 14.8% 6.5%
Costa et.al (2009) 0 32 0 32 0.0% 0.0%
Gragnoli et.al (2007) 1 43 3 73 0.56 [0.06; 5.52] 0.5% 0.9%
Erdogan et.al (2007) 0 1 0 4 0.0% 0.0%
Tavares et.al (2005) 1 36 1 19 0.51 [0.03; 8.71] 0.3% 0.6%
Raza et.al (2012) 24 63 35 53 0.32 [0.15; 0.68] 4.6% 4.3%
Pei et.al (2013) 0 17 0 32 0.0% 0.0%
Mohamed et.al (2007) 2 79 0 53 3.45 [0.16; 73.34] 0.3% 0.5%
Namvaran et.al (2011) 0 6 0 18 0.0% 0.0%
Tariq et.al (2013) 8 81 2 45 2.36 [0.48; 11.61] 1.1% 1.6%
Avzaletdinova et.al (2016) 11 89 6 103 2.28 [0.81; 6.44] 2.5% 3.0%
Wang et.al (2009) 5 53 4 52 1.25 [0.32; 4.94] 1.4% 2.0%
Lv et.al (2017) 55 317 24 244 1.92 [1.15; 3.21] 10.3% 5.9%
Fixed effect model 2917 4255 0.80 [0.67; 0.97] 77.1% --
Random effects model 0.75 [0.56; 1.01] -- 73.7%
Heterogeneity: I^2 = 43%, τ^2 = 0.2820, p < 0.01
Year = before 2005
Muller et.al (2003) 4 88 4 57 0.63 [0.15; 2.63] 1.3% 1.9%
Oh et.al (2000) 1 4 0 10 9.00 [0.29; 275.56] 0.2% 0.4%
Memisoglu et.al (2003) 3 75 12 189 0.61 [0.17; 2.24] 1.6% 2.2%
Pinterova et.al (2004) 3 34 6 36 0.48 [0.11; 2.11] 1.2% 1.8%
Evans et.al (2001) 7 62 6 106 2.12 [0.68; 6.63] 2.1% 2.7%
Sramkova et.al (2002) 2 50 1 12 0.46 [0.04; 5.52] 0.4% 0.8%
Ringel et.al (1999) 13 131 8 83 1.03 [0.41; 2.61] 3.1% 3.5%
Nemoto et.al (2002) (A) 0 3 0 4 0.0% 0.0%
Nemoto et.al (2002) (B) 0 4 1 9 0.63 [0.02; 18.84] 0.2% 0.4%
Malecki et.al (2003) 11 110 10 76 0.73 [0.29; 1.82] 3.3% 3.6%
Hegele et.al (2000) 1 37 1 55 1.50 [0.09; 24.76] 0.3% 0.6%
Meirhaeghe et.al (2000) 0 34 13 177 0.18 [0.01; 3.04] 0.3% 0.6%
Doney et.al (2004) 23 422 20 283 0.76 [0.41; 1.41] 7.1% 5.2%
Mori et.al (2001) 1 104 2 98 0.47 [0.04; 5.22] 0.5% 0.8%
Clement et.al (2000) 60 63 47 49 0.85 [0.14; 5.30] 0.8% 1.3%
Kao et.al (2003) 0 13 0 46 0.0% 0.0%
Horiki et.al (2004) 0 22 0 50 0.0% 0.0%
Mancini et.al (1999) 0 34 3 111 0.45 [0.02; 8.91] 0.3% 0.5%
Fixed effect model 1290 1451 0.81 [0.58; 1.15] 22.9% --
Random effects model 0.81 [0.58; 1.15] -- 26.3%
Heterogeneity: I^2 = 0%, τ^2 = 0, p = 0.91
Fixed effect model 4207 5706 0.81 [0.68; 0.95] 100.0% --
Random effects model 0.77 [0.62; 0.97] -- 100.0%
Heterogeneity: I^2 = 29%, τ^2 = 0.1563, p = 0.03
0.01 0.1 1 10 100

D

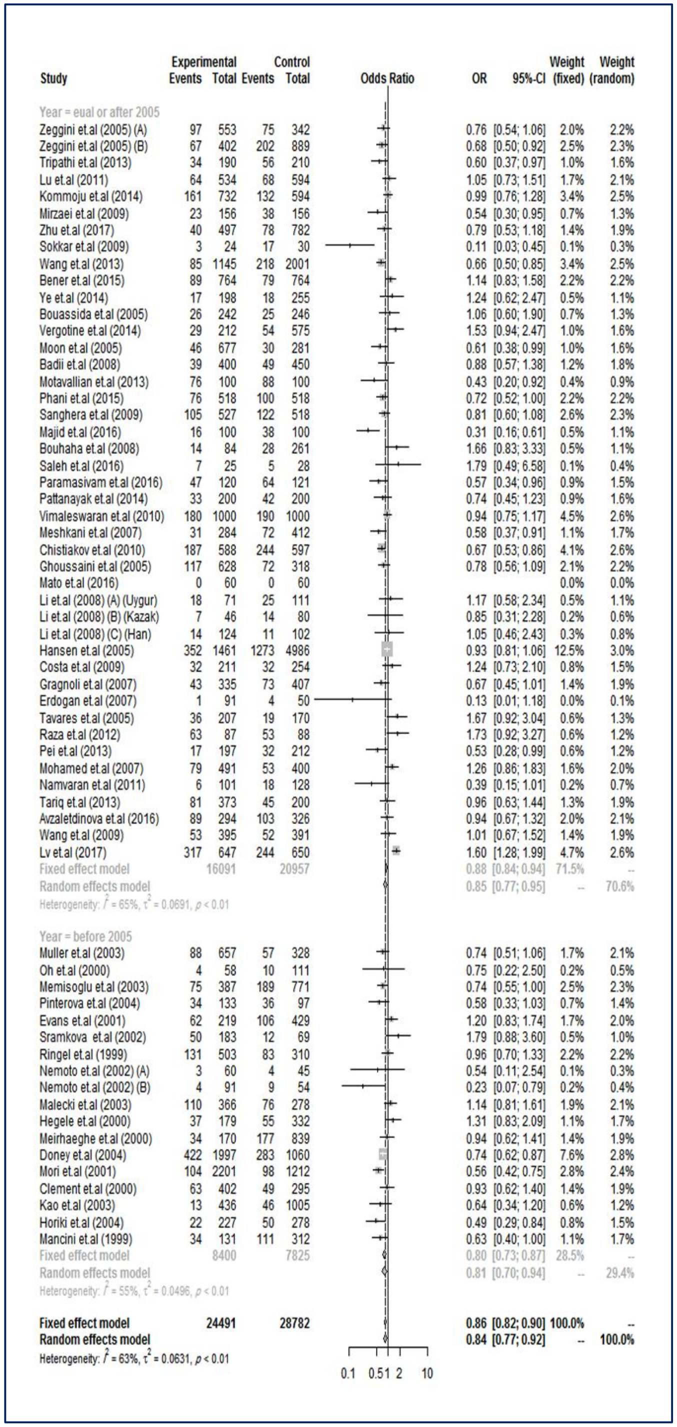
Study | Experimental Events Total | Control Events Total | Odds Ratio | OR | 95%-CI | Weight (fixed) | Weight (random)
Year = eual or after 2005
Zeggini et.al (2005) (A) 97 553 75 342 0.76 [0.54; 1.06] 2.0% 2.2%
Zeggini et.al (2005) (B) 67 402 202 889 0.68 [0.50; 0.92] 2.5% 2.3%
Tripathi et.al (2013) 34 190 56 210 0.60 [0.37; 0.97] 1.0% 1.6%
Lu et.al (2011) 64 534 68 594 1.05 [0.73; 1.51] 1.7% 2.1%
Kommoju et.al (2014) 161 732 132 594 0.99 [0.76; 1.28] 3.4% 2.5%
Mirzaei et.al (2009) 23 156 38 156 0.54 [0.30; 0.95] 0.7% 1.3%
Zhu et.al (2017) 40 497 78 782 0.79 [0.53; 1.18] 1.4% 1.9%
Sokkar et.al (2009) 3 24 17 30 0.11 [0.03; 0.45] 0.1% 0.3%
Wang et.al (2013) 85 1145 218 2001 0.66 [0.50; 0.85] 3.4% 2.5%
Bener et.al (2015) 89 764 79 764 1.14 [0.83; 1.58] 2.2% 2.2%
Ye et.al (2014) 17 198 18 255 1.24 [0.62; 2.47] 0.5% 1.1%
Bouassida et.al (2005) 26 242 25 246 1.06 [0.60; 1.90] 0.7% 1.3%
Vergotine et.al (2014) 29 212 54 575 1.53 [0.94; 2.47] 1.0% 1.6%
Moon et.al (2005) 46 677 30 281 0.61 [0.38; 0.99] 1.0% 1.6%
Badii et.al (2008) 39 400 49 450 0.88 [0.57; 1.38] 1.2% 1.8%
Motavallian et.al (2013) 76 100 88 100 0.43 [0.20; 0.92] 0.4% 0.9%
Phani et.al (2015) 76 518 100 518 0.72 [0.52; 1.00] 2.2% 2.2%
Sanghera et.al (2009) 105 527 122 518 0.81 [0.60; 1.08] 2.6% 2.3%
Majid et.al (2016) 16 100 38 100 0.31 [0.16; 0.61] 0.5% 1.1%
Bouhaha et.al (2008) 14 84 28 261 1.66 [0.83; 3.33] 0.5% 1.1%
Saleh et.al (2016) 7 25 5 28 1.79 [0.49; 6.58] 0.1% 0.4%
Paramasivam et.al (2016) 47 120 64 121 0.57 [0.34; 0.96] 0.9% 1.5%
Pattanayak et.al (2014) 33 200 42 200 0.74 [0.45; 1.23] 0.9% 1.6%
Vimaleswaran et.al (2010) 180 1000 190 1000 0.94 [0.75; 1.17] 4.5% 2.6%
Meshkani et.al (2007) 31 284 72 412 0.58 [0.37; 0.91] 1.1% 1.7%
Chistiakov et.al (2010) 187 588 244 597 0.67 [0.53; 0.86] 4.1% 2.6%
Ghoussaini et.al (2005) 117 628 72 318 0.78 [0.56; 1.09] 2.1% 2.2%
Mato et.al (2016) 0 60 0 60 0.0% 0.0%
Li et.al (2008) (A) (Uygur) 18 71 25 111 1.17 [0.58; 2.34] 0.5% 1.1%
Li et.al (2008) (B) (Kazak) 7 46 14 80 0.85 [0.31; 2.28] 0.2% 0.6%
Li et.al (2008) (C) (Han) 14 124 11 102 1.05 [0.45; 2.43] 0.3% 0.8%
Hansen et.al (2005) 352 1461 1273 4986 0.93 [0.81; 1.06] 12.5% 3.0%
Costa et.al (2009) 32 211 32 254 1.24 [0.73; 2.10] 0.8% 1.5%
Gragnoli et.al (2007) 43 335 73 407 0.67 [0.45; 1.01] 1.4% 1.9%
Erdogan et.al (2007) 1 91 4 50 0.13 [0.01; 1.18] 0.0% 0.1%
Tavares et.al (2005) 36 207 19 170 1.67 [0.92; 3.04] 0.6% 1.3%
Raza et.al (2012) 63 87 53 88 1.73 [0.92; 3.27] 0.6% 1.2%
Pei et.al (2013) 17 197 32 212 0.53 [0.28; 0.99] 0.6% 1.2%
Mohamed et.al (2007) 79 491 53 400 1.26 [0.86; 1.83] 1.6% 2.0%
Namvaran et.al (2011) 6 101 18 128 0.39 [0.15; 1.01] 0.2% 0.7%
Tariq et.al (2013) 81 373 45 200 0.96 [0.63; 1.44] 1.3% 1.9%
Avzaletdinova et.al (2016) 89 294 103 326 0.94 [0.67; 1.32] 2.0% 2.1%
Wang et.al (2009) 53 395 52 391 1.01 [0.67; 1.52] 1.4% 1.9%
Lv et.al (2017) 317 647 244 650 1.60 [1.28; 1.99] 4.7% 2.6%
Fixed effect model 16091 20957 0.88 [0.84; 0.94] 71.5% --
Random effects model 0.85 [0.77; 0.95] -- 70.6%
Heterogeneity: I^2 = 65%, τ^2 = 0.0691, p < 0.01
Year = before 2005
Muller et.al (2003) 88 657 57 328 0.74 [0.51; 1.06] 1.7% 2.1%
Oh et.al (2000) 4 58 10 111 0.75 [0.22; 2.50] 0.2% 0.5%
Memisoglu et.al (2003) 75 387 189 771 0.74 [0.55; 1.00] 2.5% 2.3%
Pinterova et.al (2004) 34 133 36 97 0.58 [0.33; 1.03] 0.7% 1.4%
Evans et.al (2001) 62 219 106 429 1.20 [0.83; 1.74] 1.7% 2.0%
Sramkova et.al (2002) 50 183 12 69 1.79 [0.88; 3.60] 0.5% 1.0%
Ringel et.al (1999) 131 503 83 310 0.96 [0.70; 1.33] 2.2% 2.2%
Nemoto et.al (2002) (A) 3 60 4 45 0.54 [0.11; 2.54] 0.1% 0.3%
Nemoto et.al (2002) (B) 4 91 9 54 0.23 [0.07; 0.79] 0.2% 0.4%
Malecki et.al (2003) 110 366 76 278 1.14 [0.81; 1.61] 1.9% 2.1%
Hegele et.al (2000) 37 179 55 332 1.31 [0.83; 2.09] 1.1% 1.7%
Meirhaeghe et.al (2000) 34 170 177 839 0.94 [0.62; 1.41] 1.4% 1.9%
Doney et.al (2004) 422 1997 283 1060 0.74 [0.62; 0.87] 7.6% 2.8%
Mori et.al (2001) 104 2201 98 1212 0.56 [0.42; 0.75] 2.8% 2.4%
Clement et.al (2000) 63 402 49 295 0.93 [0.62; 1.40] 1.4% 1.9%
Kao et.al (2003) 13 436 46 1005 0.64 [0.34; 1.20] 0.6% 1.2%
Horiki et.al (2004) 22 227 50 278 0.49 [0.29; 0.84] 0.8% 1.5%
Mancini et.al (1999) 34 131 111 312 0.63 [0.40; 1.00] 1.1% 1.7%
Fixed effect model 8400 7825 0.80 [0.73; 0.87] 28.5% --
Random effects model 0.81 [0.70; 0.94] -- 29.4%
Heterogeneity: I^2 = 55%, τ^2 = 0.0496, p < 0.01
Fixed effect model 24491 28782 0.86 [0.82; 0.90] 100.0% --
Random effects model 0.84 [0.77; 0.92] -- 100.0%
Heterogeneity: I^2 = 63%, τ^2 = 0.0631, p < 0.01
0.1 0.51 2 10

E

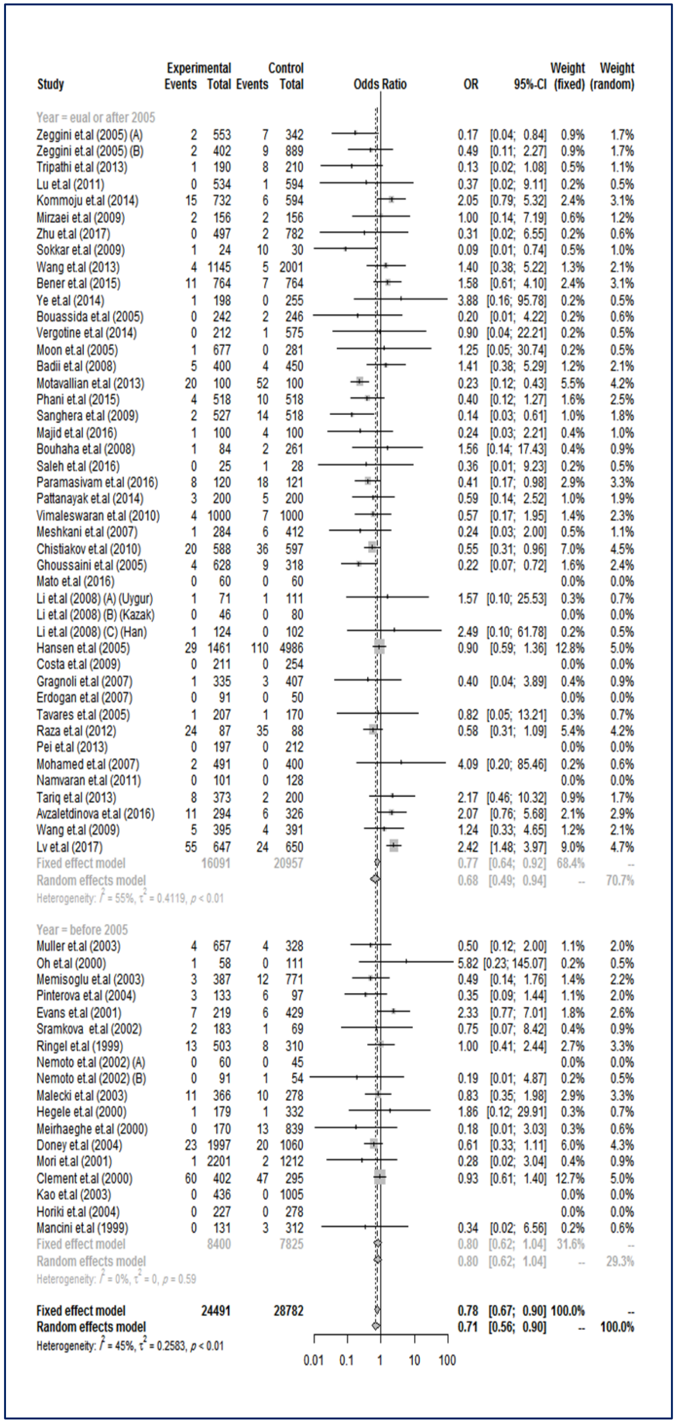
Study | Experimental Events Total | Control Events Total | Odds Ratio | OR | 95%-CI | Weight (fixed) | Weight (random)
Year = eual or after 2005
Zeggini et.al (2005) (A) 2 553 7 342 0.17 [0.04; 0.84] 0.9% 1.7%
Zeggini et.al (2005) (B) 2 402 9 889 0.49 [0.11; 2.27] 0.9% 1.7%
Tripathi et.al (2013) 1 190 8 210 0.13 [0.02; 1.08] 0.5% 1.1%
Lu et.al (2011) 0 534 1 594 0.37 [0.02; 9.11] 0.2% 0.5%
Kommoju et.al (2014) 15 732 6 594 2.05 [0.79; 5.32] 2.4% 3.1%
Mirzaei et.al (2009) 2 156 2 156 1.00 [0.14; 7.19] 0.6% 1.2%
Zhu et.al (2017) 0 497 2 782 0.31 [0.02; 6.55] 0.2% 0.6%
Sokkar et.al (2009) 1 24 10 30 0.09 [0.01; 0.74] 0.5% 1.0%
Wang et.al (2013) 4 1145 5 2001 1.40 [0.38; 5.22] 1.3% 2.1%
Bener et.al (2015) 11 764 7 764 1.58 [0.61; 4.10] 2.4% 3.1%
Ye et.al (2014) 1 198 0 255 3.88 [0.16; 95.78] 0.2% 0.5%
Bouassida et.al (2005) 0 242 2 246 0.20 [0.01; 4.22] 0.2% 0.6%
Vergotine et.al (2014) 0 212 1 575 0.90 [0.04; 22.21] 0.2% 0.5%
Moon et.al (2005) 1 677 0 281 1.25 [0.05; 30.74] 0.2% 0.5%
Badii et.al (2008) 5 400 4 450 1.41 [0.38; 5.29] 1.2% 2.1%
Motavallian et.al (2013) 20 100 52 100 0.23 [0.12; 0.43] 5.5% 4.2%
Phani et.al (2015) 4 518 10 518 0.40 [0.12; 1.27] 1.6% 2.5%
Sanghera et.al (2009) 2 527 14 518 0.14 [0.03; 0.61] 1.0% 1.8%
Majid et.al (2016) 1 100 4 100 0.24 [0.03; 2.21] 0.4% 1.0%
Bouhaha et.al (2008) 1 84 2 261 1.56 [0.14; 17.43] 0.4% 0.9%
Saleh et.al (2016) 0 25 1 28 0.36 [0.01; 9.23] 0.2% 0.5%
Paramasivam et.al (2016) 8 120 18 121 0.41 [0.17; 0.98] 2.9% 3.3%
Pattanayak et.al (2014) 3 200 5 200 0.59 [0.14; 2.52] 1.0% 1.9%
Vimaleswaran et.al (2010) 4 1000 7 1000 0.57 [0.17; 1.95] 1.4% 2.3%
Meshkani et.al (2007) 1 284 6 412 0.24 [0.03; 2.00] 0.5% 1.1%
Chistiakov et.al (2010) 20 588 36 597 0.55 [0.31; 0.96] 7.0% 4.5%
Ghoussaini et.al (2005) 4 628 9 318 0.22 [0.07; 0.72] 1.6% 2.4%
Mato et.al (2016) 0 60 0 60 0.0% 0.0%
Li et.al (2008) (A) (Uygur) 1 71 1 111 1.57 [0.10; 25.53] 0.3% 0.7%
Li et.al (2008) (B) (Kazak) 0 46 0 80 0.0% 0.0%
Li et.al (2008) (C) (Han) 1 124 0 102 2.49 [0.10; 61.78] 0.2% 0.5%
Hansen et.al (2005) 29 1461 110 4986 0.90 [0.59; 1.36] 12.8% 5.0%
Costa et.al (2009) 0 211 0 254 0.0% 0.0%
Gragnoli et.al (2007) 1 335 3 407 0.40 [0.04; 3.89] 0.4% 0.9%
Erdogan et.al (2007) 0 91 0 50 0.0% 0.0%
Tavares et.al (2005) 1 207 1 170 0.82 [0.05; 13.21] 0.3% 0.7%
Raza et.al (2012) 24 87 35 88 0.58 [0.31; 1.09] 5.4% 4.2%
Pei et.al (2013) 0 197 0 212 0.0% 0.0%
Mohamed et.al (2007) 2 491 0 400 4.09 [0.20; 85.46] 0.2% 0.6%
Namvaran et.al (2011) 0 101 0 128 0.0% 0.0%
Tariq et.al (2013) 8 373 2 200 2.17 [0.46; 10.32] 0.9% 1.7%
Avzaletdinova et.al (2016) 11 294 6 326 2.07 [0.76; 5.68] 2.1% 2.9%
Wang et.al (2009) 5 395 4 391 1.24 [0.33; 4.65] 1.2% 2.1%
Lv et.al (2017) 55 647 24 650 2.42 [1.48; 3.97] 9.0% 4.7%
Fixed effect model 16091 20957 0.77 [0.64; 0.92] 68.4% --
Random effects model 0.68 [0.49; 0.94] -- 70.7%
Heterogeneity: I^2 = 55%, τ^2 = 0.4119, p < 0.01
Year = before 2005
Muller et.al (2003) 4 657 4 328 0.50 [0.12; 2.00] 1.1% 2.0%
Oh et.al (2000) 1 58 0 111 5.82 [0.23; 145.07] 0.2% 0.5%
Memisoglu et.al (2003) 3 387 12 771 0.49 [0.14; 1.76] 1.4% 2.2%
Pinterova et.al (2004) 3 133 6 97 0.35 [0.09; 1.44] 1.1% 2.0%
Evans et.al (2001) 7 219 6 429 2.33 [0.77; 7.01] 1.8% 2.6%
Sramkova et.al (2002) 2 183 1 69 0.75 [0.07; 8.42] 0.4% 0.9%
Ringel et.al (1999) 13 503 8 310 1.00 [0.41; 2.44] 2.7% 3.3%
Nemoto et.al (2002) (A) 0 60 0 45 0.0% 0.0%
Nemoto et.al (2002) (B) 0 91 1 54 0.19 [0.01; 4.87] 0.2% 0.5%
Malecki et.al (2003) 11 366 10 278 0.83 [0.35; 1.98] 2.9% 3.3%
Hegele et.al (2000) 1 179 1 332 1.86 [0.12; 29.91] 0.3% 0.7%
Meirhaeghe et.al (2000) 0 170 13 839 0.18 [0.01; 3.03] 0.3% 0.6%
Doney et.al (2004) 23 1997 20 1060 0.61 [0.33; 1.11] 6.0% 4.3%
Mori et.al (2001) 1 2201 2 1212 0.28 [0.02; 3.04] 0.4% 0.9%
Clement et.al (2000) 60 402 47 295 0.93 [0.61; 1.40] 12.7% 5.0%
Kao et.al (2003) 0 436 0 1005 0.0% 0.0%
Horiki et.al (2004) 0 227 0 278 0.0% 0.0%
Mancini et.al (1999) 0 131 3 312 0.34 [0.02; 6.56] 0.2% 0.6%
Fixed effect model 8400 7825 0.80 [0.62; 1.04] 31.6% --
Random effects model 0.80 [0.62; 1.04] -- 29.3%
Heterogeneity: I^2 = 0%, τ^2 = 0, p = 0.59
Fixed effect model 24491 28782 0.78 [0.67; 0.90] 100.0% --
Random effects model 0.71 [0.56; 0.90] -- 100.0%
Heterogeneity: I^2 = 45%, τ^2 = 0.2583, p < 0.01
0.01 0.1 1 10 100

F

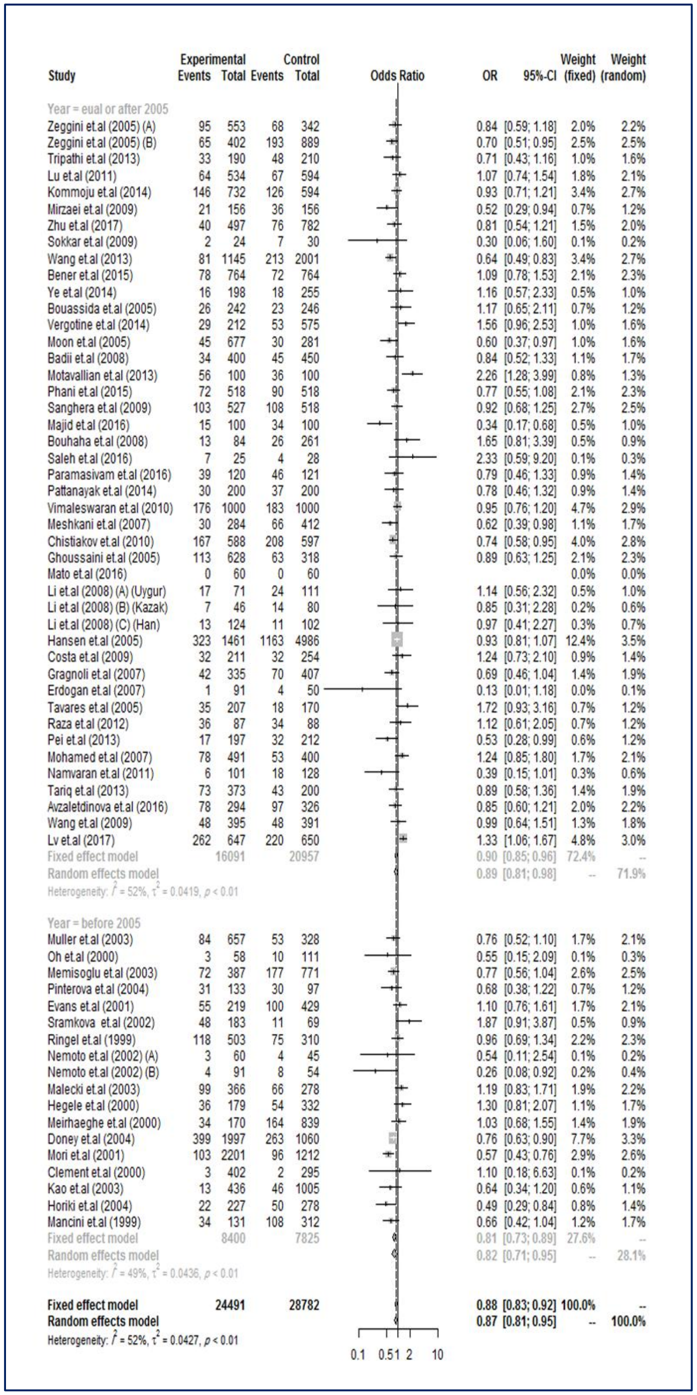

G

Figure S4. Subgroup analysis according to publication year. “A” represents allele (G vs. C); “B” represents homozygote (GG vs. CC); “C” represents heterozygote (CG vs. CC); “D” represents additive (GG vs. CG); “E” represents dominant (CG/GG vs. CC); “F” represents recessive (GG vs. CC/CG); “G” co-dominant model (CG vs. CC+GG). Vertical and horizontal lines represent ORs and the corresponding 95% CIs of each study. 95% CI = 95% confidence interval, OR = odds ratio.
