# Supplementary material for: PPARG (Pro12Ala) genetic variant and risk of T2DM: a systematic review and meta-analysis
Source: Sci Rep. 2020 Jul 29;10:12764. doi: 10.1038/s41598-020-69363-7 (PMC7391673; doi:10.1038/s41598-020-69363-7)
Supplement: Supplementary file 5 — Supplementary Information 5. [file 41598_2020_69363_MOESM5_ESM.pdf]

PPARG (Pro12Ala) genetic variant and risk of T2DM: a systematic review and meta-analysis

Negar Sarhangi [1], Farshad Sharifi [2], Leila Hashemian [3], Maryam Hassani Doabsari [3], Katayoun Heshmatzad [3], Marzieh Rahbaran [3], Seyed Hamid Jamaldini [3], Hamid Reza Aghaei Meybodi [1, 4], Mandana Hasanzad [1,3,*]

[1] Personalized Medicine Research Center, Endocrinology and Metabolism Clinical Sciences Institute, Tehran University of Medical Sciences, Tehran 1411413137, Iran

[2] Elderly Health Research Center, Endocrinology and Metabolism Population Sciences Institute, Tehran University of Medical Sciences, Tehran 1411413137, Iran

[3] Medical Genomics Research Center, Tehran Medical Sciences, Islamic Azad University, Tehran 1916893813, Iran

[4] Endocrinology and Metabolism Research Center, Endocrinology and Metabolism Clinical Sciences Institute, Tehran University of Medical Sciences, Tehran 1411413137, Iran

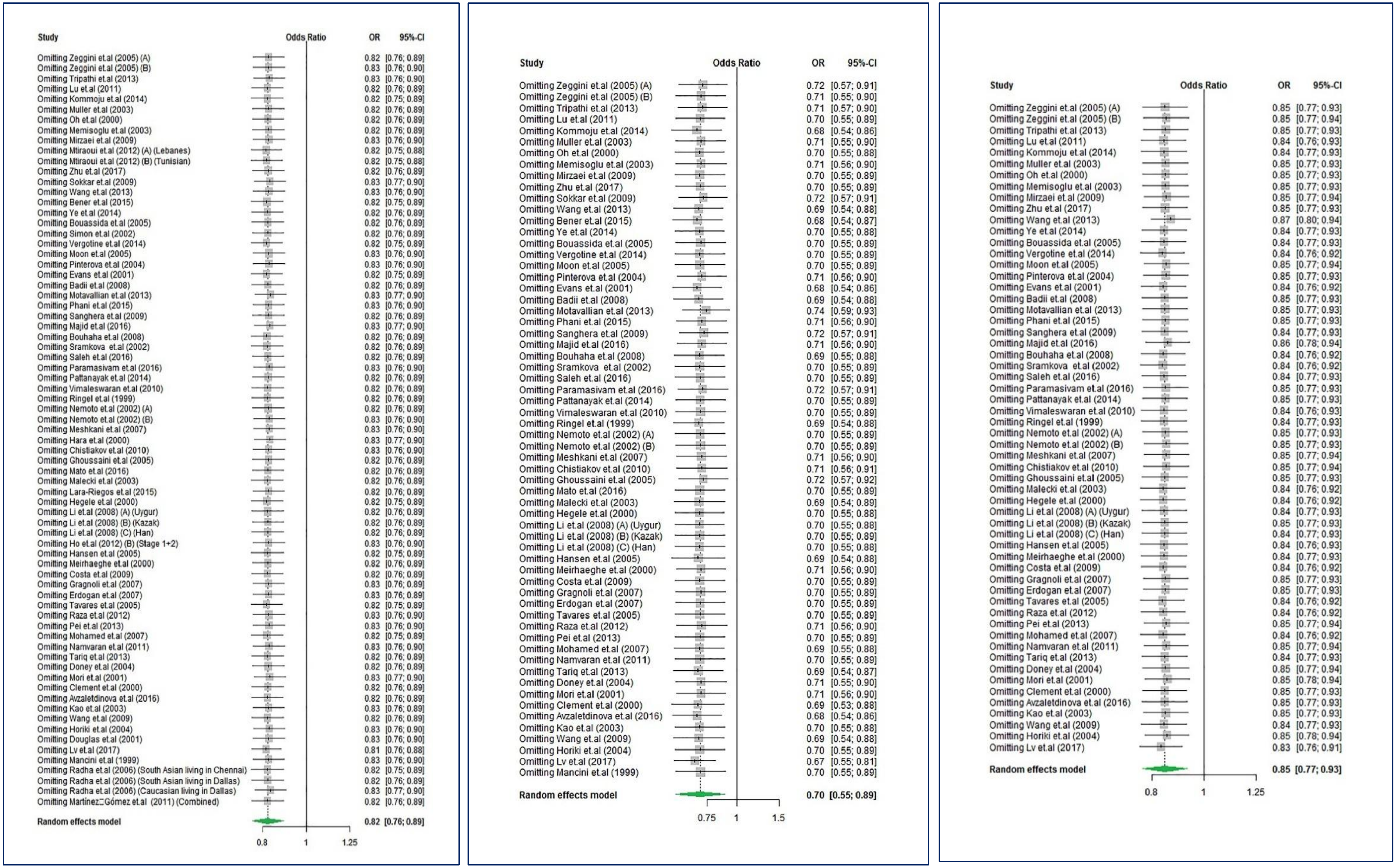
Study
Odds Ratio
OR
95%-CI
Omitting Zeggini et.al (2005) (A) 0.82 [0.76; 0.89]
Omitting Zeggini et.al (2005) (B) 0.83 [0.76; 0.90]
Omitting Tripathi et.al (2013) 0.83 [0.76; 0.90]
Omitting Lu et.al (2011) 0.82 [0.76; 0.89]
Omitting Kommoju et.al (2014) 0.82 [0.75; 0.89]
Omitting Muller et.al (2003) 0.82 [0.76; 0.89]
Omitting Oh et.al (2000) 0.82 [0.76; 0.89]
Omitting Memisoglu et.al (2003) 0.82 [0.76; 0.89]
Omitting Mirzaei et.al (2009) 0.83 [0.76; 0.90]
Omitting Mtiraoui et.al (2012) (A) (Lebanes) 0.82 [0.75; 0.88]
Omitting Mtiraoui et.al (2012) (B) (Tunisian) 0.82 [0.75; 0.88]
Omitting Zhu et.al (2017) 0.82 [0.76; 0.89]
Omitting Sokkar et.al (2009) 0.83 [0.77; 0.90]
Omitting Wang et.al (2013) 0.83 [0.76; 0.90]
Omitting Bener et.al (2015) 0.82 [0.75; 0.89]
Omitting Ye et.al (2014) 0.82 [0.76; 0.89]
Omitting Bouassida et.al (2005) 0.82 [0.76; 0.89]
Omitting Simon et.al (2002) 0.82 [0.76; 0.89]
Omitting Vergotine et.al (2014) 0.82 [0.75; 0.89]
Omitting Moon et.al (2005) 0.83 [0.76; 0.90]
Omitting Pinterova et.al (2004) 0.83 [0.76; 0.90]
Omitting Evans et.al (2001) 0.82 [0.75; 0.89]
Omitting Badii et.al (2008) 0.82 [0.76; 0.89]
Omitting Motavallian et.al (2013) 0.83 [0.77; 0.90]
Omitting Phani et.al (2015) 0.83 [0.76; 0.90]
Omitting Sanghera et.al (2009) 0.82 [0.76; 0.89]
Omitting Majid et.al (2016) 0.83 [0.77; 0.90]
Omitting Bouhaha et.al (2008) 0.82 [0.76; 0.89]
Omitting Sramkova et.al (2002) 0.82 [0.76; 0.89]
Omitting Saleh et.al (2016) 0.82 [0.76; 0.89]
Omitting Paramasivam et.al (2016) 0.83 [0.76; 0.90]
Omitting Pattanayak et.al (2014) 0.82 [0.76; 0.89]
Omitting Vimaleswaran et.al (2010) 0.82 [0.76; 0.89]
Omitting Ringel et.al (1999) 0.82 [0.76; 0.89]
Omitting Nemoto et.al (2002) (A) 0.82 [0.76; 0.89]
Omitting Nemoto et.al (2002) (B) 0.83 [0.76; 0.90]
Omitting Meshkani et.al (2007) 0.83 [0.76; 0.90]
Omitting Hara et.al (2000) 0.83 [0.77; 0.90]
Omitting Chistiakov et.al (2010) 0.83 [0.76; 0.90]
Omitting Ghoussaini et.al (2005) 0.82 [0.76; 0.89]
Omitting Mato et.al (2016) 0.82 [0.76; 0.89]
Omitting Malecki et.al (2003) 0.82 [0.76; 0.89]
Omitting Lara-Riegos et.al (2015) 0.82 [0.76; 0.89]
Omitting Hegele et.al (2000) 0.82 [0.75; 0.89]
Omitting Li et.al (2008) (A) (Uygur) 0.82 [0.76; 0.89]
Omitting Li et.al (2008) (B) (Kazak) 0.82 [0.76; 0.89]
Omitting Li et.al (2008) (C) (Han) 0.82 [0.76; 0.89]
Omitting Ho et.al (2012) (B) (Stage 1+2) 0.83 [0.76; 0.90]
Omitting Hansen et.al (2005) 0.82 [0.75; 0.89]
Omitting Meirhaeghe et.al (2000) 0.82 [0.76; 0.89]
Omitting Costa et.al (2009) 0.82 [0.76; 0.89]
Omitting Gragnoli et.al (2007) 0.83 [0.76; 0.89]
Omitting Erdogan et.al (2007) 0.83 [0.76; 0.89]
Omitting Tavares et.al (2005) 0.82 [0.75; 0.89]
Omitting Raza et.al (2012) 0.83 [0.76; 0.90]
Omitting Pei et.al (2013) 0.83 [0.76; 0.90]
Omitting Mohamed et.al (2007) 0.82 [0.75; 0.89]
Omitting Namvaran et.al (2011) 0.83 [0.76; 0.90]
Omitting Tariq et.al (2013) 0.82 [0.76; 0.89]
Omitting Doney et.al (2004) 0.82 [0.76; 0.89]
Omitting Mori et.al (2001) 0.83 [0.77; 0.90]
Omitting Clement et.al (2000) 0.82 [0.76; 0.89]
Omitting Avzaletdinova et.al (2016) 0.82 [0.76; 0.89]
Omitting Kao et.al (2003) 0.83 [0.76; 0.89]
Omitting Wang et.al (2009) 0.82 [0.76; 0.89]
Omitting Horiki et.al (2004) 0.83 [0.76; 0.90]
Omitting Douglas et.al (2001) 0.83 [0.76; 0.90]
Omitting Lv et.al (2017) 0.81 [0.76; 0.88]
Omitting Mancini et.al (1999) 0.83 [0.76; 0.90]
Omitting Radha et.al (2006) (South Asian living in Chennai) 0.82 [0.75; 0.89]
Omitting Radha et.al (2006) (South Asian living in Dallas) 0.82 [0.76; 0.89]
Omitting Radha et.al (2006) (Caucasian living in Dallas) 0.83 [0.77; 0.90]
Omitting Martínez Gómez et.al (2011) (Combined) 0.82 [0.76; 0.89]
Random effects model 0.82 [0.76; 0.89]
0.8
1
1.25
Study
Odds Ratio
OR
95%-CI
Omitting Zeggini et.al (2005) (A) 0.72 [0.57; 0.91]
Omitting Zeggini et.al (2005) (B) 0.71 [0.55; 0.90]
Omitting Tripathi et.al (2013) 0.71 [0.57; 0.90]
Omitting Lu et.al (2011) 0.70 [0.55; 0.89]
Omitting Kommoju et.al (2014) 0.68 [0.54; 0.86]
Omitting Muller et.al (2003) 0.71 [0.55; 0.90]
Omitting Oh et.al (2000) 0.70 [0.55; 0.88]
Omitting Memisoglu et.al (2003) 0.71 [0.56; 0.90]
Omitting Mirzaei et.al (2009) 0.70 [0.55; 0.89]
Omitting Zhu et.al (2017) 0.70 [0.55; 0.89]
Omitting Sokkar et.al (2009) 0.72 [0.57; 0.91]
Omitting Wang et.al (2013) 0.69 [0.54; 0.88]
Omitting Bener et.al (2015) 0.68 [0.54; 0.87]
Omitting Ye et.al (2014) 0.70 [0.55; 0.88]
Omitting Bouassida et.al (2005) 0.70 [0.55; 0.89]
Omitting Vergotine et.al (2014) 0.70 [0.55; 0.89]
Omitting Moon et.al (2005) 0.70 [0.55; 0.89]
Omitting Pinterova et.al (2004) 0.71 [0.56; 0.90]
Omitting Evans et.al (2001) 0.68 [0.54; 0.86]
Omitting Badii et.al (2008) 0.69 [0.54; 0.88]
Omitting Motavallian et.al (2013) 0.74 [0.59; 0.93]
Omitting Phani et.al (2015) 0.71 [0.56; 0.90]
Omitting Sanghera et.al (2009) 0.72 [0.57; 0.91]
Omitting Majid et.al (2016) 0.71 [0.56; 0.90]
Omitting Bouhaha et.al (2008) 0.69 [0.55; 0.88]
Omitting Sramkova et.al (2002) 0.70 [0.55; 0.89]
Omitting Saleh et.al (2016) 0.70 [0.55; 0.89]
Omitting Paramasivam et.al (2016) 0.72 [0.57; 0.91]
Omitting Pattanayak et.al (2014) 0.70 [0.55; 0.89]
Omitting Vimaleswaran et.al (2010) 0.70 [0.55; 0.89]
Omitting Ringel et.al (1999) 0.69 [0.54; 0.88]
Omitting Nemoto et.al (2002) (A) 0.70 [0.55; 0.89]
Omitting Nemoto et.al (2002) (B) 0.70 [0.55; 0.89]
Omitting Meshkani et.al (2007) 0.71 [0.56; 0.90]
Omitting Chistiakov et.al (2010) 0.71 [0.56; 0.91]
Omitting Ghoussaini et.al (2005) 0.72 [0.57; 0.92]
Omitting Mato et.al (2016) 0.70 [0.55; 0.89]
Omitting Malecki et.al (2003) 0.69 [0.54; 0.89]
Omitting Hegele et.al (2000) 0.70 [0.55; 0.88]
Omitting Li et.al (2008) (A) (Uygur) 0.70 [0.55; 0.88]
Omitting Li et.al (2008) (B) (Kazak) 0.70 [0.55; 0.89]
Omitting Li et.al (2008) (C) (Han) 0.70 [0.55; 0.88]
Omitting Hansen et.al (2005) 0.69 [0.54; 0.88]
Omitting Meirhaeghe et.al (2000) 0.71 [0.56; 0.90]
Omitting Costa et.al (2009) 0.70 [0.55; 0.89]
Omitting Gragnoli et.al (2007) 0.70 [0.55; 0.89]
Omitting Erdogan et.al (2007) 0.70 [0.55; 0.89]
Omitting Tavares et.al (2005) 0.70 [0.55; 0.89]
Omitting Raza et.al (2012) 0.71 [0.56; 0.90]
Omitting Pei et.al (2013) 0.70 [0.55; 0.89]
Omitting Mohamed et.al (2007) 0.69 [0.55; 0.88]
Omitting Namvaran et.al (2011) 0.70 [0.55; 0.89]
Omitting Tariq et.al (2013) 0.69 [0.54; 0.87]
Omitting Doney et.al (2004) 0.71 [0.55; 0.90]
Omitting Mori et.al (2001) 0.71 [0.56; 0.90]
Omitting Clement et.al (2000) 0.69 [0.53; 0.88]
Omitting Avzaletdinova et.al (2016) 0.68 [0.54; 0.86]
Omitting Kao et.al (2003) 0.70 [0.55; 0.88]
Omitting Wang et.al (2009) 0.69 [0.54; 0.88]
Omitting Horiki et.al (2004) 0.70 [0.55; 0.89]
Omitting Lv et.al (2017) 0.67 [0.55; 0.81]
Omitting Mancini et.al (1999) 0.70 [0.55; 0.89]
Random effects model 0.70 [0.55; 0.89]
0.75
1
1.5
Study
Odds Ratio
OR
95%-CI
Omitting Zeggini et.al (2005) (A) 0.85 [0.77; 0.93]
Omitting Zeggini et.al (2005) (B) 0.85 [0.77; 0.94]
Omitting Tripathi et.al (2013) 0.85 [0.77; 0.93]
Omitting Lu et.al (2011) 0.84 [0.76; 0.93]
Omitting Kommoju et.al (2014) 0.84 [0.77; 0.93]
Omitting Muller et.al (2003) 0.85 [0.77; 0.93]
Omitting Oh et.al (2000) 0.85 [0.77; 0.93]
Omitting Memisoglu et.al (2003) 0.85 [0.77; 0.93]
Omitting Mirzaei et.al (2009) 0.85 [0.77; 0.94]
Omitting Zhu et.al (2017) 0.85 [0.77; 0.93]
Omitting Wang et.al (2013) 0.87 [0.80; 0.94]
Omitting Ye et.al (2014) 0.84 [0.77; 0.93]
Omitting Bouassida et.al (2005) 0.84 [0.77; 0.93]
Omitting Vergotine et.al (2014) 0.84 [0.76; 0.92]
Omitting Moon et.al (2005) 0.85 [0.77; 0.94]
Omitting Pinterova et.al (2004) 0.85 [0.77; 0.93]
Omitting Evans et.al (2001) 0.84 [0.76; 0.92]
Omitting Badii et.al (2008) 0.85 [0.77; 0.93]
Omitting Motavallian et.al (2013) 0.85 [0.77; 0.93]
Omitting Phani et.al (2015) 0.85 [0.77; 0.93]
Omitting Sanghera et.al (2009) 0.84 [0.77; 0.93]
Omitting Majid et.al (2016) 0.86 [0.78; 0.94]
Omitting Bouhaha et.al (2008) 0.84 [0.76; 0.92]
Omitting Sramkova et.al (2002) 0.84 [0.76; 0.92]
Omitting Saleh et.al (2016) 0.84 [0.77; 0.93]
Omitting Paramasivam et.al (2016) 0.85 [0.77; 0.93]
Omitting Pattanayak et.al (2014) 0.85 [0.77; 0.93]
Omitting Vimaleswaran et.al (2010) 0.84 [0.76; 0.93]
Omitting Ringel et.al (1999) 0.84 [0.77; 0.93]
Omitting Nemoto et.al (2002) (A) 0.85 [0.77; 0.93]
Omitting Nemoto et.al (2002) (B) 0.85 [0.77; 0.93]
Omitting Meshkani et.al (2007) 0.85 [0.77; 0.94]
Omitting Chistiakov et.al (2010) 0.85 [0.77; 0.94]
Omitting Ghoussaini et.al (2005) 0.85 [0.77; 0.93]
Omitting Malecki et.al (2003) 0.84 [0.76; 0.92]
Omitting Hegele et.al (2000) 0.84 [0.76; 0.92]
Omitting Li et.al (2008) (A) (Uygur) 0.84 [0.77; 0.93]
Omitting Li et.al (2008) (B) (Kazak) 0.85 [0.77; 0.93]
Omitting Li et.al (2008) (C) (Han) 0.84 [0.77; 0.93]
Omitting Hansen et.al (2005) 0.84 [0.76; 0.93]
Omitting Meirhaeghe et.al (2000) 0.84 [0.77; 0.93]
Omitting Costa et.al (2009) 0.84 [0.76; 0.92]
Omitting Gragnoli et.al (2007) 0.85 [0.77; 0.93]
Omitting Erdogan et.al (2007) 0.85 [0.77; 0.93]
Omitting Tavares et.al (2005) 0.84 [0.76; 0.92]
Omitting Raza et.al (2012) 0.84 [0.76; 0.92]
Omitting Pei et.al (2013) 0.85 [0.77; 0.94]
Omitting Mohamed et.al (2007) 0.84 [0.76; 0.92]
Omitting Namvaran et.al (2011) 0.85 [0.77; 0.94]
Omitting Tariq et.al (2013) 0.84 [0.77; 0.93]
Omitting Doney et.al (2004) 0.85 [0.77; 0.94]
Omitting Mori et.al (2001) 0.85 [0.78; 0.94]
Omitting Clement et.al (2000) 0.85 [0.77; 0.93]
Omitting Avzaletdinova et.al (2016) 0.85 [0.77; 0.93]
Omitting Kao et.al (2003) 0.85 [0.77; 0.93]
Omitting Wang et.al (2009) 0.84 [0.77; 0.93]
Omitting Horiki et.al (2004) 0.85 [0.78; 0.94]
Omitting Lv et.al (2017) 0.83 [0.76; 0.91]
Random effects model 0.85 [0.77; 0.93]
0.8
1
1.25

A **B** **C**

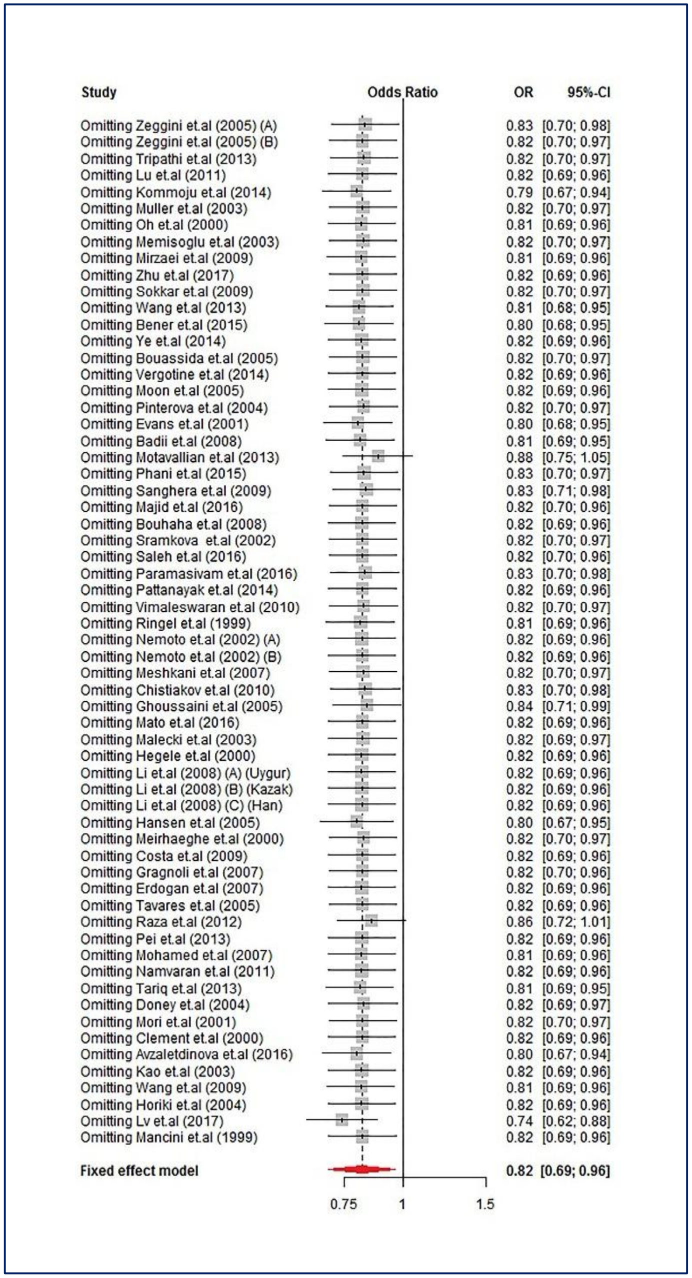
Study
Odds Ratio
OR 95%-CI
Omitting Zeggini et.al (2005) (A) 0.83 [0.70; 0.98]
Omitting Zeggini et.al (2005) (B) 0.82 [0.70; 0.97]
Omitting Tripathi et.al (2013) 0.82 [0.70; 0.97]
Omitting Lu et.al (2011) 0.82 [0.69; 0.96]
Omitting Kommoju et.al (2014) 0.79 [0.67; 0.94]
Omitting Muller et.al (2003) 0.82 [0.70; 0.97]
Omitting Oh et.al (2000) 0.81 [0.69; 0.96]
Omitting Memisoglu et.al (2003) 0.82 [0.70; 0.97]
Omitting Mirzaei et.al (2009) 0.81 [0.69; 0.96]
Omitting Zhu et.al (2017) 0.82 [0.69; 0.96]
Omitting Sokkar et.al (2009) 0.82 [0.70; 0.97]
Omitting Wang et.al (2013) 0.81 [0.68; 0.95]
Omitting Bener et.al (2015) 0.80 [0.68; 0.95]
Omitting Ye et.al (2014) 0.82 [0.69; 0.96]
Omitting Bouassida et.al (2005) 0.82 [0.70; 0.97]
Omitting Vergotine et.al (2014) 0.82 [0.69; 0.96]
Omitting Moon et.al (2005) 0.82 [0.69; 0.96]
Omitting Pinterova et.al (2004) 0.82 [0.70; 0.97]
Omitting Evans et.al (2001) 0.80 [0.68; 0.95]
Omitting Badii et.al (2008) 0.81 [0.69; 0.95]
Omitting Motavallian et.al (2013) 0.88 [0.75; 1.05]
Omitting Phani et.al (2015) 0.83 [0.70; 0.97]
Omitting Sanghera et.al (2009) 0.83 [0.71; 0.98]
Omitting Majid et.al (2016) 0.82 [0.70; 0.96]
Omitting Bouhaha et.al (2008) 0.82 [0.69; 0.96]
Omitting Sramkova et.al (2002) 0.82 [0.70; 0.97]
Omitting Saleh et.al (2016) 0.82 [0.70; 0.96]
Omitting Paramasivam et.al (2016) 0.83 [0.70; 0.98]
Omitting Pattanayak et.al (2014) 0.82 [0.69; 0.96]
Omitting Vimaleswaran et.al (2010) 0.82 [0.70; 0.97]
Omitting Ringel et.al (1999) 0.81 [0.69; 0.96]
Omitting Nemoto et.al (2002) (A) 0.82 [0.69; 0.96]
Omitting Nemoto et.al (2002) (B) 0.82 [0.69; 0.96]
Omitting Meshkani et.al (2007) 0.82 [0.70; 0.97]
Omitting Chistiakov et.al (2010) 0.83 [0.70; 0.98]
Omitting Ghoussaini et.al (2005) 0.84 [0.71; 0.99]
Omitting Mato et.al (2016) 0.82 [0.69; 0.96]
Omitting Malecki et.al (2003) 0.82 [0.69; 0.97]
Omitting Hegele et.al (2000) 0.82 [0.69; 0.96]
Omitting Li et.al (2008) (A) (Uygur) 0.82 [0.69; 0.96]
Omitting Li et.al (2008) (B) (Kazak) 0.82 [0.69; 0.96]
Omitting Li et.al (2008) (C) (Han) 0.82 [0.69; 0.96]
Omitting Hansen et.al (2005) 0.80 [0.67; 0.95]
Omitting Meirhaeghe et.al (2000) 0.82 [0.70; 0.97]
Omitting Costa et.al (2009) 0.82 [0.69; 0.96]
Omitting Gragnoli et.al (2007) 0.82 [0.70; 0.96]
Omitting Erdogan et.al (2007) 0.82 [0.69; 0.96]
Omitting Tavares et.al (2005) 0.82 [0.69; 0.96]
Omitting Raza et.al (2012) 0.86 [0.72; 1.01]
Omitting Pei et.al (2013) 0.82 [0.69; 0.96]
Omitting Mohamed et.al (2007) 0.81 [0.69; 0.96]
Omitting Namvaran et.al (2011) 0.82 [0.69; 0.96]
Omitting Tariq et.al (2013) 0.81 [0.69; 0.95]
Omitting Doney et.al (2004) 0.82 [0.69; 0.97]
Omitting Mori et.al (2001) 0.82 [0.70; 0.97]
Omitting Clement et.al (2000) 0.82 [0.69; 0.96]
Omitting Avzaletdinova et.al (2016) 0.80 [0.67; 0.94]
Omitting Kao et.al (2003) 0.82 [0.69; 0.96]
Omitting Wang et.al (2009) 0.81 [0.69; 0.96]
Omitting Horiki et.al (2004) 0.82 [0.69; 0.96]
Omitting Lv et.al (2017) 0.74 [0.62; 0.88]
Omitting Mancini et.al (1999) 0.82 [0.69; 0.96]
Fixed effect model 0.82 [0.69; 0.96]
0.75 1 1.5

D

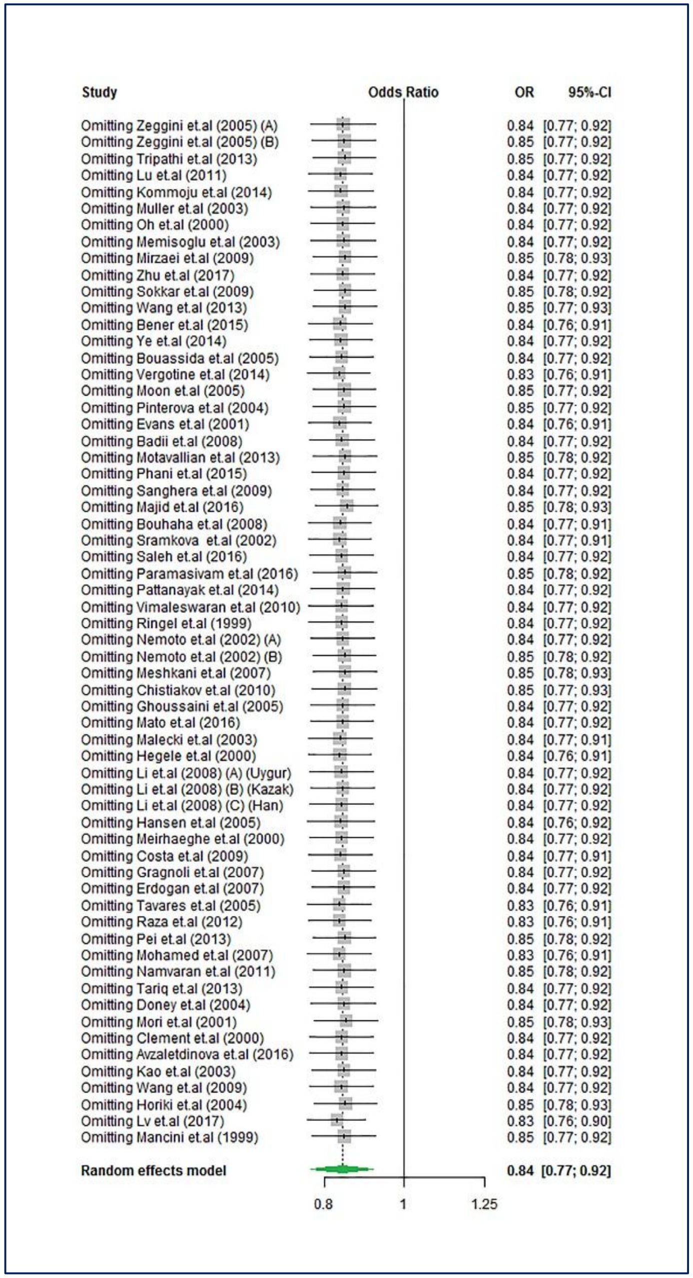
Study
Odds Ratio
OR 95%-CI
Omitting Zeggini et.al (2005) (A) 0.84 [0.77; 0.92]
Omitting Zeggini et.al (2005) (B) 0.85 [0.77; 0.92]
Omitting Tripathi et.al (2013) 0.85 [0.77; 0.92]
Omitting Lu et.al (2011) 0.84 [0.77; 0.92]
Omitting Kommoju et.al (2014) 0.84 [0.77; 0.92]
Omitting Muller et.al (2003) 0.84 [0.77; 0.92]
Omitting Oh et.al (2000) 0.84 [0.77; 0.92]
Omitting Memisoglu et.al (2003) 0.84 [0.77; 0.92]
Omitting Mirzaei et.al (2009) 0.85 [0.78; 0.93]
Omitting Zhu et.al (2017) 0.84 [0.77; 0.92]
Omitting Sokkar et.al (2009) 0.85 [0.78; 0.92]
Omitting Wang et.al (2013) 0.85 [0.77; 0.93]
Omitting Bener et.al (2015) 0.84 [0.76; 0.91]
Omitting Ye et.al (2014) 0.84 [0.77; 0.92]
Omitting Bouassida et.al (2005) 0.84 [0.77; 0.92]
Omitting Vergotine et.al (2014) 0.83 [0.76; 0.91]
Omitting Moon et.al (2005) 0.85 [0.77; 0.92]
Omitting Pinterova et.al (2004) 0.85 [0.77; 0.92]
Omitting Evans et.al (2001) 0.84 [0.76; 0.91]
Omitting Badii et.al (2008) 0.84 [0.77; 0.92]
Omitting Motavallian et.al (2013) 0.85 [0.78; 0.92]
Omitting Phani et.al (2015) 0.84 [0.77; 0.92]
Omitting Sanghera et.al (2009) 0.84 [0.77; 0.92]
Omitting Majid et.al (2016) 0.85 [0.78; 0.93]
Omitting Bouhaha et.al (2008) 0.84 [0.77; 0.91]
Omitting Sramkova et.al (2002) 0.84 [0.77; 0.91]
Omitting Saleh et.al (2016) 0.84 [0.77; 0.92]
Omitting Paramasivam et.al (2016) 0.85 [0.78; 0.92]
Omitting Pattanayak et.al (2014) 0.84 [0.77; 0.92]
Omitting Vimaleswaran et.al (2010) 0.84 [0.77; 0.92]
Omitting Ringel et.al (1999) 0.84 [0.77; 0.92]
Omitting Nemoto et.al (2002) (A) 0.84 [0.77; 0.92]
Omitting Nemoto et.al (2002) (B) 0.85 [0.78; 0.92]
Omitting Meshkani et.al (2007) 0.85 [0.78; 0.93]
Omitting Chistiakov et.al (2010) 0.85 [0.77; 0.93]
Omitting Ghoussaini et.al (2005) 0.84 [0.77; 0.92]
Omitting Mato et.al (2016) 0.84 [0.77; 0.92]
Omitting Malecki et.al (2003) 0.84 [0.77; 0.91]
Omitting Hegele et.al (2000) 0.84 [0.76; 0.91]
Omitting Li et.al (2008) (A) (Uygur) 0.84 [0.77; 0.92]
Omitting Li et.al (2008) (B) (Kazak) 0.84 [0.77; 0.92]
Omitting Li et.al (2008) (C) (Han) 0.84 [0.77; 0.92]
Omitting Hansen et.al (2005) 0.84 [0.76; 0.92]
Omitting Meirhaeghe et.al (2000) 0.84 [0.77; 0.92]
Omitting Costa et.al (2009) 0.84 [0.77; 0.91]
Omitting Gragnoli et.al (2007) 0.84 [0.77; 0.92]
Omitting Erdogan et.al (2007) 0.84 [0.77; 0.92]
Omitting Tavares et.al (2005) 0.83 [0.76; 0.91]
Omitting Raza et.al (2012) 0.83 [0.76; 0.91]
Omitting Pei et.al (2013) 0.85 [0.78; 0.92]
Omitting Mohamed et.al (2007) 0.83 [0.76; 0.91]
Omitting Namvaran et.al (2011) 0.85 [0.78; 0.92]
Omitting Tariq et.al (2013) 0.84 [0.77; 0.92]
Omitting Doney et.al (2004) 0.84 [0.77; 0.92]
Omitting Mori et.al (2001) 0.85 [0.78; 0.93]
Omitting Clement et.al (2000) 0.84 [0.77; 0.92]
Omitting Avzaletdinova et.al (2016) 0.84 [0.77; 0.92]
Omitting Kao et.al (2003) 0.84 [0.77; 0.92]
Omitting Wang et.al (2009) 0.84 [0.77; 0.92]
Omitting Horiki et.al (2004) 0.85 [0.78; 0.93]
Omitting Lv et.al (2017) 0.83 [0.76; 0.90]
Omitting Mancini et.al (1999) 0.85 [0.77; 0.92]
Random effects model 0.84 [0.77; 0.92]
0.8 1 1.25

E

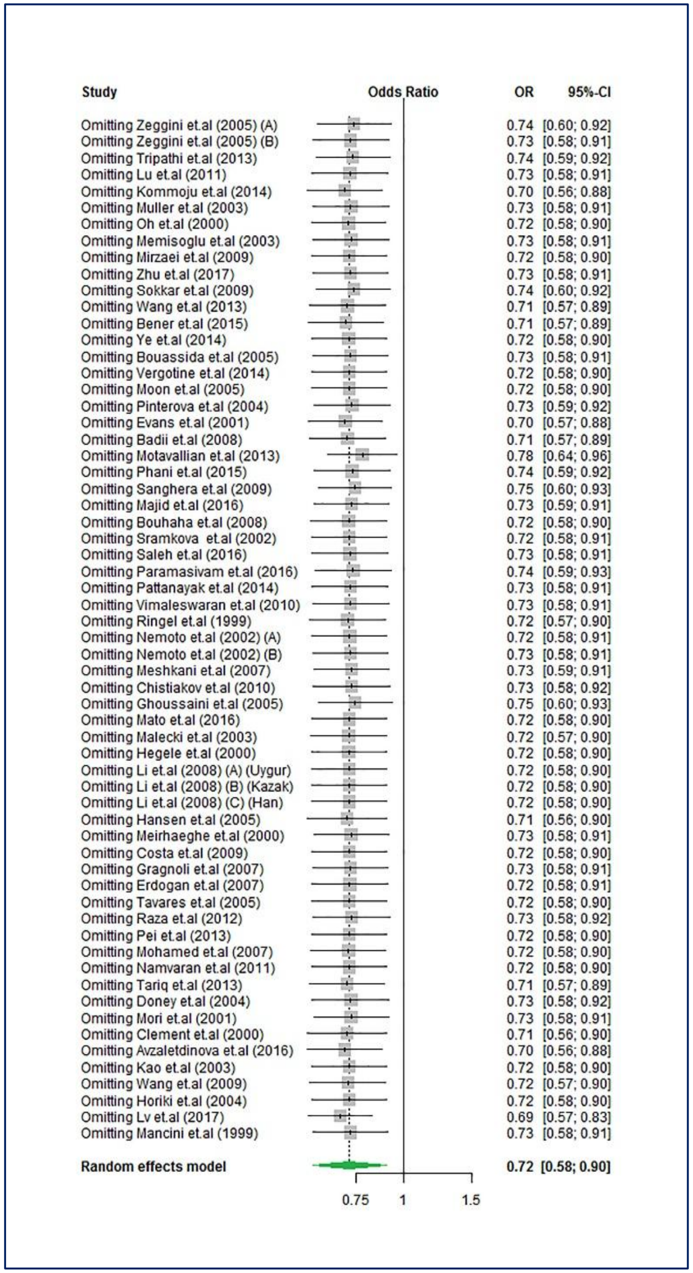
Study
Odds Ratio
OR 95%-CI
Omitting Zeggini et.al (2005) (A) 0.74 [0.60; 0.92]
Omitting Zeggini et.al (2005) (B) 0.73 [0.58; 0.91]
Omitting Tripathi et.al (2013) 0.74 [0.59; 0.92]
Omitting Lu et.al (2011) 0.73 [0.58; 0.91]
Omitting Kommoju et.al (2014) 0.70 [0.56; 0.88]
Omitting Muller et.al (2003) 0.73 [0.58; 0.91]
Omitting Oh et.al (2000) 0.72 [0.58; 0.90]
Omitting Memisoglu et.al (2003) 0.73 [0.58; 0.91]
Omitting Mirzaei et.al (2009) 0.72 [0.58; 0.90]
Omitting Zhu et.al (2017) 0.73 [0.58; 0.91]
Omitting Sokkar et.al (2009) 0.74 [0.60; 0.92]
Omitting Wang et.al (2013) 0.71 [0.57; 0.89]
Omitting Bener et.al (2015) 0.71 [0.57; 0.89]
Omitting Ye et.al (2014) 0.72 [0.58; 0.90]
Omitting Bouassida et.al (2005) 0.73 [0.58; 0.91]
Omitting Vergotine et.al (2014) 0.72 [0.58; 0.90]
Omitting Moon et.al (2005) 0.72 [0.58; 0.90]
Omitting Pinterova et.al (2004) 0.73 [0.59; 0.92]
Omitting Evans et.al (2001) 0.70 [0.57; 0.88]
Omitting Badii et.al (2008) 0.71 [0.57; 0.89]
Omitting Motavallian et.al (2013) 0.78 [0.64; 0.96]
Omitting Phani et.al (2015) 0.74 [0.59; 0.92]
Omitting Sanghera et.al (2009) 0.75 [0.60; 0.93]
Omitting Majid et.al (2016) 0.73 [0.59; 0.91]
Omitting Bouhaha et.al (2008) 0.72 [0.58; 0.90]
Omitting Sramkova et.al (2002) 0.72 [0.58; 0.91]
Omitting Saleh et.al (2016) 0.73 [0.58; 0.91]
Omitting Paramasivam et.al (2016) 0.74 [0.59; 0.93]
Omitting Pattanayak et.al (2014) 0.73 [0.58; 0.91]
Omitting Vimaleswaran et.al (2010) 0.73 [0.58; 0.91]
Omitting Ringel et.al (1999) 0.72 [0.57; 0.90]
Omitting Nemoto et.al (2002) (A) 0.72 [0.58; 0.91]
Omitting Nemoto et.al (2002) (B) 0.73 [0.58; 0.91]
Omitting Meshkani et.al (2007) 0.73 [0.59; 0.91]
Omitting Chistiakov et.al (2010) 0.73 [0.58; 0.92]
Omitting Ghoussaini et.al (2005) 0.75 [0.60; 0.93]
Omitting Mato et.al (2016) 0.72 [0.58; 0.90]
Omitting Malecki et.al (2003) 0.72 [0.57; 0.90]
Omitting Hegele et.al (2000) 0.72 [0.58; 0.90]
Omitting Li et.al (2008) (A) (Uygur) 0.72 [0.58; 0.90]
Omitting Li et.al (2008) (B) (Kazak) 0.72 [0.58; 0.90]
Omitting Li et.al (2008) (C) (Han) 0.72 [0.58; 0.90]
Omitting Hansen et.al (2005) 0.71 [0.56; 0.90]
Omitting Meirhaeghe et.al (2000) 0.73 [0.58; 0.91]
Omitting Costa et.al (2009) 0.72 [0.58; 0.90]
Omitting Gragnoli et.al (2007) 0.73 [0.58; 0.91]
Omitting Erdogan et.al (2007) 0.72 [0.58; 0.91]
Omitting Tavares et.al (2005) 0.72 [0.58; 0.90]
Omitting Raza et.al (2012) 0.73 [0.58; 0.92]
Omitting Pei et.al (2013) 0.72 [0.58; 0.90]
Omitting Mohamed et.al (2007) 0.72 [0.58; 0.90]
Omitting Namvaran et.al (2011) 0.72 [0.58; 0.90]
Omitting Tariq et.al (2013) 0.71 [0.57; 0.89]
Omitting Doney et.al (2004) 0.73 [0.58; 0.92]
Omitting Mori et.al (2001) 0.73 [0.58; 0.91]
Omitting Clement et.al (2000) 0.71 [0.56; 0.90]
Omitting Avzaletdinova et.al (2016) 0.70 [0.56; 0.88]
Omitting Kao et.al (2003) 0.72 [0.58; 0.90]
Omitting Wang et.al (2009) 0.72 [0.57; 0.90]
Omitting Horiki et.al (2004) 0.72 [0.58; 0.90]
Omitting Lv et.al (2017) 0.69 [0.57; 0.83]
Omitting Mancini et.al (1999) 0.73 [0.58; 0.91]
Random effects model 0.72 [0.58; 0.90]
0.75 1 1.5

F

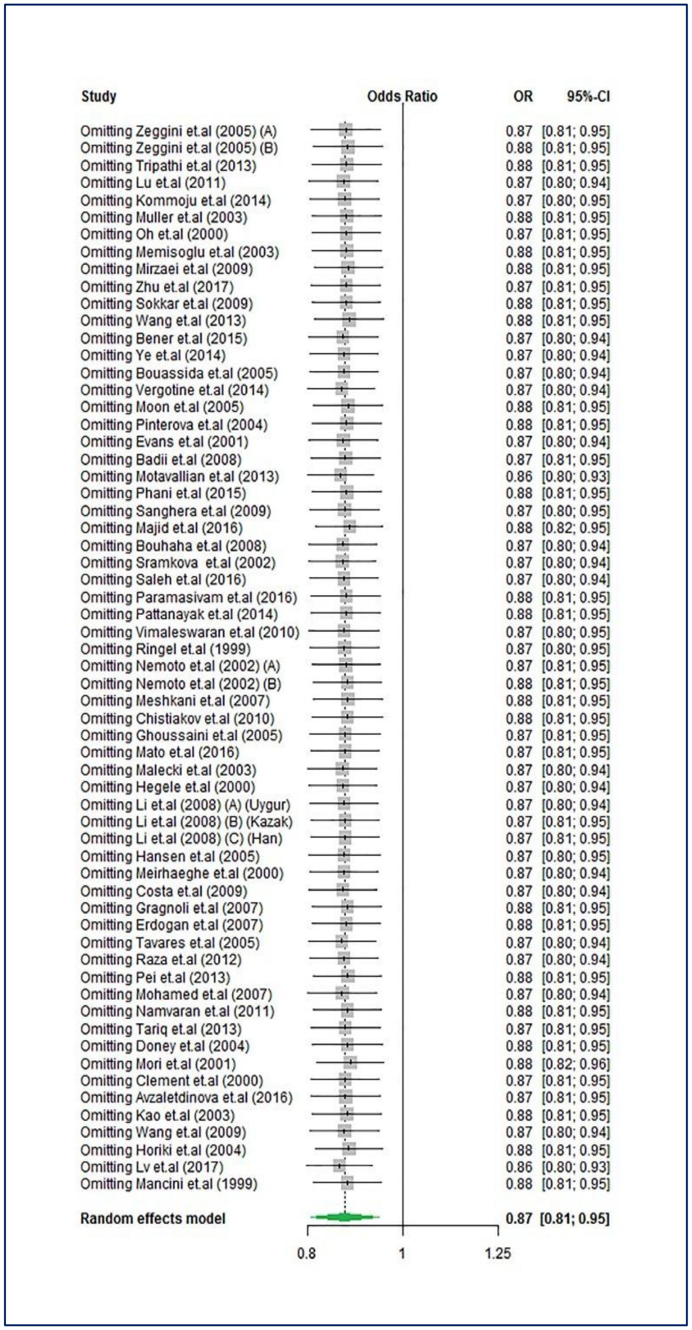

G

Figure S5. Influence plots of the included studies. “A” represents allele (G vs. C); “B” represents homozygote (GG vs. CC); “C” represents heterozygote (CG vs. CC); “D” represents additive (GG vs. CG); “E” represents dominant (CG+GG vs. CC); “F” represents recessive (GG vs. CC+CG); “G” co-dominant model (CG vs. CC+GG). Vertical and horizontal lines represent ORs and 95% CIs pooled by successively excluding one study in turn. Red stroked diamonds represent the overall estimates (pooled ORs and 95% CIs) of population with fixed effect model, and green stroked diamonds symbolize results pooled with random effects model. 95% CI = 95% confidence interval, OR = odds ratio.
