# Supplementary material for: PPARG (Pro12Ala) genetic variant and risk of T2DM: a systematic review and meta-analysis
Source: Sci Rep. 2020 Jul 29;10:12764. doi: 10.1038/s41598-020-69363-7 (PMC7391673; doi:10.1038/s41598-020-69363-7)
Supplement: Supplementary file 6 — Supplementary Information 6. [file 41598_2020_69363_MOESM6_ESM.pdf]

PPARG (Pro12Ala) genetic variant and risk of T2DM: a systematic review and meta-analysis

Negar Sarhangi [1], Farshad Sharifi [2], Leila Hashemian [3], Maryam Hassani Doabsari [3], Katayoun Heshmatzad [3], Marzieh Rahbaran [3], Seyed Hamid Jamaldini [3], Hamid Reza Aghaei Meybodi [1, 4], Mandana Hasanzad [1,3,*]

[1] Personalized Medicine Research Center, Endocrinology and Metabolism Clinical Sciences Institute, Tehran University of Medical Sciences, Tehran 1411413137, Iran

[2] Elderly Health Research Center, Endocrinology and Metabolism Population Sciences Institute, Tehran University of Medical Sciences, Tehran 1411413137, Iran

[3] Medical Genomics Research Center, Tehran Medical Sciences, Islamic Azad University, Tehran 1916893813, Iran

[4] Endocrinology and Metabolism Research Center, Endocrinology and Metabolism Clinical Sciences Institute, Tehran University of Medical Sciences, Tehran 1411413137, Iran

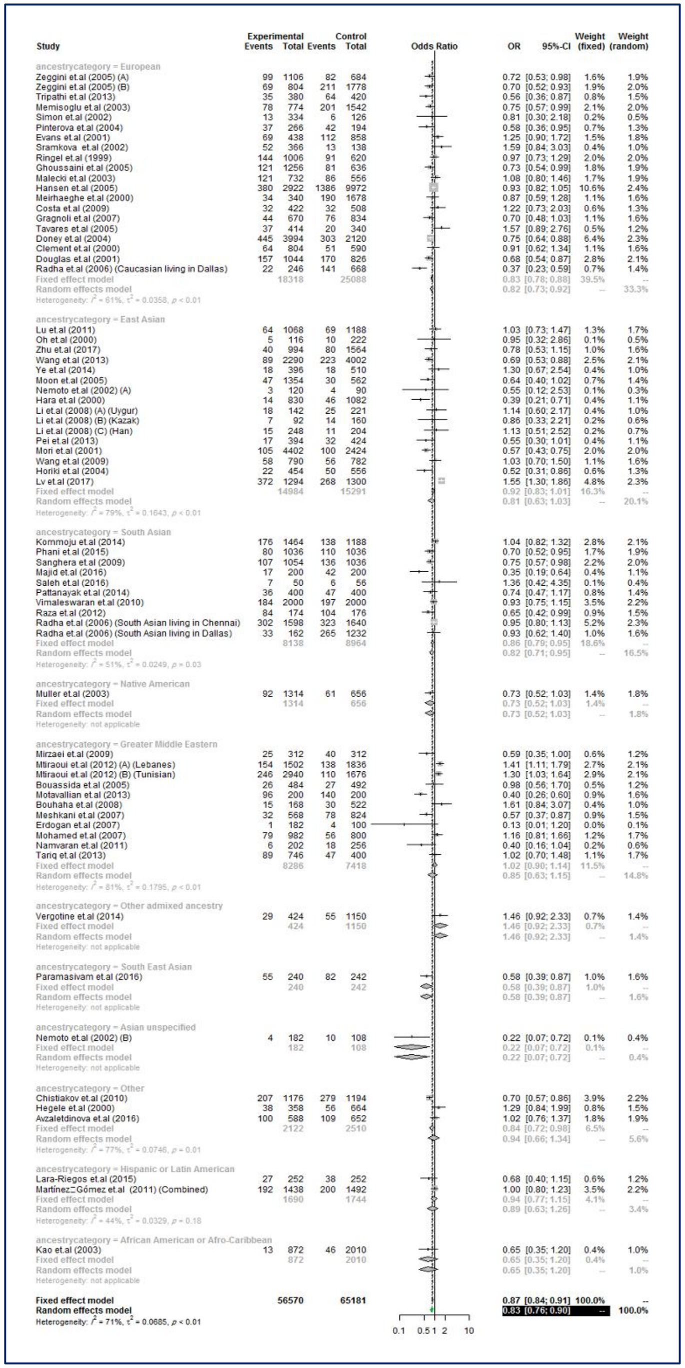

A

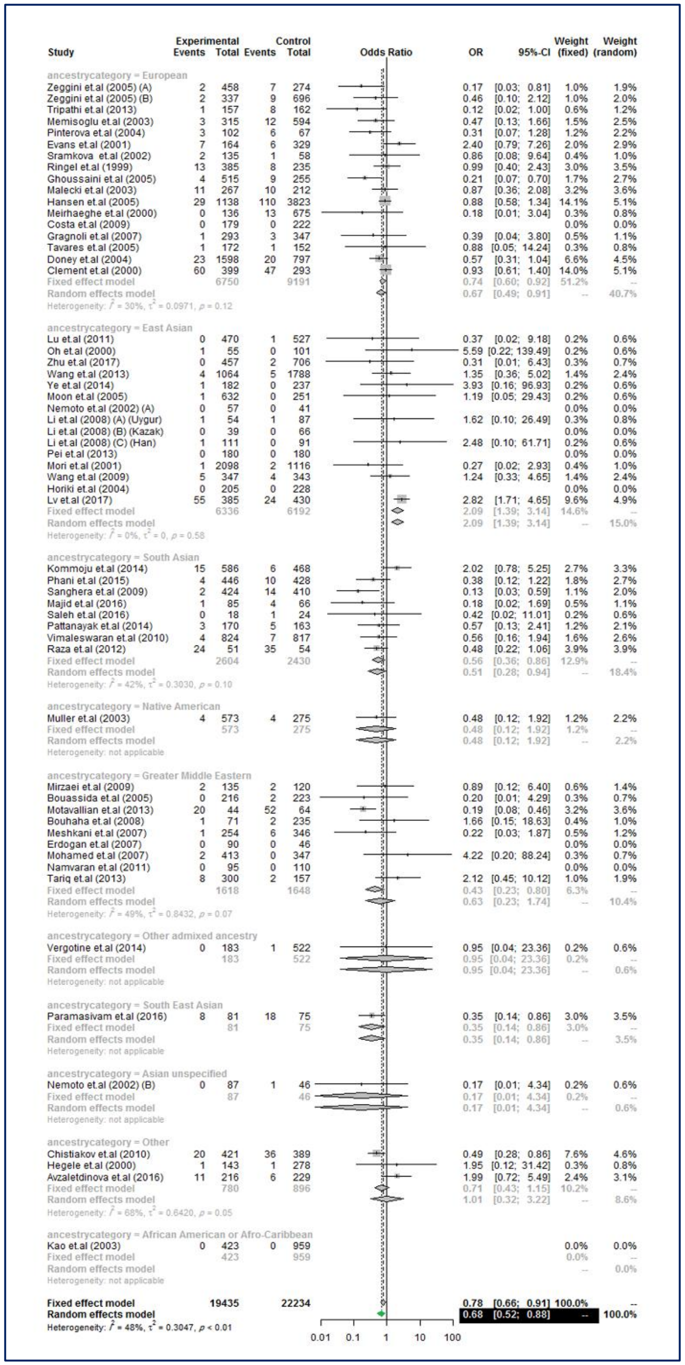

B

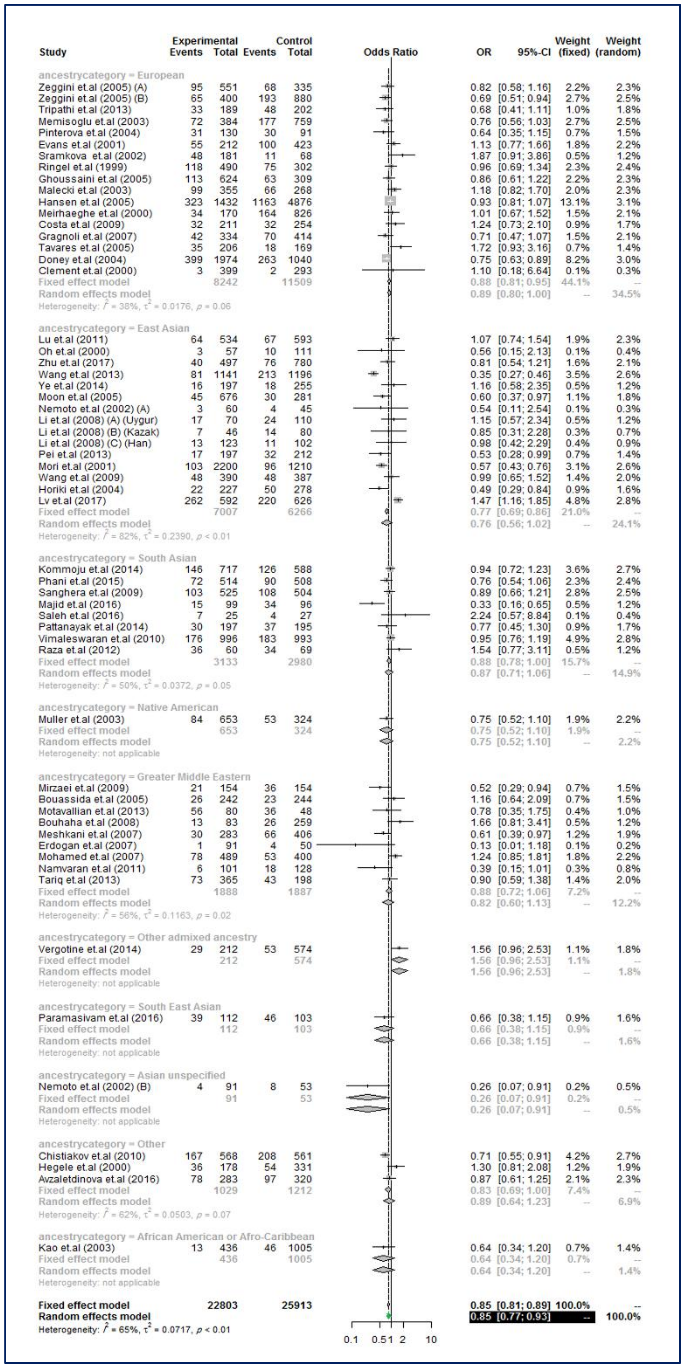

C

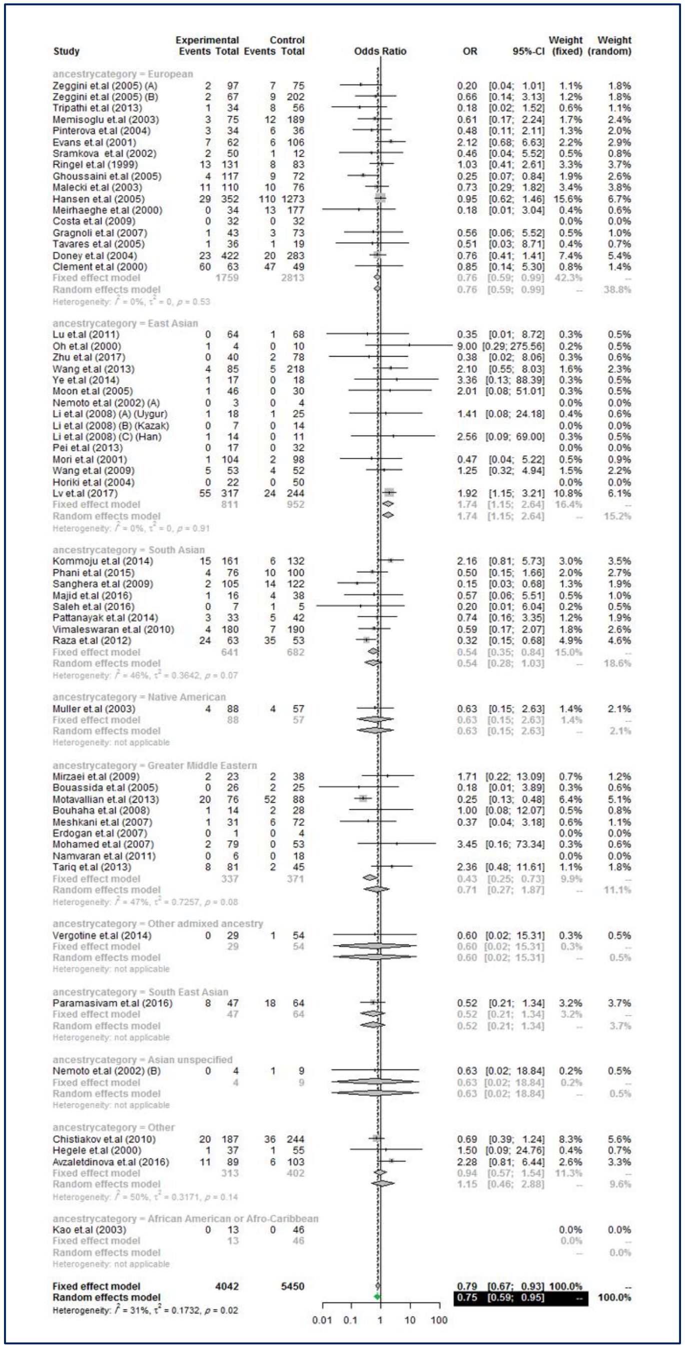

D

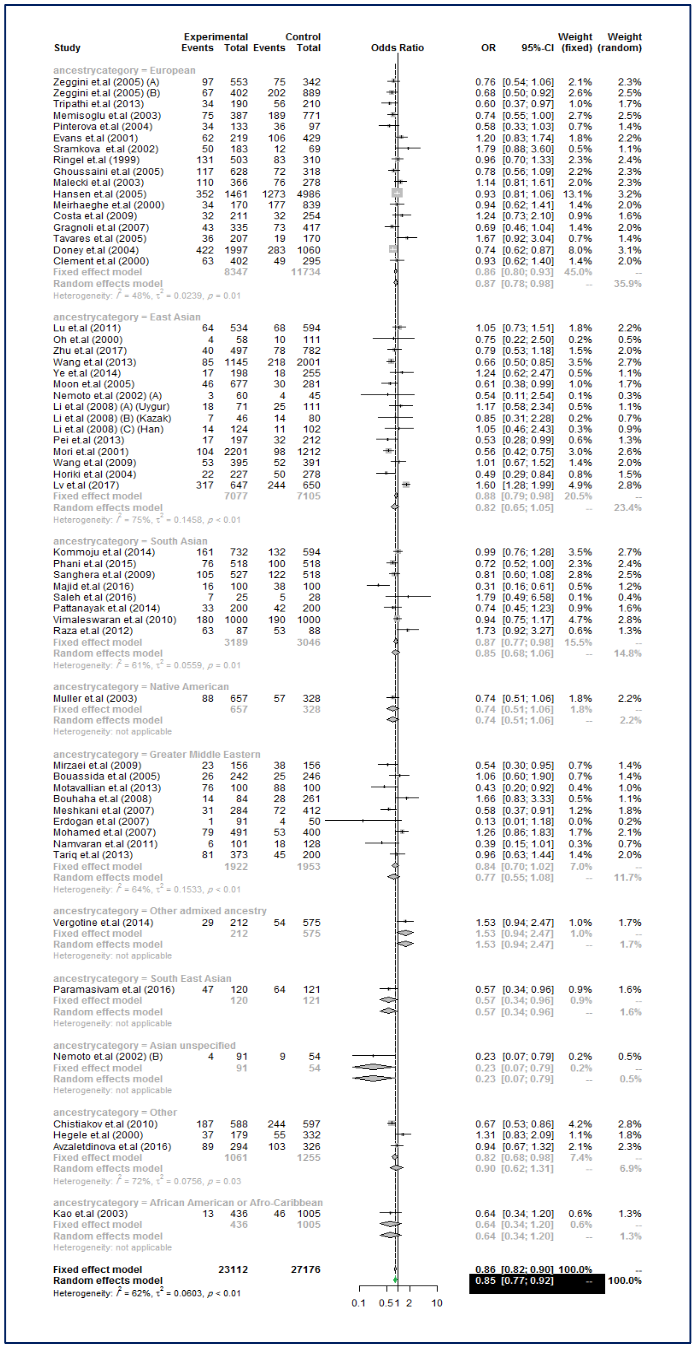

E

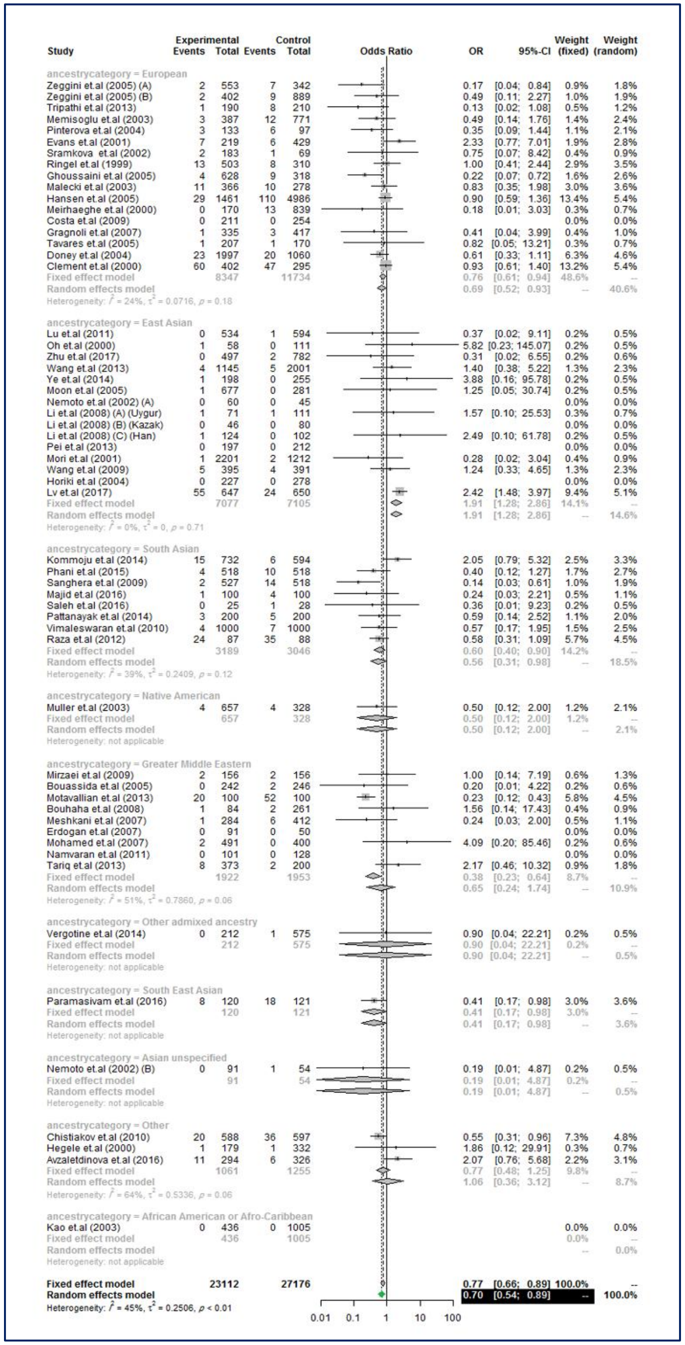

F

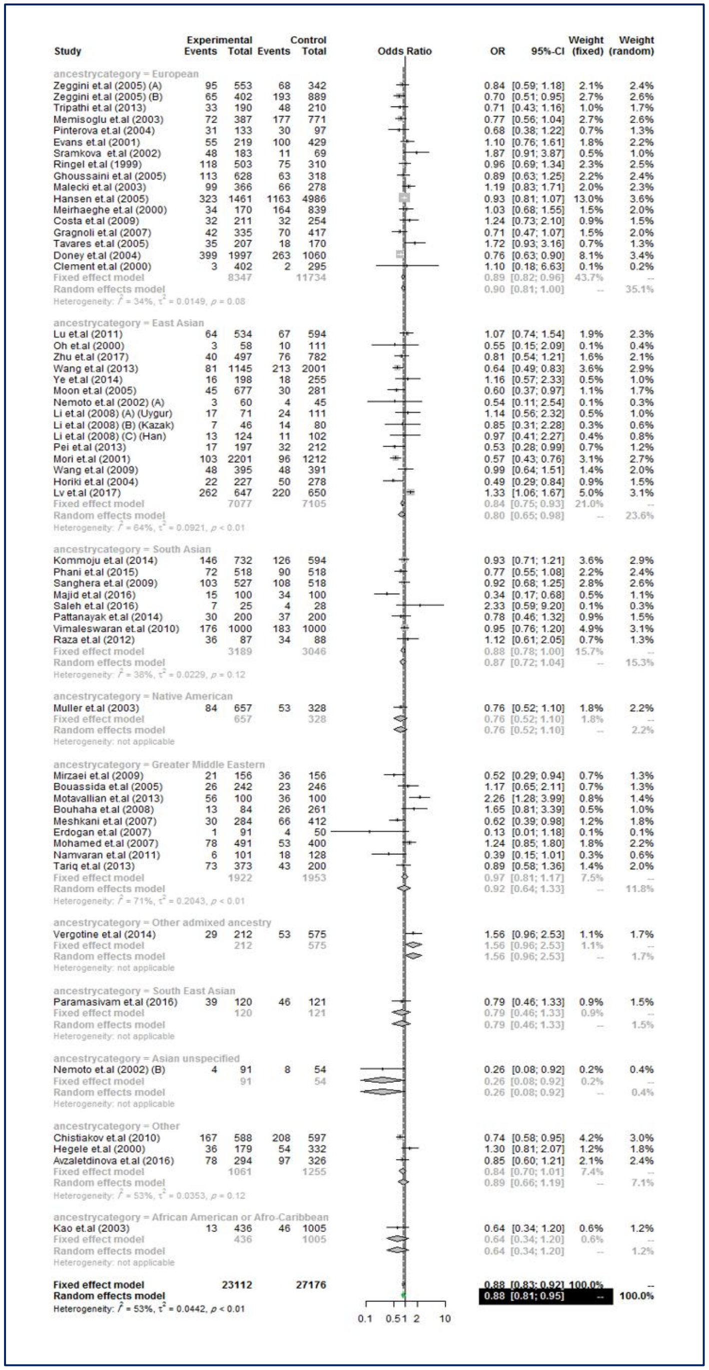

G

Figure S6. Sensitivity analysis according to studies that did not meet HWE. “A” represents allele (G vs. C); “B” represents homozygote (GG vs. CC); “C” represents heterozygote (CG vs. CC); “D” represents additive (GG vs. CG); “E” represents dominant (CG+GG vs. CC); “F” represents recessive (GG vs. CC+CG); “G” co-dominant model (CG vs. CC+GG). Vertical and horizontal lines represent ORs and the corresponding 95% CIs of each study. Black highlight represents the overall estimates (pooled ORs and 95% CIs) of population with random effects model. 95% CI = 95% confidence interval, OR = odds ratio.
