# Supplementary material for: PPARG (Pro12Ala) genetic variant and risk of T2DM: a systematic review and meta-analysis
Source: Sci Rep. 2020 Jul 29;10:12764. doi: 10.1038/s41598-020-69363-7 (PMC7391673; doi:10.1038/s41598-020-69363-7)
Supplement: Supplementary file 9 — Supplementary Information 9. [file 41598_2020_69363_MOESM9_ESM.pdf]

PPARG (Pro12Ala) genetic variant and risk of T2DM: a systematic review and meta-analysis

Negar Sarhangi [1], Farshad Sharifi [2], Leila Hashemian [3], Maryam Hassani Doabsari [3], Katayoun Heshmatzad [3], Marzieh Rahbaran [3], Seyed Hamid Jamaldini [3], Hamid Reza Aghaei Meybodi [1, 4], Mandana Hasanzad [1,3,*]

[1] Personalized Medicine Research Center, Endocrinology and Metabolism Clinical Sciences Institute, Tehran University of Medical Sciences, Tehran 1411413137, Iran

[2] Elderly Health Research Center, Endocrinology and Metabolism Population Sciences Institute, Tehran University of Medical Sciences, Tehran 1411413137, Iran

[3] Medical Genomics Research Center, Tehran Medical Sciences, Islamic Azad University, Tehran 1916893813, Iran

[4] Endocrinology and Metabolism Research Center, Endocrinology and Metabolism Clinical Sciences Institute, Tehran University of Medical Sciences, Tehran 1411413137, Iran

Electronic Search Databases

1. Pubmed Search Strategy

((diabetes[tiab] AND "Type 2"[tiab]) OR "diabetes type 2"[ti] OR "diabetes type II"[ti] OR "type II diabetes"[ti] OR "dm 2"[ti] OR "Type 2 dibetes mellitus"[ti] OR "diabetes mellitus type 2"[ti] OR "diabetes mellitus type ii"[ti] OR ("Diabetes Mellitus"[ti] AND "Type II"[ti]) OR "Type 2 Diabetes Mellitus"[ti] OR ("diabetes mellitus"[ti] AND "type 2"[ti]) OR "insulin independent diabetes"[ti] OR "non insulin dependent diabetes"[ti] OR "noninsulin dependent diabetes"[ti] OR ("Diabetes Mellitus"[ti] AND noninsulin-dependent[ti]) OR ("Diabetes Mellitus"[ti] AND "Non Insulin Dependent"[ti]) OR ("Diabetes Mellitus"[ti] AND non-insulin-dependent[ti]) OR "Non-Insulin-Dependent Diabetes Mellitus"[ti] OR "insulin independent diabetes mellitus"[ti] OR ("Diabetes Mellitus"[ti] AND "Noninsulin Dependent"[ti]) OR "Noninsulin-Dependent Diabetes Mellitus"[ti] OR "Noninsulin Dependent Diabetes Mellitus"[ti] OR niddm[ti] OR "Maturity-Onset Diabetes Mellitus"[ti] OR "Maturity Onset Diabetes Mellitus"[ti] OR "Maturity-Onset Diabetes"[ti] OR (diabetes[ti] AND maturity-onset[ti]) OR "Maturity Onset Diabetes" OR mody[ti] OR "maturity onset diabetes of the young"[ti] OR "adult onset diabetes"[ti] OR "adult onset diabetes mellitus"[ti] OR (diabetes[ti] AND "adult onset"[ti]) OR ("Diabetes Mellitus"[ti] AND adult-onset[ti]) OR ("Diabetes Mellitus"[ti] AND "Adult Onset"[ti]) OR "Adult-Onset Diabetes Mellitus"[ti] OR ("Diabetes Mellitus"[ti] AND ketosis-resistant[ti]) OR ("Diabetes Mellitus"[ti] AND "Ketosis Resistant"[ti]) OR "Ketosis-Resistant Diabetes Mellitus"[ti] OR "ketosis resistant diabetes mellitus"[ti] OR ("Diabetes Mellitus"[ti] AND stable[ti]) OR "Stable Diabetes Mellitus"[ti] OR ("Diabetes Mellitus"[ti] AND maturity-onset[ti]) OR ("Diabetes Mellitus"[ti] AND "Maturity Onset"[ti]) OR ("Diabetes Mellitus"[ti] AND slow-onset[ti]) OR ("Diabetes Mellitus"[ti] AND "Slow Onset"[ti]) OR "Slow-Onset Diabetes Mellitus"[ti]) AND

(ppargamma[ti] OR "PPAR gamma"[ti] OR ppar-gamma[ti] OR ppar-gamma[ti] OR "PPAR gamma"[ti] OR ppargamma[ti] OR "PPAR G"[ti] OR ppar-g[ti] OR "peroxisome proliferator-activated receptor gamma"[tiab] OR "peroxisome proliferator-activated receptor G"[ti] OR "Peroxisome Proliferator-Activated Nuclear Receptor Gamma"[ti] OR "Peroxisome Proliferator Activated Receptor gamma"[ti] OR ("Peroxisome Proliferative Activated Receptor"[ti] AND gamma[ti]) OR "PPARgamma3"[ti] OR PPARgamma2[ti] OR mPPARgamma1[ti] OR mPPARgamma2[ti] OR PPARG1[ti] OR PPARG2[ti] OR PPARG3[ti]) AND 1990/01/01:2017/10/30[dp]

2. Scopus Search Strategy

((TITLE-ABS(diabetes) AND TITLE-ABS("Type 2")) OR TITLE("diabetes type 2") OR TITLE("diabetes type II") OR TITLE("type II diabetes") OR TITLE("dm 2") OR TITLE("Type 2 dibetes mellitus") OR TITLE("diabetes mellitus type 2") OR TITLE("diabetes mellitus type ii") OR (TITLE("Diabetes Mellitus") AND TITLE("Type II")) OR TITLE("Type 2 Diabetes Mellitus") OR (TITLE("diabetes mellitus") AND TITLE("type 2")) OR TITLE("insulin independent diabetes") OR TITLE("non insulin dependent diabetes") OR TITLE("noninsulin dependent diabetes") OR (TITLE("Diabetes Mellitus") AND TITLE(noninsulin-dependent)) OR (TITLE("Diabetes Mellitus") AND TITLE("Non Insulin Dependent")) OR (TITLE("Diabetes Mellitus") AND TITLE(non-insulin-dependent)) OR TITLE("Non-Insulin-Dependent Diabetes Mellitus") OR TITLE("insulin independent diabetes mellitus") OR (TITLE("Diabetes Mellitus") AND TITLE("Noninsulin Dependent")) OR TITLE("Noninsulin-Dependent Diabetes Mellitus")

OR TITLE("Noninsulin Dependent Diabetes Mellitus") OR TITLE(niddm) OR TITLE("Maturity-Onset Diabetes Mellitus") OR TITLE("Maturity Onset Diabetes Mellitus") OR TITLE("Maturity-Onset Diabetes") OR (TITLE(diabetes) AND TITLE(maturity-onset)) OR TITLE("Maturity Onset Diabetes") OR TITLE(mody) OR TITLE("maturity onset diabetes of the young") OR TITLE("adult onset diabetes") OR TITLE("adult onset diabetes mellitus") OR (TITLE(diabetes) AND TITLE("adult onset")) OR (TITLE("Diabetes Mellitus") AND TITLE(adult-onset)) OR (TITLE("Diabetes Mellitus") AND TITLE("Adult Onset")) OR TITLE("Adult-Onset Diabetes Mellitus") OR (TITLE("Diabetes Mellitus") AND TITLE(ketosis-resistant)) OR (TITLE("Diabetes Mellitus") AND TITLE("Ketosis Resistant")) OR TITLE("Ketosis-Resistant Diabetes Mellitus") OR TITLE("ketosis resistant diabetes mellitus") OR (TITLE("Diabetes Mellitus") AND TITLE(stable)) OR TITLE("Stable Diabetes Mellitus") OR (TITLE("Diabetes Mellitus") AND TITLE(maturity-onset)) OR (TITLE("Diabetes Mellitus") AND TITLE("Maturity Onset")) OR (TITLE("Diabetes Mellitus") AND TITLE(slow-onset)) OR (TITLE("Diabetes Mellitus") AND TITLE("Slow Onset")) OR TITLE("Slow-Onset Diabetes Mellitus")) AND (TITLE(ppargamma) OR TITLE("PPAR gamma") OR TITLE(ppar-gamma) OR TITLE(ppar-gamma) OR TITLE("PPAR gamma") OR TITLE(ppargamma) OR TITLE("PPAR G") OR TITLE(ppar-g) OR TITLE-ABS("peroxisome proliferator-activated receptor gamma") OR TITLE("peroxisome proliferator-activated receptor G") OR TITLE("Peroxisome Proliferator-Activated Nuclear Receptor Gamma") OR TITLE("Peroxisome Proliferator Activated Receptor gamma") OR (TITLE("Peroxisome Proliferative Activated Receptor") AND TITLE(gamma)) OR TITLE("PPARgamma3") OR TITLE(PPARgamma2) OR TITLE(mPPARgamma1) OR TITLE(mPPARgamma2) OR TITLE(PPARG1) OR TITLE(PPARG2) OR TITLE(PPARG3)) AND ((PUBYEAR > 1989 AND PUBYEAR < 2016) OR (PUBDATETXT(JAN 2017) OR

PUBDATETXT(FEB 2017) OR PUBDATETXT(MAR 2017) OR PUBDATETXT(APR 2017) OR PUBDATETXT(MAY 2017) OR PUBDATETXT(JUNE 2017) OR PUBDATETXT(JULY 2017) OR PUBDATETXT(AUG 2017) OR PUBDATETXT(SEP 2017) OR PUBDATETXT(OCT 2017)))

3. Web of Sciences Search Strategy

((TS=(diabetes) AND TS=("Type 2")) OR TI=("diabetes type 2") OR TI=("diabetes type II") OR TI=("type II diabetes") OR TI=("dm 2") OR TI=("Type 2 dibetes mellitus") OR TI=("diabetes mellitus type 2") OR TI=("diabetes mellitus type ii") OR (TI=("Diabetes Mellitus") AND TI=("Type II")) OR TI=("Type 2 Diabetes Mellitus") OR (TI=("diabetes mellitus") AND TI=("type 2")) OR TI=("insulin independent diabetes") OR TI=("non insulin dependent diabetes") OR TI=("noninsulin dependent diabetes") OR (TI=("Diabetes Mellitus") AND TI=(noninsulin-dependent)) OR (TI=("Diabetes Mellitus") AND TI=("Non Insulin Dependent")) OR (TI=("Diabetes Mellitus") AND TI=(non-insulin-dependent)) OR TI=("Non-Insulin-Dependent Diabetes Mellitus") OR TI=("insulin independent diabetes mellitus") OR (TI=("Diabetes Mellitus") AND TI=("Noninsulin Dependent")) OR TI=("Noninsulin-Dependent Diabetes Mellitus") OR TI=("Noninsulin Dependent Diabetes Mellitus") OR TI=(niddm) OR TI=("Maturity-Onset Diabetes Mellitus") OR TI=("Maturity Onset Diabetes Mellitus") OR TI=("Maturity-Onset Diabetes") OR (TI=(diabetes) AND TI=(maturity-onset)) OR TI=("Maturity Onset Diabetes") OR TI=(mody) OR TI=("maturity onset diabetes of the young") OR TI=("adult onset diabetes") OR TI=("adult onset diabetes mellitus") OR (TI=(diabetes) AND TI=("adult onset")) OR (TI=("Diabetes Mellitus") AND TI=(adult-onset)) OR (TI=("Diabetes Mellitus")

AND TI=("Adult Onset")) OR TI=("Adult-Onset Diabetes Mellitus") OR (TI=("Diabetes Mellitus") AND TI=(ketosis-resistant)) OR (TI=("Diabetes Mellitus") AND TI=("Ketosis Resistant")) OR TI=("Ketosis-Resistant Diabetes Mellitus") OR TI=("ketosis resistant diabetes mellitus") OR (TI=("Diabetes Mellitus") AND TI=(stable)) OR TI=("Stable Diabetes Mellitus") OR (TI=("Diabetes Mellitus") AND TI=(maturity-onset)) OR (TI=("Diabetes Mellitus") AND TI=("Maturity Onset")) OR (TI=("Diabetes Mellitus") AND TI=(slow-onset)) OR (TI=("Diabetes Mellitus") AND TI=("Slow Onset")) OR TI=("Slow-Onset Diabetes Mellitus")) AND (TI=(ppargamma) OR TI=("PPAR gamma") OR TI=(ppar-gamma) OR TI=(ppar-gamma) OR TI=("PPAR gamma") OR TI=(ppargamma) OR TI=("PPAR G") OR TI=(ppar-g) OR TS=("peroxisome proliferator-activated receptor gamma") OR TI=("peroxisome proliferator-activated receptor G") OR TI=("Peroxisome Proliferator-Activated Nuclear Receptor Gamma") OR TI=("Peroxisome Proliferator Activated Receptor gamma") OR (TI=("Peroxisome Proliferative Activated Receptor") AND TI=(gamma)) OR TI=("PPARgamma3") OR TI=(PPARgamma2) OR TI=(mPPARgamma1) OR TI=(mPPARgamma2) OR TI=(PPARG1) OR TI=(PPARG2) OR TI=(PPARG3)) AND PY= (1989-2018)

4. Embase Search Strategy

((diabetes:ti,ab AND "Type 2":ti,ab) OR "diabetes type 2":ti OR "diabetes type II":ti OR "type II diabetes":ti OR "dm 2":ti OR "Type 2 dibetes mellitus":ti OR "diabetes mellitus type 2":ti OR "diabetes mellitus type ii":ti OR ("Diabetes Mellitus":ti AND "Type II":ti) OR "Type 2 Diabetes Mellitus":ti OR ("diabetes mellitus":ti AND "type 2":ti) OR "insulin independent diabetes":ti OR "non insulin dependent diabetes":ti OR "noninsulin dependent diabetes":ti OR ("Diabetes Mellitus":ti AND noninsulin-dependent:ti) OR ("Diabetes Mellitus":ti AND "Non Insulin

Dependent":ti) OR ("Diabetes Mellitus":ti AND non-insulin-dependent:ti) OR "Non-Insulin-Dependent Diabetes Mellitus":ti OR "insulin independent diabetes mellitus":ti OR ("Diabetes Mellitus":ti AND "Noninsulin Dependent":ti) OR "Noninsulin-Dependent Diabetes Mellitus":ti OR "Noninsulin Dependent Diabetes Mellitus":ti OR niddm:ti OR "Maturity-Onset Diabetes Mellitus":ti OR "Maturity Onset Diabetes Mellitus":ti OR "Maturity-Onset Diabetes":ti OR (diabetes:ti AND maturity-onset:ti) OR "Maturity Onset Diabetes" OR mody:ti OR "maturity onset diabetes of the young":ti OR "adult onset diabetes":ti OR "adult onset diabetes mellitus":ti OR (diabetes:ti AND "adult onset":ti) OR ("Diabetes Mellitus":ti AND adult-onset:ti) OR ("Diabetes Mellitus":ti AND "Adult Onset":ti) OR "Adult-Onset Diabetes Mellitus":ti OR ("Diabetes Mellitus":ti AND ketosis-resistant:ti) OR ("Diabetes Mellitus":ti AND "Ketosis Resistant":ti) OR "Ketosis-Resistant Diabetes Mellitus":ti OR "ketosis resistant diabetes mellitus":ti OR ("Diabetes Mellitus":ti AND stable:ti) OR "Stable Diabetes Mellitus":ti OR ("Diabetes Mellitus":ti AND maturity-onset:ti) OR ("Diabetes Mellitus":ti AND "Maturity Onset":ti) OR ("Diabetes Mellitus":ti AND slow-onset:ti) OR ("Diabetes Mellitus":ti AND "Slow Onset":ti) OR "Slow-Onset Diabetes Mellitus":ti) AND (ppargamma:ti OR "PPAR gamma":ti OR ppar-gamma:ti OR ppar-gamma:ti OR "PPAR gamma":ti OR ppargamma:ti OR "PPAR G":ti OR ppar-g:ti OR "peroxisome proliferator-activated receptor gamma":ti,ab OR "peroxisome proliferator-activated receptor G":ti OR "Peroxisome Proliferator-Activated Nuclear Receptor Gamma":ti OR "Peroxisome Proliferator Activated Receptor gamma":ti OR ("Peroxisome Proliferative Activated Receptor":ti AND gamma:ti) OR "PPARgamma3":ti OR PPARgamma2:ti OR mPPARgamma1:ti OR mPPARgamma2:ti OR PPARG1:ti OR PPARG2:ti OR PPARG3:ti) AND [1990-2017]/PY

5. ProQuest Search Strategy

((AB,TI(diabetes) AND AB,TI("Type 2")) OR TI("diabetes type 2") OR TI("diabetes type II") OR TI("type II diabetes") OR TI("dm 2") OR TI("Type 2 dibetes mellitus") OR TI("diabetes mellitus type 2") OR TI("diabetes mellitus type ii") OR (TI("Diabetes Mellitus") AND TI("Type II")) OR TI("Type 2 Diabetes Mellitus") OR (TI("diabetes mellitus") AND TI("type 2")) OR TI("insulin independent diabetes") OR TI("non insulin dependent diabetes") OR TI("noninsulin dependent diabetes") OR (TI("Diabetes Mellitus") AND TI(noninsulin-dependent)) OR (TI("Diabetes Mellitus") AND TI("Non Insulin Dependent")) OR (TI("Diabetes Mellitus") AND TI(non-insulin-dependent)) OR TI("Non-Insulin-Dependent Diabetes Mellitus") OR TI("insulin independent diabetes mellitus") OR (TITLE("Diabetes Mellitus") AND TI("Noninsulin Dependent")) OR TI("Noninsulin-Dependent Diabetes Mellitus") OR TI("Noninsulin Dependent Diabetes Mellitus") OR TI(niddm) OR TI("Maturity-Onset Diabetes Mellitus") OR TI("Maturity Onset Diabetes Mellitus") OR TI("Maturity-Onset Diabetes") OR (TI(diabetes) AND TI(maturity-onset)) OR TI("Maturity Onset Diabetes") OR TI(mody) OR TI("maturity onset diabetes of the young") OR TI("adult onset diabetes") OR TI("adult onset diabetes mellitus") OR (TI(diabetes) AND TI("adult onset")) OR (TI("Diabetes Mellitus") AND TI(adult-onset)) OR (TI("Diabetes Mellitus") AND TI("Adult Onset")) OR TI("Adult-Onset Diabetes Mellitus") OR (TI("Diabetes Mellitus") AND TI(ketosis-resistant)) OR (TI("Diabetes Mellitus") AND TI("Ketosis Resistant")) OR TI("Ketosis-Resistant Diabetes Mellitus") OR TI("ketosis resistant diabetes mellitus") OR (TI("Diabetes Mellitus") AND TI(stable)) OR TI("Stable Diabetes Mellitus") OR (TI("Diabetes Mellitus") AND TI(maturity-onset)) OR (TI("Diabetes Mellitus") AND TI("Maturity Onset")) OR (TI("Diabetes Mellitus") AND TI(slow-onset)) OR (TI("Diabetes Mellitus") AND TI("Slow

Onset")) OR TI("Slow-Onset Diabetes Mellitus")) AND (TI(ppargamma) OR TI("PPAR gamma") OR TI(ppar-gamma) OR TI(ppar-gamma) OR TI("PPAR gamma") OR TI(ppargamma) OR TI("PPAR G") OR TI(ppar-g) OR AB,TI("peroxisome proliferator-activated receptor gamma") OR TI("peroxisome proliferator-activated receptor G") OR TI("Peroxisome Proliferator-Activated Nuclear Receptor Gamma") OR TI("Peroxisome Proliferator Activated Receptor gamma") OR (TI("Peroxisome Proliferative Activated Receptor") AND TI(gamma)) OR TI("PPARgamma3") OR TI(PPARgamma2) OR TI(mPPARgamma1) OR TI(mPPARgamma2) OR TI(PPARG1) OR TI(PPARG2) OR TI(PPARG3)) AND YR(1990/01/01-2017/10/30)

6. Google Scholar Search Strategy (Hand Searching)

allintitle: “type 2 diabetes mellitus” + PPARG

allintitle: T2DM + PPARG

Key Journals (Hand Searching)

1. Diabetes

2. Diabetologia
