# Supplementary material for: PPARG (Pro12Ala) genetic variant and risk of T2DM: a systematic review and meta-analysis
Source: Sci Rep. 2020 Jul 29;10:12764. doi: 10.1038/s41598-020-69363-7 (PMC7391673; doi:10.1038/s41598-020-69363-7)
Supplement: Supplementary file 10 — Supplementary Information 10. [file 41598_2020_69363_MOESM10_ESM.pdf]


PPARG (Pro12Ala) genetic variant and risk of T2DM: a systematic review and meta-analysis

Negar Sarhangi 1, Farshad Sharifi 2, Leila Hashemian 3, Maryam Hassani Doabsari 3, Katayoun Heshmatzad 3, Marzieh Rahbaran 3, Seyed Hamid Jamaldini 3, Hamid Reza Aghaei Meybodi 1, 4, Mandana Hasanzad 1,3,*

1 Personalized Medicine Research Center, Endocrinology and Metabolism Clinical Sciences Institute, Tehran University of Medical Sciences, Tehran 1411413137, Iran
2 Elderly Health Research Center, Endocrinology and Metabolism Population Sciences Institute, Tehran University of Medical Sciences, Tehran 1411413137, Iran
3 Medical Genomics Research Center, Tehran Medical Sciences, Islamic Azad University, Tehran 1916893813, Iran
4 Endocrinology and Metabolism Research Center, Endocrinology and Metabolism Clinical Sciences Institute, Tehran University of Medical Sciences, Tehran 1411413137, Iran

```
 name:  <unnamed>
       log:  E:\My Documents\Sarhangi study\New\Analysis\Untitled.log
  log type:  text
 opened on:   8 Sep 2019, 09:34:52

. do "C:\Users\241143~1\AppData\Local\Temp\STD29000000.tmp"

. metan logOR selog_OR,  eform  xlabel(0.2,0.4,0.6,0.8) random label(namevar= F
> irstAuthor)

           Study     |     ES    [95% Conf. Interval]     % Weight
---------------------+---------------------------------------------------
Majithia et.al (2014 |  0.850       0.777     0.930         20.28
Tai et.al (2004) (A) |  0.037       0.033     0.042         20.26
Tai et.al (2004) (B) |  0.032       0.026     0.040         20.21
Tai et.al (2004) (C) |  0.119       0.102     0.139         20.25
Soriguer et.al (2006 |  0.300       0.122     0.740         19.01
---------------------+---------------------------------------------------
D+L pooled ES        |  0.128       0.027     0.612        100.00
---------------------+---------------------------------------------------

  Heterogeneity chi-squared = 1973.73 (d.f. = 4) p = 0.000
  I-squared (variation in ES attributable to heterogeneity) =  99.8%
  Estimate of between-study variance Tau-squared =  3.1454

  Test of ES=1 : z=   2.57 p = 0.010

.
end of do-file

. restore

. preserve
```

```
. keep if GeneticModels=="Additive"
(9 observations deleted)

. do "C:\Users\241143~1\AppData\Local\Temp\STD29000000.tmp"

. metan logOR selog_OR,  eform  xlabel(0.2,0.4,0.6,0.8) random label(namevar= F
> irstAuthor)
variable logOR not found
r(111);

end of do-file

r(111);

. restore

. restore
nothing to restore
r(622);

. do "C:\Users\241143~1\AppData\Local\Temp\STD29000000.tmp"

. gen logOR=log(OR)

. gen log_low_band=log( Low_OR)

. gen log_up_band=log( Up_OR )

. gen selog_OR= (log_up_band- logOR)/1.96

.
end of do-file

. save "E:\My Documents\Sarhangi study\New\Analysis\OR.dta", replace
file E:\My Documents\Sarhangi study\New\Analysis\OR.dta saved

. preserve

. keep if GeneticModels=="Additive"
(9 observations deleted)

. do "C:\Users\241143~1\AppData\Local\Temp\STD29000000.tmp"

. metan logOR selog_OR,  eform  xlabel(0.2,0.4,0.6,0.8) random label(namevar= F
> irstAuthor)

           Study     |     ES    [95% Conf. Interval]     % Weight
---------------------+---------------------------------------------------
Chan et.al (2013) (A |  0.620       0.343     1.120         64.39
Chan et.al (2013) (B |  0.590       0.151     2.310         12.09
Chan et.al (2013) (C |  0.270       0.078     0.930         14.72
Chan et.al (2013) (D |  0.210       0.042     1.040          8.80
---------------------+---------------------------------------------------
D+L pooled ES        |  0.496       0.308     0.797        100.00
---------------------+---------------------------------------------------

  Heterogeneity chi-squared =   2.65 (d.f. = 3) p = 0.449
  I-squared (variation in ES attributable to heterogeneity) =   0.0%
  Estimate of between-study variance Tau-squared =  0.0000
```

```
  Test of ES=1 : z=   2.90 p = 0.004

.
end of do-file

. do "C:\Users\241143~1\AppData\Local\Temp\STD29000000.tmp"

. metan logOR selog_OR,  eform  xlabel(0.2,0.4,0.6,0.8) fix label(namevar= Firs
> tAuthor)

           Study     |     ES    [95% Conf. Interval]     % Weight
---------------------+---------------------------------------------------
Chan et.al (2013) (A |  0.620       0.343     1.120         64.39
Chan et.al (2013) (B |  0.590       0.151     2.310         12.09
Chan et.al (2013) (C |  0.270       0.078     0.930         14.72
Chan et.al (2013) (D |  0.210       0.042     1.040          8.80
---------------------+---------------------------------------------------
I-V pooled ES        |  0.496       0.308     0.797        100.00
---------------------+---------------------------------------------------

  Heterogeneity chi-squared =   2.65 (d.f. = 3) p = 0.449
  I-squared (variation in ES attributable to heterogeneity) =   0.0%

  Test of ES=1 : z=   2.90 p = 0.004
option fix not allowed
r(198);

end of do-file

r(198);

. do "C:\Users\241143~1\AppData\Local\Temp\STD29000000.tmp"

. metan logOR selog_OR,  eform  xlabel(0.2,0.4,0.6,0.8) label(namevar= FirstAut
> hor)

           Study     |     ES    [95% Conf. Interval]     % Weight
---------------------+---------------------------------------------------
Chan et.al (2013) (A |  0.620       0.343     1.120         64.39
Chan et.al (2013) (B |  0.590       0.151     2.310         12.09
Chan et.al (2013) (C |  0.270       0.078     0.930         14.72
Chan et.al (2013) (D |  0.210       0.042     1.040          8.80
---------------------+---------------------------------------------------
I-V pooled ES        |  0.496       0.308     0.797        100.00
---------------------+---------------------------------------------------

  Heterogeneity chi-squared =   2.65 (d.f. = 3) p = 0.449
  I-squared (variation in ES attributable to heterogeneity) =   0.0%

  Test of ES=1 : z=   2.90 p = 0.004

.
end of do-file

. restore

. preserve
```

```
. keep if GeneticModels=="Dominanat"
(9 observations deleted)

. do "C:\Users\241143~1\AppData\Local\Temp\STD29000000.tmp"

. metan logOR selog_OR,  eform  xlabel(0.2,0.4,0.6,0.8) random label(namevar= F
> irstAuthor)

           Study     |     ES    [95% Conf. Interval]     % Weight
---------------------+---------------------------------------------------
Chan et.al (2013) (A |  0.600       0.321     1.120         61.24
Chan et.al (2013) (B |  0.590       0.151     2.310         14.76
Chan et.al (2013) (C |  0.190       0.045     0.810         13.14
Chan et.al (2013) (D |  0.210       0.042     1.040         10.86
---------------------+---------------------------------------------------
D+L pooled ES        |  0.459       0.269     0.784        100.00
---------------------+---------------------------------------------------

  Heterogeneity chi-squared =   3.17 (d.f. = 3) p = 0.367
  I-squared (variation in ES attributable to heterogeneity) =   5.3%
  Estimate of between-study variance Tau-squared =  0.0204

  Test of ES=1 : z=   2.85 p = 0.004

.
end of do-file

. do "C:\Users\241143~1\AppData\Local\Temp\STD29000000.tmp"

. metan logOR selog_OR,  eform  xlabel(0.2,0.4,0.6,0.8) label(namevar= FirstAut
> hor)

           Study     |     ES    [95% Conf. Interval]     % Weight
---------------------+---------------------------------------------------
Chan et.al (2013) (A |  0.600       0.321     1.120         64.66
Chan et.al (2013) (B |  0.590       0.151     2.310         13.52
Chan et.al (2013) (C |  0.190       0.045     0.810         11.98
Chan et.al (2013) (D |  0.210       0.042     1.040          9.84
---------------------+---------------------------------------------------
I-V pooled ES        |  0.470       0.285     0.777        100.00
---------------------+---------------------------------------------------

  Heterogeneity chi-squared =   3.17 (d.f. = 3) p = 0.367
  I-squared (variation in ES attributable to heterogeneity) =   5.3%

  Test of ES=1 : z=   2.95 p = 0.003

.
end of do-file

. restore

. *(1 variable, 1 observation pasted into data editor)

. *(1 variable, 0 observation pasted into data editor)

. replace parketal2012 = "Park et.al (2012)" in 14
(1 real change made)
```

```
. replace OR = 0.4 in 14
(1 real change made)

. replace Low_OR = 0.18 in 14
(1 real change made)

. replace Up_OR = 0.91 in 14
(1 real change made)

. replace Ethnicity = "Korean" in 14
(1 real change made)

. replace HWE = 0.796 in 14
variable HWE was byte now float
(1 real change made)

. drop log_low_band log_up_band selog_OR

. drop logOR

. do "C:\Users\241143~1\AppData\Local\Temp\STD29000000.tmp"

. gen logOR=log(OR)

. gen log_low_band=log( Low_OR)

. gen log_up_band=log( Up_OR )

. gen selog_OR= (log_up_band- logOR)/1.96

. metan logOR selog_OR,  eform  xlabel(0.2,0.4,0.6,0.8) random label(namevar= F
> irstAuthor)
Variable FirstAuthor not defined

. metan logOR selog_OR,  eform  xlabel(0.2,0.4,0.6,0.8) label(namevar= FirstAut
> hor)
Variable FirstAuthor not defined

.
end of do-file

. rename parketal2012 FirstAuthor

. label variable FirstAuthor "FirstAuthor"

. save "E:\My Documents\Sarhangi study\New\Analysis\OR.dta", replace
file E:\My Documents\Sarhangi study\New\Analysis\OR.dta saved

. do "C:\Users\241143~1\AppData\Local\Temp\STD29000000.tmp"

. metan logOR selog_OR,  eform  xlabel(0.2,0.4,0.6,0.8) random label(namevar= F
> irstAuthor)

           Study     |     ES    [95% Conf. Interval]     % Weight
---------------------+---------------------------------------------------
Majithia et.al (2014 |  0.850       0.777     0.930          7.76
Tai et.al (2004) (A) |  0.037       0.033     0.042          7.76
Tai et.al (2004) (B) |  0.032       0.026     0.040          7.73
Tai et.al (2004) (C) |  0.119       0.102     0.139          7.75
```

```
Soriguer et.al (2006 |  0.300       0.122     0.740          7.24
Chan et.al (2013) (A |  0.620       0.343     1.120          7.53
Chan et.al (2013) (B |  0.590       0.151     2.310          6.67
Chan et.al (2013) (C |  0.270       0.078     0.930          6.84
Chan et.al (2013) (D |  0.210       0.042     1.040          6.33
Chan et.al (2013) (A |  0.600       0.321     1.120          7.51
Chan et.al (2013) (B |  0.590       0.151     2.310          6.67
Chan et.al (2013) (C |  0.190       0.045     0.810          6.55
Chan et.al (2013) (D |  0.210       0.042     1.040          6.33
Park et.al (2012)    |  0.400       0.176     0.910          7.33
---------------------+---------------------------------------------------
D+L pooled ES        |  0.248       0.097     0.633        100.00
---------------------+---------------------------------------------------

  Heterogeneity chi-squared = 2002.57 (d.f. = 13) p = 0.000
  I-squared (variation in ES attributable to heterogeneity) =  99.4%
  Estimate of between-study variance Tau-squared =  2.9408

  Test of ES=1 : z=   2.92 p = 0.004

. metan logOR selog_OR,  eform  xlabel(0.2,0.4,0.6,0.8) label(namevar= FirstAut
> hor)

           Study     |     ES    [95% Conf. Interval]     % Weight
---------------------+---------------------------------------------------
Majithia et.al (2014 |  0.850       0.777     0.930         47.82
Tai et.al (2004) (A) |  0.037       0.033     0.042         24.08
Tai et.al (2004) (B) |  0.032       0.026     0.040          7.77
Tai et.al (2004) (C) |  0.119       0.102     0.139         16.03
Soriguer et.al (2006 |  0.300       0.122     0.740          0.47
Chan et.al (2013) (A |  0.620       0.343     1.120          1.11
Chan et.al (2013) (B |  0.590       0.151     2.310          0.21
Chan et.al (2013) (C |  0.270       0.078     0.930          0.25
Chan et.al (2013) (D |  0.210       0.042     1.040          0.15
Chan et.al (2013) (A |  0.600       0.321     1.120          0.99
Chan et.al (2013) (B |  0.590       0.151     2.310          0.21
Chan et.al (2013) (C |  0.190       0.045     0.810          0.18
Chan et.al (2013) (D |  0.210       0.042     1.040          0.15
Park et.al (2012)    |  0.400       0.176     0.910          0.57
---------------------+---------------------------------------------------
I-V pooled ES        |  0.220       0.207     0.234        100.00
---------------------+---------------------------------------------------

  Heterogeneity chi-squared = 2002.57 (d.f. = 13) p = 0.000
  I-squared (variation in ES attributable to heterogeneity) =  99.4%

  Test of ES=1 : z=  47.74 p = 0.000

.
end of do-file

. save "E:\My Documents\Sarhangi study\New\Analysis\OR.dta", replace
file E:\My Documents\Sarhangi study\New\Analysis\OR.dta saved

. preserve

. keep if GeneticModels=="Dominanat"
(9 observations deleted)
```

```
. do "C:\Users\241143~1\AppData\Local\Temp\STD29000000.tmp"

. metan logOR selog_OR,  eform  xlabel(0.2,0.4,0.6,0.8) random label(namevar= F
> irstAuthor)

           Study     |     ES    [95% Conf. Interval]     % Weight
---------------------+---------------------------------------------------
Chan et.al (2013) (A |  0.600       0.321     1.120         47.10
Chan et.al (2013) (B |  0.590       0.151     2.310          9.85
Chan et.al (2013) (C |  0.190       0.045     0.810          8.73
Chan et.al (2013) (D |  0.210       0.042     1.040          7.17
Park et.al (2012)    |  0.400       0.176     0.910         27.16
---------------------+---------------------------------------------------
D+L pooled ES        |  0.450       0.293     0.691        100.00
---------------------+---------------------------------------------------

  Heterogeneity chi-squared =   3.28 (d.f. = 4) p = 0.513
  I-squared (variation in ES attributable to heterogeneity) =   0.0%
  Estimate of between-study variance Tau-squared =  0.0000

  Test of ES=1 : z=   3.65 p = 0.000

. metan logOR selog_OR,  eform  xlabel(0.2,0.4,0.6,0.8) label(namevar= FirstAut
> hor)

           Study     |     ES    [95% Conf. Interval]     % Weight
---------------------+---------------------------------------------------
Chan et.al (2013) (A |  0.600       0.321     1.120         47.10
Chan et.al (2013) (B |  0.590       0.151     2.310          9.85
Chan et.al (2013) (C |  0.190       0.045     0.810          8.73
Chan et.al (2013) (D |  0.210       0.042     1.040          7.17
Park et.al (2012)    |  0.400       0.176     0.910         27.16
---------------------+---------------------------------------------------
I-V pooled ES        |  0.450       0.293     0.691        100.00
---------------------+---------------------------------------------------

  Heterogeneity chi-squared =   3.28 (d.f. = 4) p = 0.513
  I-squared (variation in ES attributable to heterogeneity) =   0.0%

  Test of ES=1 : z=   3.65 p = 0.000

.
end of do-file

. restore

. tab FirstAuthor

                            FirstAuthor |      Freq.     Percent        Cum.
----------------------------------------+-----------------------------------
Chan et.al (2013) (A) (European-Ameri.. |          2       14.29       14.29
Chan et.al (2013) (B) (African-Americ.. |          2       14.29       28.57
Chan et.al (2013) (C) (Hispanic-Ameri.. |          2       14.29       42.86
 Chan et.al (2013) (D) (Asian-American) |          2       14.29       57.14
                  Majithia et.al (2014) |          1        7.14       64.29
                      Park et.al (2012) |          1        7.14       71.43
                 Soriguer et.al (2006)  |          1        7.14       78.57
         Tai et.al (2004) (A) (Chinese) |          1        7.14       85.71
           Tai et.al (2004) (B) (Malay) |          1        7.14       92.86
```

```
          Tai et.al (2004) (C) (Indian) |          1        7.14      100.00
----------------------------------------+----------------------------------
                                  Total |         14      100.00

. exit, clear
```

Reference: Sterne JA, Bradburn MJ, Egger M. Meta?Analysis in Stata�. Systematic reviews in health care: meta-analysis in context. 2001 Jan 1:347-69.
