# Supplementary material for: PPARG (Pro12Ala) genetic variant and risk of T2DM: a systematic review and meta-analysis
Source: Sci Rep. 2020 Jul 29;10:12764. doi: 10.1038/s41598-020-69363-7 (PMC7391673; doi:10.1038/s41598-020-69363-7)
Supplement: Supplementary file 12 — Supplementary Information 12. [file 41598_2020_69363_MOESM12_ESM.pdf]

PPARG (Pro12Ala) genetic variant and risk of T2DM: a systematic review and meta-analysis

Negar Sarhangi [1], Farshad Sharifi [2], Leila Hashemian [3], Maryam Hassani Doabsari [3], Katayoun Heshmatzad [3], Marzieh Rahbaran [3], Seyed Hamid Jamaldini [3], Hamid Reza Aghaei Meybodi [1, 4], Mandana Hasanzad [1,3,*]

[1] Personalized Medicine Research Center, Endocrinology and Metabolism Clinical Sciences Institute, Tehran University of Medical Sciences, Tehran 1411413137, Iran

[2] Elderly Health Research Center, Endocrinology and Metabolism Population Sciences Institute, Tehran University of Medical Sciences, Tehran 1411413137, Iran

[3] Medical Genomics Research Center, Tehran Medical Sciences, Islamic Azad University, Tehran 1916893813, Iran

[4] Endocrinology and Metabolism Research Center, Endocrinology and Metabolism Clinical Sciences Institute, Tehran University of Medical Sciences, Tehran 1411413137, Iran

First author (year)/ref	Title	Reason for exclusion
Zmyslowska (2014) (1)	The Pro12Ala PPARg2 gene polymorphism involves residual C-peptide secretion and BMI in type 1 diabetes	Study on Type 1 diabetes
Johansen A (2006) (2)	IRS1, KCNJ11, PPARgamma2 and HNF-1alpha: do amino acid polymorphisms in these candidate genes support a shared etiology between type 1 and type 2 diabetes?	To estimate the impact of PPARg2 Pro12Ala in a Danish population of T1DM families
Wu LSH (2009) (3)	Association and interaction analyses of genetic variants in ADIPOQ, ENPP1, GHSR, PPARγ and TCF7L2 genes for diabetic nephropathy in a Taiwanese population with type 2 diabetes	Diabetic Nephropathy (Diabetes Complication)
Zhang Y (2015) (4)	The gene polymorphisms of UCP1 but not PPAR gamma and TCF7L2 are associated with diabetic retinopathy in Chinese type 2 diabetes mellitus cases	Diabetic Retinopathy (Diabetes Complication)
Pollex RL (2007) (5)	Peroxisome proliferator-activated receptor gamma polymorphism Pro12Ala is associated with nephropathy in type 2 diabetes	Diabetic Nephropathy (Diabetes Complication)
Franck N (2012) (6)	Cardiovascular risk factors related to the PPARγ Pro12Ala polymorphism in patients with type 2 diabetes are gender dependent	Study on pro12ala in Cardiovascular just in T2D patients by comparison based on gender. (Diabetes Complications)
Herrmann SM (2002) (7)	Peroxisome proliferator-activated receptor-gamma 2 polymorphism Pro12Ala is associated with nephropathy in type 2 diabetes - The Berlin Diabetes Mellitus (BeDiaM) Study	Investigated whether PPAR-G2 Pro12Ala was associated with micro-vascular complications of type 2 diabetes. The study has not control group (Diabetes Complications)
Lapice E (2015) (8)	The PPARgamma2 Pro12Ala variant is protective against progression of nephropathy in people with type 2 diabetes	To evaluates the association between the Pro12Ala polymorphism of PPARγ2 and the progression of albuminuria and decay in glomerular filtration rate (GFR) in type 2 diabetes (Diabetes Complications)
Kaur N (2017) (9)	Association analysis of PPARgamma (p. Pro12Ala) polymorphism with type 2 diabetic retinopathy in patients from north India	The control group consists of T2D without retinopathy. As control group do not match with my control according to criteria (Diabetes Complications)

Malecki MT (2008) (10)	Alanine variant of the Pro12Ala polymorphism of the PPARgamma gene might be associated with decreased risk of diabetic retinopathy in type 2 diabetes	Diabetic Retinopathy (Diabetes Complications)
Petrovic MG (2005) (11)	Gly482Ser polymorphism of the peroxisome proliferator-activated receptor-gamma coactivator-1 gene might be a risk factor for diabetic retinopathy in Slovene population (Caucasians) with type 2 diabetes and the Pro12Ala polymorphism of the PPAR gamma gene is not	Diabetic Retinopathy (Diabetes Complication)
Maria Luiza Caramori (2003) /(12)	The Human Peroxisome Proliferator–Activated Receptor □2 (PPAR□2) Pro12Ala Polymorphism Is Associated With Decreased Risk of Diabetic Nephropathy in Patients With Type 2 Diabetes	Diabetic Nephropathy (Diabetes Complications)
Ereqat S (2009) (13)	Impact of the pro12Ala polymorphism of the PPAR-gamma 2 gene on metabolic and clinical characteristics in the palestinian type 2 diabetic patients	Metabolic and clinical characteristics
Hara M (2012) (14)	Effect of the PPARG2 Pro12Ala polymorphism and clinical risk factors for diabetes mellitus on HbA1c in the Japanese general population	Examine if carrying the Ala allele of PPARG2 was inversely associated with HbA1c and if this association modified the effects of known clinical risk factors, family history of diabetes, and their interactions (Metabolic Traits)
Stumvoll M (2002) (15)	Interaction effect between common polymorphisms in PPARgamma2 (Pro12Ala) and insulin receptor substrate 1 (Gly972Arg) on insulin sensitivity	Metabolic Traits
Stryjecki C (2016) (16)	Association between PPAR-gamma 2 Pro12Ala genotype and insulin resistance is modified by circulating lipids in Mexican children	Metabolic Traits
Black MH (2015) (17)	Variation in PPARG is associated with longitudinal change in insulin resistance in Mexican Americans at risk for type 2 diabetes	Metabolic Traits
Tellechea ML (2009) (18)	Pro12Ala polymorphism of the peroxisome proliferatoractivated receptor-gamma gene is associated with metabolic syndrome and surrogate measures of insulin resistance in healthy men: interaction with smoking status	Metabolic Traits
Ahluwalia M (2002) (19)	The influence of the Pro12Ala mutation of the PPAR-gamma receptor gene on metabolic and clinical characteristics in treatment-naive patients with type 2 diabetes	Metabolic Traits
Zietz B (2002) (20)	Pro12Ala polymorphism in the peroxisome proliferator-activated receptor-gamma2 (PPARgamma2) is associated with higher levels of total cholesterol and LDL-cholesterol in male Caucasian type 2 diabetes patients	Determine the possible association of Pro12Ala polymorphism with metabolic

		parameters and diabetic retinopathy in type 2 diabetes patients<br>(Metabolic Traits)
Nasereddin A (2009) (21)	Impact of the pro12Ala polymorphism of the PPAR-gamma 2 gene on metabolic and clinical characteristics in the palestinian type 2 diabetic patients	Investigate the relationship between P12A polymorphism and blood pressure, BMI and other related metabolic parameters in type 2 diabetic.<br>(Metabolic Traits)
Kadowaki T (2002) (22)	The role of PPARg in high-fat diet-induced obesity and insulin resistance	Insulin resistance<br>(Metabolic Traits)
Ostergard T (2005) (23)	Influence of the PPAR-γ2 Pro12Ala and ACE I/D Polymorphisms on Insulin Sensitivity and Training Effects in Healthy Offspring of Type 2 Diabetic Subjects	Insulin sensitivity<br>(Metabolic Traits)
Villegas R (2014) (24)	Genetic variation in the peroxisome proliferator-activated receptor (PPAR) and peroxisome proliferator-activated receptor gamma co-activator 1 (PGC1) gene families and type 2 diabetes	Not original article<br>Other SNP
Bonnycastle LL (2006) (25)	Common variants in maturity-onset diabetes of the young genes contribute to risk of type 2 diabetes in Finns	Other genes
Zhu S (2009) (26)	Evaluation of the association between the PPARGC1A genetic polymorphisms and type 2 diabetes in Han Chinese population	Study on association between PGC-1α gene and type 2 diabetes<br>(Other genes)
Zhang SL (2007) (27)	Association between peroxisome proliferator-activated receptor-gamma coactivator-1alpha gene polymorphisms and type 2 diabetes in southern Chinese population: role of altered interaction with myocyte enhancer factor 2C	Study on association between PGC-1α gene and type 2 diabetes<br>(Other genes)
Shokouhi S (2015) (28)	Association between PGC-1alpha gene polymorphisms and type 2 diabetes risk: a case-control study of an Iranian population	Study on association between PGC-1α gene and type 2 diabetes<br>(Other genes)
Sharma R (2018) (29)	Association of PGC-1α gene with type 2 diabetes in three unrelated endogamous groups of North-West India (Punjab): a case-control and meta-analysis study	Study on association between PGC-1α gene and type 2 diabetes<br>(Other genes)
Vasseur F (2005) (30)	Hypoadiponectinaemia and high risk of type 2 diabetes are associated with adiponectin-encoding (ACDC) gene promoter variants in morbid obesity: evidence for a role of ACDC in diabesity	The genotype distribution of Pro12Ala are not specified in the diabetes and non-diabetes group according to my criteria<br>(Did not have relevant data)
West NA (2010) (31)	The PPAR-gamma Pro12Ala polymorphism and risk of cognitive impairment in a longitudinal study	Cognitive Impairment<br>(Did not have relevant data)

Thamer C (2002) (32)	Evidence for greater oxidative substrate flexibility in male carriers of the Pro 12 Ala polymorphism in PPARgamma2	Examined respiratory quotient (RQ), lipid and carbohy-drate oxidation rates in healthy, normal weight subjects with and without the Pro 12 Ala polymorphism in PPARg2 in the fast-ing state and during a hyperinsulinaemic euglycaemic clamp. (Did not have relevant data)
Tan GD (2006) (33)	The in vivo effects of the Pro12Ala PPARgamma2 polymorphism on adipose tissue NEFA metabolism: the first use of the Oxford Biobank	Study on adipose tissue metabolism and cardiovascular risk factors (Other Reasons)
Takata N (2004) (34)	Pro12Ala substitution in peroxisome proliferator-activated receptor gamma 2 is associated with low adiponectin concentrations in young Japanese men	Determine whether PPARG Pro12Ala substitution is associated with low serum adiponectin concentrations in young healthy adults (Did not have relevant data)
Stefanski A (2006) (35)	Lack of association between the Pro12Ala polymorphism in PPAR-gamma 2 gene and body weight changes, insulin resistance and chronic diabetic complications in obese patients with type 2 diabetes	Study on body weight and its changes, differences in insulin resistance and insulin secretion, secondary failure of oral hypoglyce-mic agents and incidence of diabetic complications in obese patients with long-lasting type 2 diabetes. (Did not have relevant data)
Yates T (2015) (36)	Effect of the PPARG2 Pro12Ala Polymorphism on Associations of Physical Activity and Sedentary Time with Markers of Insulin Sensitivity in Those with an Elevated Risk of Type 2 Diabetes	Associations of sedentary behavior and moderate-to-vigorous intensity physical activity (MVPA) with common measures of insulin sensitivity (Did not have relevant data)
Bendlova B (2008) (37)	PPAR gamma 2 Pro12Ala polymorphism in relation to free fatty acids concentration and composition in lean healthy Czech individuals with and without family history of diabetes type 2	Association with free fatty acids concentration (Did not have relevant data)
Verier C (2010) (38)	Breast-feeding modulates the influence of the peroxisome proliferator-activated receptor-gamma (PPARG2) Pro12Ala polymorphism on adiposity in adolescents: The Healthy Lifestyle in Europe by Nutrition in Adolescence (HELENA) cross-sectional study	Assess the influence of the PPARG2 Pro12Ala polymorphism on adiposity markers in adolescents (Did not have relevant data)
Namvaran F (2011) (39)	Genotyping of peroxisome proliferator-activated receptor gamma (PPAR-γ) polymorphism (pro12ala) in Iranian population	Investigation of frequency of Pro12Ala polymorphism in PPAR-γ in healthy Iranian population to compare with other populations (Did not have relevant data)

Lapice E (2016) (40)	The combination of UCP3-55CT and PPARgamma2Pro12Ala polymorphisms affects BMI and substrate oxidation in two diabetic populations	Evaluate the combined contribution of UCP3-55CT and PARγ2 Pro12Ala polymorphisms as correlates of BMI, energy expenditure (REE) and substrate oxidation in people with type 2 diabetes. Not have control group (Did not have relevant data)
Eftychi C (2004) (41)	Analysis of the Type 2 Diabetes-Associated Single Nucleotide Polymorphisms in the Genes IRS1, KCNJ11, and PPARG2 in Type 1 Diabetes	(Did not have relevant data)
Tschritter O (2003) (42)	Increased insulin clearance in peroxisome proliferator-activated receptor gamma (2) Pro12Ala	Association of pro12ala with the mechanism of T2DM not risk of T2DM (Did not have relevant data)
Poulsen P (2003) (43)	Impact of two common polymorphisms in the PPAR gamma gene on glucose tolerance and plasma insulin profiles in monozygotic and dizygotic twins - Thrifty genotype, thrifty phenotype, or both?	According to our inclusion criteria, the samples of this study include MZ and DZ twins. (Did not have relevant data)
Lamri A (2012) (44)	Dietary fat intake and polymorphisms at the PPARG locus modulate BMI and type 2 diabetes risk in the D.E.S.I.R. prospective study	Study potential interactions between Pro12Ala polymorphisms and dietary fat intake (consumption of meat, fish, pork products, fried foods, butter, cheese and other dairy products) on T2D and BMI, in a prospective cohort. (Other Reasons)
Estivalet AAF (2011) (45)	D2 Thr92Ala and PPARγ2 Pro12Ala Polymorphisms Interact in the Modulation of Insulin Resistance in Type 2 Diabetic Patients	evaluate the potential synergistic effect of D2 Tr92Ala and PPARγ2 Pro12Ala polymorphisms on IR and related charac-teristics in a sample of white DM2 patients. (Did not have relevant data)
Ruchat SM (2009) (46)	Evidence for Interaction between PPARG Pro12Ala and PPARGC1A Gly482Ser Polymorphisms in Determining Type 2 Diabetes Intermediate Phenotypes in Overweight Subjects	T2DM intermediate phenotypes Subjects who underwent a 75 g oral glucose tolerant test (OGTT) (Did not have relevant data)
Lee BC (2006) (47)	Peroxisome proliferator-activated receptor-gamma 2 Pro12Ala polymorphism is associated with reduced risk for ischemic stroke with type 2 diabetes	ischemic stroke (Did not have relevant data)

Kozarova M (2010) (48)	Relationship of five type 2 diabetes candidate gene polymorphisms to the age at diagnosis of diabetes in the Slovakian population	Examine the effect of Pro12Ala on the age at diagnosis of T2D just in T2D patients<br>(Did not have relevant data)
Okazawa H (1997) (49)	No Coding Mutations Are Detected in the Peroxisome Proliferator-Activated Receptor-smgamma. gif (938 bytes) Gene in Japanese Patients with Lipoatrophic Diabetes	Lipoatrophic Diabetes<br>(Did not have relevant data)
Vigouroux C (1998) (50)	Human peroxisome proliferator-activated receptor-gamma2: genetic mapping, identification of a variant in the coding sequence, and exclusion as the gene responsible for lipoatrophic diabetes	Lipoatrophic Diabetes<br>(Did not have relevant data)
Andrulionyte L (2004) (51)	Common polymorphisms of the PPAR-gamma 2 (Pro12Ala) and PGC-1 alpha (Gly482Ser) genes are associated with the conversion from impaired glucose tolerance to type 2 diabetes in the STOP-NIDDM trial	Investigated the effects of the (PPAR- 2; Pro12Ala) on the conversion from impaired glucose tolerance to type 2 diabetes in participants in the STOP-NIDDM trial.<br>Effect of acarbose in the prevention of type 2 diabetes.<br>(Clinical Trial Study)
Andrulionyte L (2006) (52)	Single nucleotide polymorphisms of PPARD in combination with the Gly482Ser substitution of PGC-1A and the Pro12Ala substitution of PPARG2 predict the conversion from impaired glucose tolerance to type 2 diabetes: the STOP-NIDDM trial	Investigated the effects of the (PPAR- 2; Pro12Ala) on the conversion from impaired glucose tolerance (IGT) to type 2 diabetes in the STOP-NIDDM trial.<br>(Clinical Trial Study)
Kilpelainen TO (2008) (53)	SNPs in PPARG associate with type 2 diabetes and interact with physical activity	Clinical Trial<br>From DPS (cohort study), clinical trial was done.
Lindi VI (2002) (54)	Association of the Pro12Ala polymorphism in the PPAR-gamma 2 gene with 3-year incidence of type 2 diabetes and body weight change in the Finnish Diabetes Prevention Study	Subjects are IGT not T2D<br>Intervention was done<br>Clinical Trial
Florez JC (2007) (55)	Effects of the type 2 diabetes-associated PPARG P12A polymorphism on progression to diabetes and response to troglitazone	Clinical Trial<br>Subjects are IGT
Chung-Jen Y (1997) (56)	Molecular scanning of the human peroxisome proliferator activated receptor γ (hPPARγ) gene in diabetic Caucasians: Identification of a Pro12Ala PPARγ2 missense mutation	Case-series<br>Did not have control group<br>Did not have sufficient data
Scacchi R (2007) (57)	An analysis of peroxisome proliferator-activated receptor gamma (PPAR-gamma 2) Pro12Ala polymorphism distribution and prevalence of type 2 diabetes mellitus (T2DM) in world populations in relation to dietary habits	Case-series<br>Not have control group<br>Not response from author to receive data

Bego T (2011) (58)	Association of PPARG and LPIN1 gene polymorphisms with metabolic syndrome and type 2 diabetes	Case-series<br>Not sufficient data<br>Have not control group and investigation in T2DM patients
Celi FS (2002) (59)	The role of peroxisome proliferator-activated receptor gamma in diabetes and obesity	Not a study- Review article
Tonjes A (2007) (60)	The role of the Pro12Ala polymorphism in peroxisome proliferator-activated receptor gamma in diabetes risk	Not a study- Review article
Radha V (2007) (61)	Genetic predisposition to type 2 diabetes among Asian Indians	Review
Stumvoll M (2002) (62)	The Peroxisome Proliferator–Activated Receptor-□2 Pro12Ala Polymorphism	Review
Dong C (2015) (63)	Role of peroxisome proliferator-activated receptors gene polymorphisms in type 2 diabetes and metabolic syndrome	Mini Review
Tong JY (2012) (64)	[Relationship between PPARgamma2 Pro12Ala polymorphism and type 2 diabetes mellitus in Chinese Han population: a Meta-analysis]	Meta-Analysis
Guo WL (2011) (65)	[Meta-analysis of the association of Pro12Ala polymorphism of peroxisome proliferator activated receptor gamma gene with type 2 diabetes in Chinese Han population]	Meta-analysis
Huguenin GVB (2010) (66)	The Ala allele in the PPAR-g2 gene is associated with reduced risk of type 2 diabetes mellitus in Caucasians and improved insulin sensitivity in overweight subjects	Meta-analysis
Ludovico O (2007) (67)	Heterogeneous Effect of Peroxisome Proliferator-activated Receptor γ2 Ala12 Variant on Type 2 Diabetes Risk	Meta-Analysis
Altshuler D (2000) (68)	The common PPARγ Pro12Ala polymorphism is associated with decreased risk of type 2 diabetes	Letter
Deeb SS (1998) (69)	A Pro12Ala substitution in PPARγ2 associated with decreased receptor activity, lower body mass index and improved insulin sensitivity	Letter

Barroso I (1999) (70)	Dominant negative mutations in human PPARγ associated with severe insulin resistance, diabetes mellitus and hypertension	Letter
Wakil SM (2006) (71)	The Peroxisome Proliferator–Activated Receptor-γ2 P12A Polymorphism and Type 2 Diabetes in an Arab Population	Letter
Nelson TL (2007) (72)	Association of the peroxisome proliferator–activated receptor g gene with type 2 diabetes mellitus varies by physical activity among non-Hispanic whites from Colorado	Familial Study
Black MH (2008) (73)	Evidence of interaction between PPARG2 and HNF4A contributing to variation in insulin sensitivity in Mexican Americans	Familial Study
Lyssenko V (2008) (74)	Clinical risk factors, DNA variants, and the development of type 2 diabetes	Cohort
Park SE (2012) (75)	Impact of common type 2 diabetes risk gene variants on future type 2 diabetes in the non-diabetic population in Korea	Cohort
Lin Y (2010) (76)	Association study of genetic variants in eight genes/loci with type 2 diabetes in a Han Chinese population	Loci
Hu C (2009) (77)	PPARG, KCNJ11, CDKAL1, CDKN2A-CDKN2B, IDE-KIF11-HHEX, IGF2BP2 and SLC30A8 are associated with type 2 diabetes in a chinese population	Loci
Gamboa-Meléndez MA (2012) (78)	Contribution of common genetic variation to the risk of type 2 diabetes in the Mexican Mestizo population	Loci
Hanson RL (2015) (79)	Role of Established Type 2 Diabetes-Susceptibility Genetic Variants in a High Prevalence American Indian Population	Loci
Chauhan G (2010) (80)	Impact of common variants of PPARG, KCNJ11, TCF7L2, SLC30A8, HHEX, CDKN2A, IGF2BP2 and CDKAL1 on the risk of type 2 diabetes in 5164 Indians	Loci
Voight BF (2010) (81)	Twelve type 2 diabetes susceptibility loci identified through large-scale association analysis	GWAS
(82)	Diabetes Epidemiology; Pro12Ala variant of PPARG2 gene plus high intake of oleic acid is linked to type 2 diabetes	Report

2016 (83)	Nutritional and Metabolic Diseases and Conditions; New Type 2 Diabetes Study Results from National Research Council Described (The combination of UCP3-55CT and PPAR gamma 2Pro12Ala polymorphisms affects BMI and substrate oxidation in two diabetic populations)	News
Lapice E (2013) (84)	Comment on: Zhang et al. Peroxisome proliferator-activated receptor gamma polymorphism Pro12Ala is associated with nephropathy in type 2 diabetes: evidence from meta-analysis of 18 studies	Commentary
Shah AY (2016) (85)	Interplay of Pparγ2 and adrβ3 allelic polymorphism and metabolic factors conferring poor hemoglobin glycation and increased insulin resistance: A western indian study	Poster<br>(Meeting Abstract)
Osman NA (2013) (86)	The association between diabetic nephropathy and polymorphisms of PPAR PRO12ALA and CCR5 32 genes in type 2 diabetes	Study the association between polymorphisms of<br>both, the PPAR γ Pro12Ala and CCR5 δ 32 genes with the presence of diabetic nephropathy in Egyptian type 2 diabetic patients<br>(Meeting Abstract)
Yang YZ (2010) (87)	Correlation Analysis of Pro12A1a Polymorphism in PPAR gamma 2 Gene with Susceptibility to Diabetic Nephropathy in Type 2 Diabetes Mellitus	Meeting Abstract
Namvaran F (2011) (88)	PEROXISOME PROLIFERATOR-ACTIVATED RECEPTOR Gamma POLYMORPHISM IN IRANIAN POPULATION: RELATION WITH INSULIN RESISTANCE AND RESPONSE TO PIOGLITAZONE IN DIABETES TYPE 2	Meeting Abstract<br>Main article included
Mato EPM (2014) (89)	No Association between Pro12Ala polymorphism in Peroxisome Proliferator Activated Receptor Gamma 2 gene and type 2 diabetes in a Cameroonian population	Meeting Abstract
Watanabe R (2000) (90)	Association of the peroxisome proliferator-activated receptory-gamma 2) (PPAR-gamma 2) PRO12ALA variant with type 2 diabetes-related phenotypes in a large Finnish cohort	Meeting Abstract<br>No full-text
Andersen G (2000) (91)	The Pro12Ala polymorphism of the peroxisome proliferator-activated receptor-gamma 2 is not associated with type 2 diabetes	Meeting Abstract<br>No full-text
Erdos MR (2000) (92)	The PPAR-gamma 2 Pro12Ala variant: association with type 2 diabetes, trait differences, and interaction with the beta(3)-adrenergic receptor	No full-text<br>Meeting Abstract
Ek J (2001) (93)	Studies of the Pro12Ala polymorphism of the peroxisome proliferator-activated receptor-gamma2 (PPAR-gamma2) gene in relation to insulin sensitivity among glucose tolerant caucasians	No full-text

Donnarumma G (2006) (94)	Absence of association between Pro12Ala polymorphism of the PPARγ2 gene and diabetes	No full-text
Zhang A (2005) (95)	The influence of the Pro12Ala mutation of PPARgamma2 receptor gene on beta-cells restoration and insulin resistance in type 2 diabetes with hypertension	beta-cells restoration and insulin resistance<br>No full-text
Azab MM (2014) (96)	Peroxisome Proliferator Activated Receptor gamma 2 Pro12Ala Gene Polymorphism in Type 2 Diabetes and its Relationship with Diabetic Nephropathy	No full-text
Kawasaki I (2002) (97)	Impact of Prol2Ala variant in the peroxisome proliferator-activated receptor (PPAR) gamma2 on obesity and insulin resistance in Japanese Type 2 diabetic and healthy subjects	On obesity and insulin resistance<br><br>No full-text
Zhai B (2006) (98)	Interaction of adiponectin and peroxisome proliferaor activated receptor gamma 2 gene polymorphisms and their relationship with type 2 diabetes in population from Beijing communities	No full-text<br>(Article in Chinese)
Wang C (2004) (99)	Association of Pro12Ala mutation in peroxisome proliferator-activated receptor gamma 2 with obesity and diabetes in Chinese population	No full-text<br>No English<br>Article in Chinese
Fu M (2002) (100)	Association of Pro12Ala variant in peroxisome proliferator-activated receptor-gamma2 gene with type 2 diabetes mellitus	No full-text<br>No English<br>(Article in Chinese)
Dong Y (2004) (101)	Study of Pro12Ala polymorphism in peroxisome proliferator-activated receptor-72 gene with type 2 diabetes in Shanghai Han population [J]	No English<br>Article in Chinese
Bondar IA (2013) (102)	Rs7903146 variant of TCF7L2 gene and rs18012824 variant of PPARG2 gene (Pro12Ala) are associated with type 2 diabetes mellitus in Novosibirsk population	No English<br>Article in Russia
Gacka, M (2007) (103)	[The Pro12Ala polymorphism of the peroxisome proliferator-activated receptor gamma and immunological processes in patients with type 2 diabetes and insulin resistance]	No English<br>Article in Polish
Hara K (2003) (104)	[The role of PPARgamma in the onset of type 2 diabetes]	Review<br>No English<br>Article in Japanese
Sanghera DK (2008) (105)	Impact of nine common type 2 diabetes risk polymorphisms in Asian Indian Sikhs: PPARG2 (Pro12Ala), IGF2BP2, TCF7L2 and FTO variants confer a significant risk	Pilot Study<br>Duplicate
Kadowaki T (2002) (106)	The role of PPARg in high-fat diet-induced obesity and insulin resistance	Duplicate

Bouhaha R (2008) (107)	Effect of ENPP1/PC-1-K121Q and PPARg-Pro12Ala polymorphisms on the genetic susceptibility to T2D in the Tunisian population	Duplicate
Trombetta M (2013) (108)	PPARG2 Pro12Ala and ADAMTS9 rs4607103 as ‘‘insulin resistance loci’’ and ‘‘insulin secretion loci’’ in Italian individuals. The GENFIEV study and the Verona Newly Diagnosed Type 2 Diabetes Study (VNDS) 4	Duplicate
Zeggini E (2005) (109)	Examining the relationships between the Pro12Ala variant in PPARG and Type 2 diabetes-related traits in UK samples	Duplicate
Majithia AR (2014) (110)	Rare variants in PPARG with decreased activity in adipocyte differentiation are associated with increased risk of type 2 diabetes	Case-series<br>Duplicate
Ereqat S (2009) (111)	Impact of the pro12Ala polymorphism of the PPAR-gamma 2 gene on metabolic and clinical characteristics in the palestinian type 2 diabetic patients	Duplicate
Zouari KB (2005) (112)	The peroxisome proliferator activated receptorgamma2 (PPARgamma2) Pro12Ala variant: lack of association with type 2 diabetes in obese and non-obese Tunisian patients	Duplicate
Mori H (2001) (113)	The Pro12→ Ala substitution in PPAR-γ is associated with resistance to development of diabetes in the general population: possible involvement in impairment of insulin secretion in individuals with type 2 diabetes	Duplicate
Muller YL (2003) (114)	A functional variant in the peroxisome proliferator-activated receptor γ2 promoter is associated with predictors of obesity and type 2 diabetes in Pima Indians	Duplicate
Yen CJ (1997) (115)	Molecular scanning of the human peroxisome proliferator activated receptor γ (hPPARγ) gene in diabetic Caucasians: Identification of a Pro12Ala PPARγ2 missense mutation	Duplicate
Wu LSH (2009) / (116)	Association and interaction analyses of genetic variants in ADIPOQ, ENPP1, GHSR, PPARγ and TCF7L2 genes for diabetic nephropathy in a Taiwanese population with type 2 diabetes	Duplicate
Tavares V (2005) (117)	Association between Pro12Ala polymorphism of the PPARg 2 gene and insulin sensitivity in Brazilian patients with type-2 diabetes mellitus	Duplicate
Namvaran F (118)	Genotyping of peroxisome proliferator-activated receptor gamma (PPAR-γ) polymorphism (pro12ala) in Iranian population	Duplicate
Scacchi R (2007) (119)	An analysis of peroxisome proliferator-activated receptor gamma (PPAR-γ2) Pro12Ala polymorphism distribution and prevalence of type 2 diabetes mellitus (T2DM) in world populations in relation to dietary habits	Duplicate

Herrmann SM (2002) (120)	Peroxisome proliferator-activated receptor-(gamma)2 polymorphism Pro12Ala is associated with nephropathy in type 2 diabetes: The Berlin Diabetes Mellitus (BeDiaM) Study	Duplicate

Reference

1. Zmyslowska A, Szadkowska A, Mianowska B, Pietrzak I, Wyka K, Mlynarski W. The Pro12Ala PPARg2 gene polymorphism involves residual C-peptide secretion and BMI in type 1 diabetes. Pediatric Endocrinology, Diabetes and Metabolism. 2014;20(3):88-94.
2. Johansen A, Jensen DP, Bergholdt R, Mortensen HB, Pociot F, Nerup J, et al. IRS1, KCNJ11, PPARgamma2 and HNF-1alpha: do amino acid polymorphisms in these candidate genes support a shared aetiology between type 1 and type 2 diabetes? Diabetes, obesity & metabolism. 2006;8(1):75-82.
3. Wu LSH, Hsieh CH, Pei D, Hung YJ, Kuo SW, Lin E. Association and interaction analyses of genetic variants in ADIPOQ, ENPP1, GHSR, PPARγ and TCF7L2 genes for diabetic nephropathy in a Taiwanese population with type 2 diabetes. Nephrology Dialysis Transplantation. 2009;24(11):3360-6.
4. Zhang Y, Meng N, Lv Z, Li H, Qu Y. The gene polymorphisms of UCP1 but not PPAR gamma and TCF7L2 are associated with diabetic retinopathy in Chinese type 2 diabetes mellitus cases. Acta ophthalmologica. 2015;93(3):e223-9.
5. Pollex RL, Mamakeesick M, Zinman B, Harris SB, Hegele RA, Hanley AJG. Peroxisome proliferator-activated receptor gamma polymorphism Pro12Ala is associated with nephropathy in type 2 diabetes. J Diabetes Complications. 2007;21(3):166-71.
6. Franck N, Lanne T, Astrand O, Engvall J, Lindstrom T, Ostgren CJ, et al. Cardiovascular risk factors related to the PPAR gamma Pro12Ala polymorphism in patients with type 2 diabetes are gender dependent. Blood Pressure. 2012;21(2):122-7.
7. Herrmann SM, Ringel J, Wang JG, Staessen JA, Brand E. Peroxisome proliferator-activated receptor-gamma 2 polymorphism Pro12Ala is associated with nephropathy in type 2 diabetes - The Berlin Diabetes Mellitus (BeDiaM) Study. Diabetes. 2002;51(8):2653-7.
8. Lapice E, Monticelli A, Cocozza S, Pinelli M, Cocozza S, Bruzzese D, et al. The PPARgamma2 Pro12Ala variant is protective against progression of nephropathy in people with type 2 diabetes. Journal of translational medicine. 2015;13:85.
9. Kaur N, Vanita V. Association analysis of PPARgamma (p.Pro12Ala) polymorphism with type 2 diabetic retinopathy in patients from north India. Ophthalmic genetics. 2017;38(3):217-21.

10. Malecki MT, Cyganek K, Mirkiewicz-Sieradzka B, Wolkow PP, Wanic K, Skupien J, et al. Alanine variant of the Pro12Ala polymorphism of the PPARgamma gene might be associated with decreased risk of diabetic retinopathy in type 2 diabetes. Diabetes research and clinical practice. 2008;80(1):139-45.

11. Petrovic MG, Kunej T, Peterlin B, Dovc P, Petrovic D. Gly482Ser polymorphism of the peroxisome proliferator-activated receptor-gamma coactivator-1 gene might be a risk factor for diabetic retinopathy in Slovene population (Caucasians) with type 2 diabetes and the Pro12Ala polymorphism of the PPAR gamma gene is not. Diabetes-Metab Res Rev. 2005;21(5):470-4.

12. Caramori ML, Canani LH, Costa LA, Gross JL. The human peroxisome proliferator-activated receptor gamma2 (PPARgamma2) Pro12Ala polymorphism is associated with decreased risk of diabetic nephropathy in patients with type 2 diabetes. Diabetes. 2003;52(12):3010-3.

13. Ereqat S, Nasereddin A, Azmi K, Abdeen Z, Amin R. Impact of the Pro12Ala polymorphism of the PPAR-Gamma 2 gene on metabolic and clinical characteristics in the Palestinian type 2 diabetic patients. PPAR Res. 2009;2009:874126.

14. Hara M, Higaki Y, Taguchi N, Shinchi K, Morita E, Naito M, et al. Effect of the PPARG2 Pro12Ala polymorphism and clinical risk factors for diabetes mellitus on HbA1c in the Japanese general population. Journal of epidemiology. 2012;22(6):523-31.

15. Stumvoll M, Stefan N, Fritsche A, Madaus A, Tschritter O, Koch M, et al. Interaction effect between common polymorphisms in PPARgamma2 (Pro12Ala) and insulin receptor substrate 1 (Gly972Arg) on insulin sensitivity. Journal of molecular medicine (Berlin, Germany). 2002;80(1):33-8.

16. Stryjecki C, Peralta-Romero J, Alyass A, Karam-Araujo R, Suarez F, Gomez-Zamudio J, et al. Association between PPAR-gamma 2 Pro12Ala genotype and insulin resistance is modified by circulating lipids in Mexican children. Sci Rep. 2016;6.

17. Black MH, Wu J, Takayanagi M, Wang N, Taylor KD, Haritunians T, et al. Variation in PPARG is associated with longitudinal change in insulin resistance in Mexican Americans at risk for type 2 diabetes. The Journal of clinical endocrinology and metabolism. 2015;100(3):1187-95.

18. Tellechea ML, Aranguren F, Perez MS, Cerrone GE, Frechtel GD, Taverna MJ. Pro12Ala polymorphism of the peroxisome proliferatoractivated receptor-gamma gene is associated with metabolic syndrome and surrogate measures of insulin resistance in healthy men: interaction with smoking status. Circulation journal : official journal of the Japanese Circulation Society. 2009;73(11):2118-24.

19. Ahluwalia M, Evans M, Morris K, Currie C, Davies S, Rees A, et al. The influence of the Pro12Ala mutation of the PPAR-gamma receptor gene on metabolic and clinical characteristics in treatment-naive patients with type 2 diabetes. Diabetes, obesity & metabolism. 2002;4(6):376-8.

20. Zietz B, Barth N, Spiegel D, Schmitz G, Schölmerich J, Schäffler A. Pro12Ala polymorphism in the peroxisome proliferator-activated receptor-gamma2 (PPARgamma2) is associated with higher levels of total cholesterol and LDL-cholesterol in male caucasian type 2 diabetes patients. Experimental and clinical endocrinology & diabetes : official journal, German Society of Endocrinology [and] German Diabetes Association. 2002;110(2):60-6.

21. Nasereddin A, Ereqat S, Azmi K, Abdeen Z, Amin R. Impact of the pro12Ala polymorphism of the PPAR-gamma 2 gene on metabolic and clinical characteristics in the palestinian type 2 diabetic patients. PPAR Res. 2009.

22. Kadowaki T, Hara K, Kubota N, Tobe K, Terauchi Y, Yamauchi T, et al. The role of PPAR gamma in high-fat diet-induced obesity and insulin resistance. J Diabetes Complications. 2002;16(1):41-5.

23. Ostergard T, Ek J, Hamid Y, Saltin B, Pedersen OB, Hansen T, et al. Influence of the PPAR-gamma 2 Pro12Ala and ACE I/D polymorphisms on insulin sensitivity and training effects in healthy offspring of type 2 diabetic subjects. Horm Metab Res. 2005;37(2):99-105.

24. Villegas R, Williams SM, Gao YT, Long J, Shi J, Cai H, et al. Genetic variation in the peroxisome proliferator-activated receptor (PPAR) and peroxisome proliferator-activated receptor gamma co-activator 1 (PGC1) gene families and type 2 diabetes. Annals of human genetics. 2014;78(1):23-32.

25. Bonnycastle LL, Willer CJ, Conneely KN, Jackson AU, Burrill CP, Watanabe RM, et al. Common variants in maturity-onset diabetes of the young genes contribute to risk of type 2 diabetes in Finns. Diabetes. 2006;55(9):2534-40.

26. Zhu S, Liu Y, Wang X, Wu X, Zhu X, Li J, et al. Evaluation of the association between the PPARGC1A genetic polymorphisms and type 2 diabetes in Han Chinese population. Diabetes research and clinical practice. 2009;86(3):168-72.

27. Zhang SL, Lu WS, Yan L, Wu MC, Xu MT, Chen LH, et al. Association between peroxisome proliferator-activated receptor-gamma coactivator-1alpha gene polymorphisms and type 2 diabetes in southern Chinese population: role of altered interaction with myocyte enhancer factor 2C. Chin Med J (Engl). 2007;120(21):1878-85.

28. Shokouhi S, Haghani K, Borji P, Bakhtiyari S. Association between PGC-1alpha gene polymorphisms and type 2 diabetes risk: a case-control study of an Iranian population. Canadian journal of diabetes. 2015;39(1):65-72.

29. Sharma R, Matharoo K, Kapoor R, Bhanwer AJS. Association of PGC-1α gene with type 2 diabetes in three unrelated endogamous groups of North-West India (Punjab): a case-control and meta-analysis study. Molecular Genetics and Genomics. 2017:1-13.

30. Vasseur F, Helbecque N, Lobbens S, Vasseur-Delannoy V, Dina C, Clement K, et al. Hypoadiponectinaemia and high risk of type 2 diabetes are associated with adiponectin-encoding (ACDC) gene promoter variants in morbid obesity: evidence for a role of ACDC in diabesity. Diabetologia. 2005;48(5):892-9.

31. West NA, Haan MN, Morgenstern H. The PPAR-gamma Pro12Ala polymorphism and risk of cognitive impairment in a longitudinal study. Neurobiology of aging. 2010;31(5):741-6.

32. Thamer C, Haap M, Volk A, Maerker E, Becker R, Bachmann O, et al. Evidence for greater oxidative substrate flexibility in male carriers of the Pro 12 Ala polymorphism in PPARgamma2. Hormone and metabolic research = Hormon- und Stoffwechselforschung = Hormones et metabolisme. 2002;34(3):132-6.

33. Tan GD, Neville MJ, Liverani E, Humphreys SM, Currie JM, Dennis L, et al. The in vivo effects of the Pro12Ala PPARgamma2 polymorphism on adipose tissue NEFA metabolism: the first use of the Oxford Biobank. Diabetologia. 2006;49(1):158-68.

34. Takata N, Awata T, Inukai K, Watanabe M, Ohkubo T, Kurihara S, et al. Pro12Ala substitution in peroxisome proliferator-activated receptor gamma 2 is associated with low adiponectin concentrations in young Japanese men. Metabolism: clinical and experimental. 2004;53(12):1548-51.

35. Stefanski A, Majkowska L, Ciechanowicz A, Frankow M, Safranow K, Parczewski M, et al. Lack of association between the Pro12Ala polymorphism in PPAR-gamma 2 gene and body weight changes, insulin resistance and chronic diabetic complications in obese patients with type 2 diabetes. Arch Med Res. 2006;37(6):736-43.
36. Yates T, Davies MJ, Henson J, Edwardson C, Webb D, Bodicoat DH, et al. Effect of the PPARG2 Pro12Ala Polymorphism on Associations of Physical Activity and Sedentary Time with Markers of Insulin Sensitivity in Those with an Elevated Risk of Type 2 Diabetes. PloS one. 2015;10(5):e0124062.
37. Bendlova B, Vejrazkova D, Vcelak J, Lukasova P, Burkonova D, Kunesova M, et al. PPAR gamma 2 Pro12Ala polymorphism in relation to free fatty acids concentration and composition in lean healthy Czech individuals with and without family history of diabetes type 2. Physiol Res. 2008;57:S77-S90.
38. Verier C, Meirhaeghe A, Bokor S, Breidenassel C, Manios Y, Molnar D, et al. Breast-feeding modulates the influence of the peroxisome proliferator-activated receptor-gamma (PPARG2) Pro12Ala polymorphism on adiposity in adolescents: The Healthy Lifestyle in Europe by Nutrition in Adolescence (HELENA) cross-sectional study. Diabetes Care. 2010;33(1):190-6.
39. Namvaran F, Rahimi-Moghaddam P, Azarpira N. Genotyping of peroxisome proliferator-activated receptor gamma (PPAR-γ) polymorphism (pro12ala) in Iranian population. Journal of Research in Medical Sciences. 2011;16(3):291-6.
40. Lapice E, Monticelli A, Cocozza S, Pinelli M, Massimino E, Giacco A, et al. The combination of UCP3-55CT and PPARgamma2Pro12Ala polymorphisms affects BMI and substrate oxidation in two diabetic populations. Nutrition, metabolism, and cardiovascular diseases : NMCD. 2016;26(5):400-6.
41. Eftychi C, Howson JMM, Barratt BJ, Vella A, Payne F, Smyth DJ, et al. Analysis of the Type 2 Diabetes-Associated Single Nucleotide Polymorphisms in the Genes IRS1, KCNJ11, and PPARG2 in Type 1 Diabetes. Diabetes. 2004;53(3):870-3.
42. Tschritter O, Fritsche A, Stefan N, Haap M, Thamer C, Bachmann O, et al. Increased insulin clearance in peroxisome proliferator-activated receptor gamma(2) Pro12Ala. Metab-Clin Exp. 2003;52(6):778-83.
43. Poulsen P, Andersen G, Fenger M, Hansen T, Echwald SM, Volund A, et al. Impact of two common polymorphisms in the PPAR gamma gene on glucose tolerance and plasma insulin profiles in monozygotic and dizygotic twins - Thrifty genotype, thrifty phenotype, or both? Diabetes. 2003;52(1):194-8.
44. Lamri A, Abi Khalil C, Jaziri R, Velho G, Lantieri O, Vol S, et al. Dietary fat intake and polymorphisms at the PPARG locus modulate BMI and type 2 diabetes risk in the D.E.S.I.R. prospective study. International journal of obesity (2005). 2012;36(2):218-24.
45. Estivalet AAF, Leiria LB, Dora JM, Rheinheimer J, Boucas AP, Maia AL, et al. D2 Thr92Ala and PPAR gamma 2 Pro12Ala Polymorphisms Interact in the Modulation of Insulin Resistance in Type 2 Diabetic Patients. Obesity. 2011;19(4):825-32.
46. Ruchat SM, Weisnagel SJ, Vohl MC, Rankinen T, Bouchard C, Perusse L. Evidence for Interaction between PPARG Pro12Ala and PPARGC1A Gly482Ser Polymorphisms in Determining Type 2 Diabetes Intermediate Phenotypes in Overweight Subjects. Exp Clin Endocrinol Diabet. 2009;117(9):455-9.

47. Lee BC, Lee HJ, Chung JH. Peroxisome proliferator-activated receptor-gamma 2 Pro12Ala polymorphism is associated with reduced risk for ischemic stroke with type 2 diabetes. Neurosci Lett. 2006;410(2):141-5.
48. Kozarova M, Javorsky M, Stancakova A, Dobrikova M, Habalova V, Klimcakova L, et al. Relationship of five type 2 diabetes candidate gene polymorphisms to the age at diagnosis of diabetes in the Slovakian population. Bratislavske lekarske listy. 2010;111(3):150-2.
49. Okazawa H, Mori H, Tamori Y, Araki S, Niki T, Masugi J, et al. No Coding Mutations Are Detected in the Peroxisome Proliferator-Activated Receptor-smgamma. gif (938 bytes) Gene in Japanese Patients with Lipoatrophic Diabetes. Diabetes. 1997;46(11).
50. Vigouroux C, Fajas L, Khallouf E, Meier M, Gyapay G, Lascols O, et al. Human peroxisome proliferator-activated receptor-gamma2: genetic mapping, identification of a variant in the coding sequence, and exclusion as the gene responsible for lipoatrophic diabetes. Diabetes. 1998;47(3):490-2.
51. Andrulionyte L, Zacharova J, Chiasson JL, Laakso M. Common polymorphisms of the PPAR-gamma 2 (Pro12Ala) and PGC-1 alpha (Gly482Ser) genes are associated with the conversion from impaired glucose tolerance to type 2 diabetes in the STOP-NIDDM trial. Diabetologia. 2004;47(12):2176-84.
52. Andrulionyte L, Peltola P, Chiasson JL, Laakso M. Single nucleotide polymorphisms of PPARD in combination with the Gly482Ser substitution of PGC-1A and the Pro12Ala substitution of PPARG2 predict the conversion from impaired glucose tolerance to type 2 diabetes: the STOP-NIDDM trial. Diabetes. 2006;55(7):2148-52.
53. Kilpelainen TO, Lakka TA, Laaksonen DE, Lindstrom J, Eriksson JG, Valle TT, et al. SNPs in PPARG associate with type 2 diabetes and interact with physical activity. Medicine and science in sports and exercise. 2008;40(1):25-33.
54. Lindi VI, Uusitupa MIJ, Lindstrom J, Louheranta A, Eriksson JG, Valle TT, et al. Association of the Pro12Ala polymorphism in the PPAR-gamma 2 gene with 3-year incidence of type 2 diabetes and body weight change in the Finnish Diabetes Prevention Study. Diabetes. 2002;51(8):2581-6.
55. Florez JC, Jablonski KA, Sun MW, Bayley N, Kahn SE, Shamoon H, et al. Effects of the type 2 diabetes-associated PPARG P12A polymorphism on progression to diabetes and response to troglitazone. The Journal of Clinical Endocrinology & Metabolism. 2007;92(4):1502-9.
56. Chung-Jen Y, Beamer BA, Negri C, Silver K, Brown KA, Yarnall DP, et al. Molecular scanning of the human peroxisome proliferator activated receptor γ (hPPARγ) gene in diabetic Caucasians: Identification of a Pro12Ala PPARγ2 missense mutation. Biochemical and Biophysical Research Communications. 1997;241(2):270-4.
57. Scacchi R, Pinto A, Rickards O, Pacella A, De Stefano GF, Cannella C, et al. An analysis of peroxisome proliferator-activated receptor gamma (PPAR-gamma 2) Pro12Ala polymorphism distribution and prevalence of type 2 diabetes mellitus (T2DM) in world populations in relation to dietary habits. Nutrition, metabolism, and cardiovascular diseases : NMCD. 2007;17(9):632-41.
58. Bego T, Dujic T, Mlinar B, Semiz S, Malenica M, Prnjavorac B, et al. Association of PPARG and LPIN1 gene polymorphisms with metabolic syndrome and type 2 diabetes. Med Glas (Zenica). 2011;8(1):76-83.

59. Celi FS, Shuldiner AR. The role of peroxisome proliferator-activated receptor gamma in diabetes and obesity. Current diabetes reports. 2002;2(2):179-85.

60. Tonjes A, Stumvoll M. The role of the Pro12Ala polymorphism in peroxisome proliferator-activated receptor gamma in diabetes risk. Current opinion in clinical nutrition and metabolic care. 2007;10(4):410-4.

61. Radha V, Mohan V. Genetic predisposition to type 2 diabetes among Asian Indians. The Indian journal of medical research. 2007;125(3):259-74.

62. Stumvoll M, Haring H. The peroxisome proliferator--activated receptor-(gamma)2 pro12Ala polymorphism. Diabetes. 2002;51(8):2341-7.

63. Dong C, Zhou H, Shen C, Yu L-G, Ding Y, Zhang Y-H, et al. Role of peroxisome proliferator-activated receptors gene polymorphisms in type 2 diabetes and metabolic syndrome. World journal of diabetes. 2015;6(4):654.

64. Tong JY, Huang N, Wang L, Yi YS, Pan XH, Lu Y. [Relationship between PPARgamma2 Pro12Ala polymorphism and type 2 diabetes mellitus in Chinese Han population: a Meta-analysis]. Zhonghua yu fang yi xue za zhi [Chinese journal of preventive medicine]. 2012;46(4):359-63.

65. Guo WL, Tang Y, Han XY, Ji LN. [Meta-analysis of the association of Pro12Ala polymorphism of peroxisome proliferator activated receptor gamma gene with type 2 diabetes in Chinese Han population]. Zhongguo yi xue ke xue yuan xue bao Acta Academiae Medicinae Sinicae. 2011;33(6):593-9.

66. Huguenin GVB, Rosa G. The Ala allele in the PPAR-gamma 2 gene is associated with reduced risk of type 2 diabetes mellitus in Caucasians and improved insulin sensitivity in overweight subjects. Br J Nutr. 2010;104(4):488-97.

67. Ludovico O, Pellegrini F, Di Paola R, Minenna A, Mastroianno S, Cardellini M, et al. Heterogeneous Effect of Peroxisome Proliferator-activated Receptor γ2 Ala12 Variant on Type 2 Diabetes Risk. Obesity. 2007;15(5):1076-81.

68. Altshuler D, Hirschhorn JN, Klannemark M, Lindgren CM, Vohl MC, Nemesh J, et al. The common PPARgamma Pro12Ala polymorphism is associated with decreased risk of type 2 diabetes. Nature genetics. 2000;26(1):76-80.

69. Deeb SS, Fajas L, Nemoto M, Pihlajamaki J, Mykkanen L, Kuusisto J, et al. A Pro12Ala substitution in PPARgamma2 associated with decreased receptor activity, lower body mass index and improved insulin sensitivity. Nature genetics. 1998;20(3):284-7.

70. Barroso I, Gurnell M, Crowley V, Agostini M, Schwabe J, Soos M, et al. Dominant negative mutations in human PPARγ associated with severe insulin resistance, diabetes mellitus and hypertension. Nature. 1999;402(6764):880.

71. Wakil SM, Al-Rubeaan K, Alsmadi O, Imtiaz F, Carroll P, Rajab M, et al. The Peroxisome Proliferator–Activated Receptor-γ2 P12A Polymorphism and Type 2 Diabetes in an Arab Population. Diabetes Care. 2006;29(1):171-2.

72. Nelson TL, Fingerlin TE, Moss LK, Barmada MM, Ferrell RE, Norris JM. Association of the peroxisome proliferator-activated receptor gamma gene with type 2 diabetes mellitus varies by physical activity among non-Hispanic whites from Colorado. Metab-Clin Exp. 2007;56(3):388-93.

73. Black MH, Fingerlin TE, Allayee H, Zhang W, Xiang AH, Trigo E, et al. Evidence of interaction between PPARG2 and HNF4A contributing to variation in insulin sensitivity in Mexican Americans. Diabetes. 2008;57(4):1048-56.

74. Lyssenko V, Jonsson A, Almgren P, Pulizzi N, Isomaa B, Tuomi T, et al. Clinical risk factors, DNA variants, and the development of type 2 diabetes. The New England journal of medicine. 2008;359(21):2220-32.

75. Park SE, Lee WY, Oh KW, Baek KH, Yoon KH, Kang MI, et al. Impact of common type 2 diabetes risk gene variants on future type 2 diabetes in the non-diabetic population in Korea. Journal of human genetics. 2012;57(4):265-8.

76. Lin Y, Li P, Cai L, Zhang B, Tang X, Zhang X, et al. Association study of genetic variants in eight genes/loci with type 2 diabetes in a Han Chinese population. BMC medical genetics. 2010;11:97.

77. Hu C, Zhang R, Wang C, Wang J, Ma X, Lu J, et al. PPARG, KCNJ11, CDKAL1, CDKN2A-CDKN2B, IDE-KIF11-HHEX, IGF2BP2 and SLC30A8 are associated with type 2 diabetes in a chinese population. PloS one. 2009;4(10):e7643.

78. Gamboa-Meléndez MA, Huerta-Chagoya A, Moreno-Macías H, Vázquez-Cárdenas P, Ordóñez-Sánchez ML, Rodríguez-Guillén R, et al. Contribution of common genetic variation to the risk of type 2 diabetes in the Mexican Mestizo population. Diabetes. 2012:DB_110550.

79. Hanson RL, Rong R, Kobes S, Muller YL, Weil EJ, Curtis JM, et al. Role of Established Type 2 Diabetes-Susceptibility Genetic Variants in a High Prevalence American Indian Population. Diabetes. 2015;64(7):2646-57.

80. Chauhan G, Spurgeon CJ, Tabassum R, Bhaskar S, Kulkarni SR, Mahajan A, et al. Impact of common variants of PPARG, KCNJ11, TCF7L2, SLC30A8, HHEX, CDKN2A, IGF2BP2 and CDKAL1 on the risk of type 2 diabetes in 5164 Indians. Diabetes. 2010.

81. Voight BF, Scott LJ, Steinthorsdottir V, Morris AP, Dina C, Welch RP, et al. Twelve type 2 diabetes susceptibility loci identified through large-scale association analysis. Nature genetics. 2010;42(7):579.

82. Diabetes Epidemiology; Pro12Ala variant of PPARG2 gene plus high intake of oleic acid is linked to type 2 diabetes. Obesity & Diabetes Week. 2006 2006 Oct 23.

83. Nutritional and Metabolic Diseases and Conditions; New Type 2 Diabetes Study Results from National Research Council Described (The combination of UCP3-55CT and PPAR gamma 2Pro12Ala polymorphisms affects BMI and substrate oxidation in two diabetic populations). Diabetes Week. 2016 2016 Jun 13.

84. Lapice E, Cocozza S, Riccardi G, Vaccaro O. Comment on: Zhang et al. Peroxisome proliferator-activated receptor gamma polymorphism Pro12Ala is associated with nephropathy in type 2 diabetes: evidence from meta-analysis of 18 studies. Diabetes Care 2012;35:1388-1393. Diabetes Care. 2013;36(1):e18.

85. Shah AY, Sheth J, Trivedi S, Nabar N, Shah N, Thakor P, et al. Interplay of Pparγ2 and adrβ3 allelic polymorphism and metabolic factors conferring poor hemoglobin glycation and increased insulin resistance: A western indian study. Endocrine Reviews. 2016;37(2).

86. Osman NA, NasrAllah MM, Kamal MM, Ahmed AI. The association between diabetic nephropathy and polymorphisms of PPAR PRO12ALA and CCR5 32 genes in type 2 diabetes. Nephrol Dial Transplant. 2013;28:i382-i3.

87. Yang YZ, Feng K, Wang D. Correlation Analysis of Pro12A1a Polymorphism in PPAR gamma 2 Gene with Susceptibility to Diabetic Nephropathy in Type 2 Diabetes Mellitus. Diabetes. 2010;59:A590-A.

88. Namvaran F, Azarpira N, Rahimi-Moghaddam P. PEROXISOME PROLIFERATOR-ACTIVATED RECEPTOR Gamma POLYMORPHISM IN IRANIAN POPULATION: RELATION WITH INSULIN RESISTANCE AND RESPONSE TO PIOGLITAZONE IN DIABETES TYPE 2. Basic Clin Pharmacol Toxicol. 2011;109:88-.

89. Mato EPM, Sobngwi E, Sontsa OD, Ndonwi EN, Fokeng MG, Pokam PF, et al. No Association between Pro12Ala polymorphism in Peroxisome Proliferator Activated Receptor Gamma 2 gene and type 2 diabetes in a Cameroonian population. Diabetes research and clinical practice. 2014;103:S37-S8.

90. Watanabe R, Erdos M, Douglas J, Valle T, Tuomilehto J, Collins F, et al. Association of the peroxisome proliferator-activated receptory-gamma 2) (PPAR-gamma 2) PRO12ALA variant with type 2 diabetes-related phenotypes in a large Finnish cohort. Diabetes. 2000;49:A206-A.

91. Andersen G, Ek J, Urhammer SA, Eiberg H, Borch-Johnsen K, Hansen T, et al. The Pro12Ala polymorphism of the peroxisome proliferator-activated receptor-gamma 2 is not associated with type 2 diabetes. Diabetologia. 2000;43:A87-A.

92. Erdos MR, Douglas JA, Watanabe RM, Braun A, Oeth P, Johnston C, et al. The PPAR-gamma 2 Pro12Ala variant: association with type 2 diabetes, trait differences, and interaction with the beta(3)-adrenergic receptor. Am J Hum Genet. 2000;67(4):357-.

93. Ek J, Andersen G, Urhammer SA, Hansen L, Carstensen B, Borch-Johnsen K, et al. Studies of the Pro12Ala polymorphism of the peroxisome proliferator-activated receptor-gamma2 (PPAR-gamma2) gene in relation to insulin sensitivity among glucose tolerant caucasians. Diabetologia. 2001;44(9):1170-6.

94. Donnarumma G, Lapice E, Giacchetti M, Pinelli M, Nosso G, Romano G, et al. Absence of association between Pro12Ala polymorphism of the PPARγ2 gene and diabetes. Giornale Italiano di Diabetologia e Metabolismo. 2006;26(3):105-9.

95. Zhang A, Zhang M, Zhang J, Yu Y, Xie J. The influence of the Pro12Ala mutation of PPARgamma2 receptor gene on beta-cells restoration and insulin resistance in type 2 diabetes with hypertension. Journal of Huazhong University of Science and Technology Medical sciences = Hua zhong ke ji da xue xue bao Yi xue Ying De wen ban = Huazhong keji daxue xuebao Yixue Yingdewen ban. 2005;25(6):648-50.

96. Azab MM, Abdel-Azeez HA, Zanaty MF, El Alawi SM. Peroxisome Proliferator Activated Receptor gamma 2 Pro12Ala Gene Polymorphism in Type 2 Diabetes and its Relationship with Diabetic Nephropathy. Clin Lab. 2014;60(5):743-9.

97. Kawasaki I, Tahara H, Emoto M, Shoji T, Shioji A, Okuno Y, et al. Impact of Prol2Ala variant in the peroxisome proliferator-activated receptor (PPAR) gamma2 on obesity and insulin resistance in Japanese Type 2 diabetic and healthy subjects. Osaka city medical journal. 2002;48(1):23-8.

98. Zhai B, Ye L, Liu J, Liu J. Interaction of adiponectin and peroxisome proliferaor activated receptor gamma 2 gene polymorphisms and their relationship with type 2 diabetes in population from Beijing communities. Chin J Clin Rehabil. 2006;10:28-31.
99. Wang C, Zhai F, Chi Y, Wang G. Association of Pro12Ala mutation in peroxisome proliferator-activated receptor gamma 2 with obesity and diabetes in Chinese population. Wei sheng yan jiu= Journal of hygiene research. 2004;33(3):317-20.
100. Fu M, Chen H, Li X, Li J, Wu B, Cheng L, et al. Association of Pro12Ala variant in peroxisome proliferator-activated receptor-gamma2 gene with type 2 diabetes mellitus. Zhonghua yi xue yi chuan xue za zhi= Zhonghua yixue yichuanxue zazhi= Chinese journal of medical genetics. 2002;19(3):234-8.
101. DONG Y, LI G, LUO T-h, WU G, HUANG W, LUO M. Study of Pro12Ala polymorphism in peroxisome proliferator-activated receptor-72 gene with type 2 diabetes in Shanghai Han population [J]. Acta Universitatis Medicinalis Secondae Shanghai. 2004;5:009.
102. Bondar IA, Filipenko ML, Shabel'nikova OY, Sokolova EA. Rs7903146 variant of TCF7L2 gene and rs18012824 variant of PPARG2 gene (Pro12Ala) are associated with type 2 diabetes mellitus in Novosibirsk population. Diabetes Mellitus. 2013(4):17-22.
103. Gacka M, Bednarska-Chabowska D, Dobosz T, Szymaniec S, Jakobsche U, Lebioda A, et al. [The Pro12Ala polymorphism of the peroxisome proliferator-activated receptor gamma and immunological processes in patients with type 2 diabetes and insulin resistance]. Przeglad lekarski. 2007;64(6):393-7.
104. Hara K, Yamauchi T, Kubota N, Tobe K, Yamazaki T, Nagai R, et al. [The role of PPARgamma in the onset of type 2 diabetes]. Nihon yakurigaku zasshi Folia pharmacologica Japonica. 2003;122(4):317-24.
105. Sanghera DK, Ortega L, Han S, Singh J, Ralhan SK, Wander GS, et al. Impact of nine common type 2 diabetes risk polymorphisms in Asian Indian Sikhs: PPARG2 (Pro12Ala), IGF2BP2, TCF7L2 and FTO variants confer a significant risk. BMC medical genetics. 2008;9:59.
106. Kadowaki T, Hara K, Kubota N, Tobe K, Terauchi Y, Yamauchi T, et al. The role of PPARγ in high-fat diet-induced obesity and insulin resistance. J Diabetes Complications. 2002;16(1):41-5.
107. Bouhaha R, Meyre D, Kamoun HA, Ennafaa H, Vaillant E, Sassi R, et al. Effect of ENPP1/PC-1-K121Q and PPAR gamma-Pro12Ala polymorphisms on the genetic susceptibility to T2D in the Tunisian population. Diabetes research and clinical practice. 2008;81(3):278-83.
108. Trombetta M, Bonetti S, Boselli ML, Miccoli R, Trabetti E, Malerba G, et al. PPARG2 Pro12Ala and ADAMTS9 rs4607103 as "insulin resistance loci" and "insulin secretion loci" in Italian individuals. The GENFIEV study and the Verona Newly Diagnosed Type 2 Diabetes Study (VNDS) 4. Acta diabetologica. 2013;50(3):401-8.
109. Zeggini E, Parkinson JRC, Halford S, Owen K, Walker M, Hitman G, et al. Examining the relationships between the Pro12Ala variant in PPARG and Type 2 diabetes-related traits in UK samples. Diabetic Med. 2005;22(12):1696-700.
110. Majithia AR, Flannick J, Shahinian P, Guo M, Bray M-A, Fontanillas P, et al. Rare variants in PPARG with decreased activity in adipocyte differentiation are associated with increased risk of type 2 diabetes. Proceedings of the National Academy of Sciences. 2014;111(36):13127-32.

111. Ereqat S, Nasereddin A, Azmi K, Abdeen Z, Amin R. Impact of the Pro12Ala polymorphism of the PPAR-Gamma 2 gene on metabolic and clinical characteristics in the Palestinian type 2 diabetic patients. PPAR Res. 2009;2009.

112. Zouari KB, Chouchane L, Jellouli K, Cherif S, Haddad S, Gabbouj S, et al. The peroxisome proliferator activated receptorgamma2 (PPARgamma2) Pro12Ala variant: lack of association with type 2 diabetes in obese and non obese Tunisian patients. Diabetes & metabolism. 2005;31(2):119-23.

113. Mori H, Ikegami H, Kawaguchi Y, Seino S, Yokoi N, Takeda J, et al. The Pro12→ Ala substitution in PPAR-γ is associated with resistance to development of diabetes in the general population: possible involvement in impairment of insulin secretion in individuals with type 2 diabetes. Diabetes. 2001;50(4):891-4.

114. Muller YL, Bogardus C, Beamer BA, Shuldiner AR, Baier LJ. A functional variant in the peroxisome proliferator-activated receptor γ2 promoter is associated with predictors of obesity and type 2 diabetes in Pima Indians. Diabetes. 2003;52(7):1864-71.

115. Yen C-J, Beamer BA, Negri C, Silver K, Brown KA, Yarnall DP, et al. Molecular scanning of the human peroxisome proliferator activated receptor γ (hPPARγ) gene in diabetic Caucasians: identification of a Pro12Ala PPARγ2 missense mutation. Biochemical and biophysical research communications. 1997;241(2):270-4.

116. Wu LSH, Hsieh CH, Pei D, Hung YJ, Kuo SW, Lin E. Association and interaction analyses of genetic variants in ADIPOQ, ENPP1, GHSR, PPAR gamma and TCF7L2 genes for diabetic nephropathy in a Taiwanese population with type 2 diabetes. Nephrol Dial Transplant. 2009;24(11):3360-6.

117. Tavares V, Hirata RDC, Rodrigues AC, Monte O, Salles JEN, Scalissi N, et al. Association between Pro12Ala polymorphism of the PPAR-gamma 2 gene and insulin sensitivity in Brazilian patients with type-2 diabetes mellitus. Diabetes Obes Metab. 2005;7(5):605-11.

118. Namvaran F, Rahimi-Moghaddam P, Azarpira N. Genotyping of peroxisome proliferator-activated receptor gamma (PPAR-γ) polymorphism (Pro12Ala) in Iranian population. Journal of research in medical sciences: the official journal of Isfahan University of Medical Sciences. 2011;16(3):291.

119. Scacchi R, Pinto A, Rickards O, Pacella A, De Stefano G, Cannella C, et al. An analysis of peroxisome proliferator-activated receptor gamma (PPAR-γ2) Pro12Ala polymorphism distribution and prevalence of type 2 diabetes mellitus (T2DM) in world populations in relation to dietary habits. Nutrition, Metabolism and Cardiovascular Diseases. 2007;17(9):632-41.

120. Stefan-Martin H, Ringel J, Wang J-G, Staessen JA, Brand E. Peroxisome proliferator-activated receptor-(gamma)2 polymorphism Pro12Ala is associated with nephropathy in type 2 diabetes: The Berlin Diabetes Mellitus (BeDiaM) Study. Diabetes. 2002;51(8):2653-7.
